# Universals of word order reflect optimization of grammars for efficient communication

**DOI:** 10.1073/pnas.1910923117

**Published:** 2020-01-21

**Authors:** Michael Hahn, Dan Jurafsky, Richard Futrell

**Affiliations:** ^a^Department of Linguistics, Stanford University, Stanford, CA 94305;; ^b^Department of Language Science, University of California, Irvine, CA 92697

**Keywords:** language universals, language processing, computational linguistics

## Abstract

The universal properties of human languages have been the subject of intense study across the language sciences. We report computational and corpus evidence for the hypothesis that a prominent subset of these universal properties—those related to word order—result from a process of optimization for efficient communication among humans, trading off the need to reduce complexity with the need to reduce ambiguity. We formalize these two pressures with information-theoretic and neural-network models of complexity and ambiguity and simulate grammars with optimized word-order parameters on large-scale data from 51 languages. Evolution of grammars toward efficiency results in word-order patterns that predict a large subset of the major word-order correlations across languages.

## Significance

Human languages share many grammatical properties. We show that some of these properties can be explained by the need for languages to offer efficient communication between humans given our cognitive constraints. Grammars of languages seem to find a balance between two communicative pressures: to be simple enough to allow the speaker to easily produce sentences, but complex enough to be unambiguous to the hearer, and this balance explains well-known word-order generalizations across our sample of 51 varied languages. Our results offer quantitative and computational evidence that language structure is dynamically shaped by communicative and cognitive pressures.

Understanding what is universal and what varies across human languages is a central goal of linguistics. Across theoretical paradigms, linguists have hypothesized that language is shaped by efficiency in computation (1–4) and communication (5–10). However, formalizing how these pressures explain specific grammatical universals has proved difficult. Here, we pair computational models that measure the communicative efficiency of grammars with a simulation framework for finding optimal grammars and show that the most efficient grammars also exhibit a large class of language universals.

**Fig. 1.**
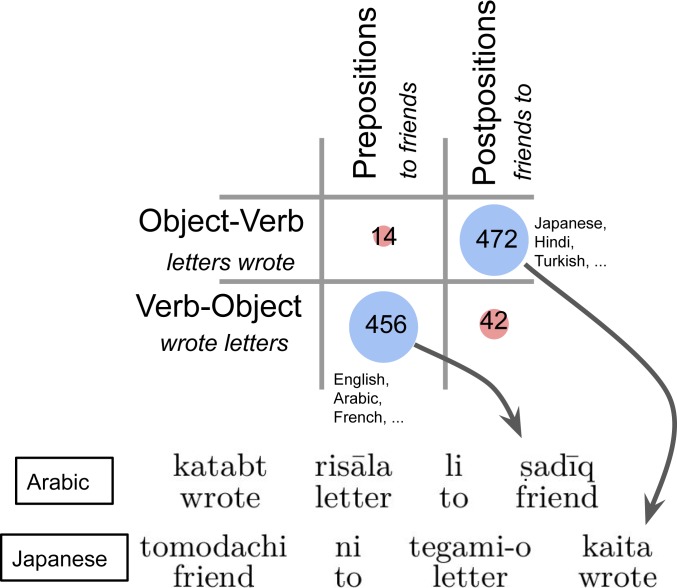
One word-order correlation. Languages can order the object after (Arabic) or before (Japanese) the verb and have prepositions (Arabic) or postpositions (Japanese). For each combination, we indicate how many languages satisfy it, as documented in the World Atlas of Language Structures (12). Combinations on the diagonal are vastly more common than off-diagonal ones.

The language universals we study are the well-known Greenberg universals of word order (11). Human languages vary in the order in which they express information. Consider Fig. 1, showing a sentence in Arabic (*Top*) and Japanese (*Bottom*), both translating to “I wrote a letter to a friend.” Both sentences contain a verb meaning “wrote,” a noun expressing the object “letter,” and a phrase translating to “to a friend.” However, the order of these words is entirely different in the two languages: the verb stands at the beginning in Arabic and at the end in Japanese. Arabic expresses “to” by a preposition (preceding the noun “friend”); Japanese uses a postposition (following it).

However, this variation reflects a deep and stable regularity: while languages ordering the objects before (Japanese) or after (Arabic) the verb are approximately equally common around the world, this is strongly correlated with the occurrence of pre- or postpositions (Fig. 1, *Top*): languages ordering their objects the way Japanese does have postpositions; languages ordering them as Arabic does have prepositions.

This generalization lies in a group of language universals originally documented by Greenberg (11), known as word-order correlations. These describe correlations between the relative positions of different types of expressions across languages. The example above documents that the position of the object (“letter”) relative to the verb is correlated with the position of the adposition (“to”). Greenberg also found that the order of verb and object is correlated with other aspects of a language’s word order (Table 1), such as the order of verb and adpositional phrase (“wrote – to friend” in Arabic vs. “friend to – wrote” in Japanese) and that of noun and genitive (“book – of friend” in Arabic, “friend of – book” in Japanese).

**Table 1.** Greenberg word-order correlations, exemplified by Arabic (left) and Japanese (right) examples

	Arabic (English, …)		Japanese (Turkish, …)	
Correlation no.	Correlates with verb	Correlates with object	Correlates with object	Correlates with verb
	kataba	risāla	tegami-o	kaita
	*wrote*	*letter*	*letter*	*wrote*
①	li	ṣadīq	tomodachi	ni
	*to*	*a friend*	*friend*	*to*
②	kāna	ṣadīq	tomodachi	datta
	*was*	*a friend*	*friend*	*was*
③	sawfa	yaktub	kak-	-udesho
	*will*	*write*	*write*	*will*
④	ṣadīq	John	John no	tomodachi
	*friend*	*of John*	*John of*	*friend*
⑤	kutub	taqra’uhā	anata-ga yonda	hon
	*books*	*that you read*	*that you read*	*book*
⑥	’an	tuṣil	toochaku suru	koto
	*that*	*she arrives*	*arrives*	*that*
⑦	dhahabt	’ilā lmadrasa	gakkoo ni	itta
	*went*	*to school*	*school to*	*went*
⑧	’urīd	’an ’ughādir	ik-	-itai
	*wants*	*to leave*	*to go*	*want*

Across the world, the orders of different constituents are strikingly correlated with that of verb and object. Selection is based on a more recent typological study by Dryer (13), restricted to those correlations that are annotated in available corpus data. See *SI Appendix*, section S1 for more on Greenberg correlations.

Supported by languages on all continents, these correlations are among the language universals with the strongest empirical support. Importantly, their validity is also independent from specific assumptions about theories of grammar.

Explaining these patterns has been an important aim of linguistic research since Greenberg’s seminal study (4, 13–19). Prominent among this research is the argument that language universals arise for functional reasons: that is, because they make human communication and language processing maximally efficient, and regularities across languages hold because these efficiency constraints are rooted in general principles of communication and cognition (e.g., refs. 4, 5, 8, 9, and 20–26). Under this view, the various human languages represent multiple solutions to the problem of efficient information transfer given human cognitive constraints.

In an early and influential functional framework, Zipf (5) argued that language optimizes a tradeoff between two pressures: to reduce complexity and to reduce ambiguity. What Zipf called the “Force of Unification” is a pressure to reduce the complexity of the language by reducing the number of distinctions made in the language, in order to make production and processing as easy as possible. The countervailing “Force of Diversification” favors languages that provide different utterances for different meanings, so that the listener can unambiguously identify the meaning from the utterance. These two forces act in opposing directions: producing and processing simple utterances incurs little cost, but more complex and diverse utterances are required to provide enough information. The idea that many properties of language arise from the tension between these two pressures has a long and fruitful history in linguistics (20, 23, 27–29).

Recent work has drawn on information theory to computationally test this “dual pressures” idea in various domains of language, showing that it predicts both basic statistical properties of languages (30, 31) and language evolution (8) and sophisticated aspects of language, such as pragmatic inference (32), and the distribution of color words (33) and kinship categories (34) across many languages. While it has been suggested that the dual pressure should also apply to grammar (23), testing these accounts is more difficult, as this requires large amounts of data representative of language use across languages, computational methods for estimating the efficiency of entire languages, and a simulation methodology for comparing different possible grammars.

In this work, we address these challenges by combining large-scale text data from 51 languages with machine-learning techniques to estimate both aspects of the communicative efficiency of grammar: complexity and ambiguity. We use machine-learning models based on neural networks to model the evolution of grammars toward efficiency. We apply this approach to the problem of explaining Greenberg word-order correlation universals.

In Study 1, we compare the word order of actual grammars of 51 languages with alternative “counterfactual” grammars parameterized by different word orders. We use our model to measure the communicative efficiency of each possible grammar, showing that the grammars of real languages are more efficient than alternative grammars. The fact that real grammars lie at the Pareto frontier of the efficiency space of possible grammars suggests that the word order of languages has evolved to optimize communicative efficiency.

In Study 2, we test whether efficiency optimization accounts for the Greenberg word-order correlations. For each of the 51 languages, we create hypothetical grammars optimized for efficiency. We then test statistically whether these optimized grammars exhibit the Greenberg correlations, using a Bayesian mixed-effects logistic regression to control for language and language family. Efficiency optimization indeed predicts all eight Greenberg correlations. Our results show that general properties of efficient communication can give rise to these universal word-order properties of human language.

## Grammars and Grammar Data

**Fig. 2.**
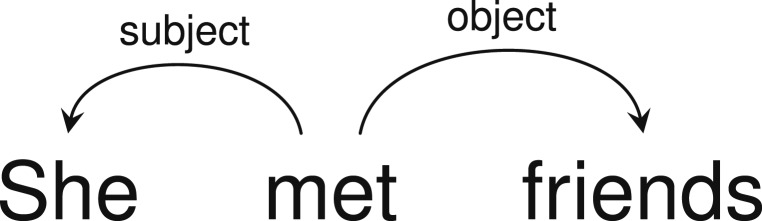
An English sentence with annotated syntactic relations.

Following a long tradition in theoretical and computational linguistics, we formalize the grammatical structure of languages using dependency trees (35–39). This linguistic formalism represents grammatical dependencies as directed arcs between syntactically related words, annotated with grammatical relations like subject or object (Fig. 2). While syntactic formalisms vary, the dependency grammar community has an agreed representation format that has been used to annotate corpora of text from dozens of languages (40), and there are computational methods for deriving such representations from other standard linguistic formalisms (41).

Our models require a sample of syntactic structures as actually used by speakers across different languages, for which we draw on the recent Universal Dependencies project (40), which has collected and created syntactic annotations for several dozens of different languages; 51 languages had sufficient data for our purposes. These corpora represent a typologically and genetically diverse group of languages. We obtained a total of 11.7 million words in 700,000 sentences annotated with syntactic structures, with a median of 117,000 words and 7,000 sentences for each individual language.

## Study 1: Efficiency of Languages

We first ask whether the grammars of human languages reflect optimization for efficiency of communication. To do this, we compare the efficiency of the actual grammars of the 51 languages from the Universal Dependencies datasets to randomly constructed baseline grammars.

**Fig. 3.**
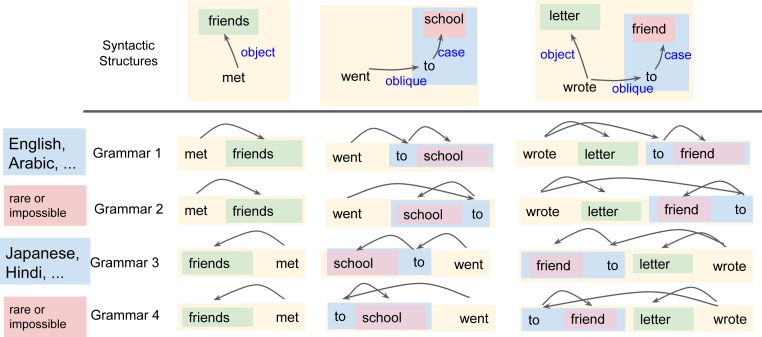
Grammars define consistent ordering rules for syntactic structures. Here, Grammars 1 and 2 order the object after the verb, and Grammars 3 and 4 order the object before the verb. Grammars 1 and 3 conform to the Greenberg correlations and are common around the world; Grammars 2 and 4 are rare or impossible.

The grammars of natural languages specify how the different words in a syntactic structure are ordered into a sentence, i.e., a string of words (42). This is illustrated in Fig. 3: we show how four different grammars order objects, adpositional phrases, and adpositions. For instance, Grammar 1—corresponding to Arabic in Fig. 1—orders objects (“friends,” “letter”) after verbs and has prepositions (“to friend”). Grammar 2 orders objects after verbs but has postpositions (“friend – to”). Grammars 3 and 4 place the object before the verb, and one of them (Grammar 3) corresponds to Japanese order.

Beyond the syntactic relations exemplified in Fig. 3, human languages have further types of syntactic relations. The Universal Dependencies project, the source of our data, defines a total of 37 syntactic relations. We adopt a variant of the grammar model developed by Gildea and coworkers (43–45): a grammar assigns a weight from [−1,1] to each of these 37 syntactic relations and orders words according to the weights assigned to their relations (see Materials and Methods for details).

Given a large database of sentences annotated with syntactic structures (such as those at the top of Fig. 3), obtained from a corpus of some real language L, we can apply a grammar to reorder the structures in the database into a dataset of counterfactual sentences belonging to a hypothetical language defined by that grammar (Fig. 3). This hypothetical language has identical syntactic structures and grammatical relations as the true language L but different word order.

We create baseline grammars by randomly sampling the weights for each syntactic relation. These baseline grammars have systematic word-order rules similar to natural language but do not exhibit any correlations among the orderings of different syntactic relations. All four grammars in Fig. 3 are equally likely under this baseline distribution.

For every 1 of the 51 languages, we construct 50 counterfactual baseline versions by randomly creating 50 baseline grammars and applying them to obtain counterfactual orderings for all syntactic structures that were available for that language.

Having defined our space of possible word-order grammars, we now turn to how to define and measure efficiency. Following the information-theoretical literature on language processing, we formalize the communicative efficiency of a language as a weighted combination of two terms: the amount of information that utterances contain about the underlying messages and the cost or difficulty of communication (30, 32–34, 46, 47). We model the informativity term as the degree to which listeners can reconstruct syntactic structures from an utterance, i.e., the parseability of the language. We model the cost or complexity term as the predictability, or negative entropy, of the utterances, since entropy is a standard measure of the complexity of any system of messages (48). We use standard neural-network methods to estimate the numerical values of parseability and predictability from counterfactually ordered corpora. Efficiency is a weighted sum of parseability and predictability. See Materials and Methods for details and *SI Appendix*, section S7 for experiments demonstrating that our results are robust to different methods of estimating parseability and predictability.

For each language, we computationally construct grammars that are optimized for efficiency (Materials and Methods). This optimization problem is challenging because both the parseability and predictability of a sentence can only be evaluated globally, in the context of an entire language. We address this challenge by introducing a simple, differentiable computational formalism for describing grammatical regularities. Our formalism makes it possible to find optimal grammars by standard methods, such as stochastic gradient descent (*SI Appendix*, section S5). For each grammar, we report predictability and parseability as estimated on the data resulting from ordering the syntactic structures from the corpus according to the grammar.

**Fig. 4.**
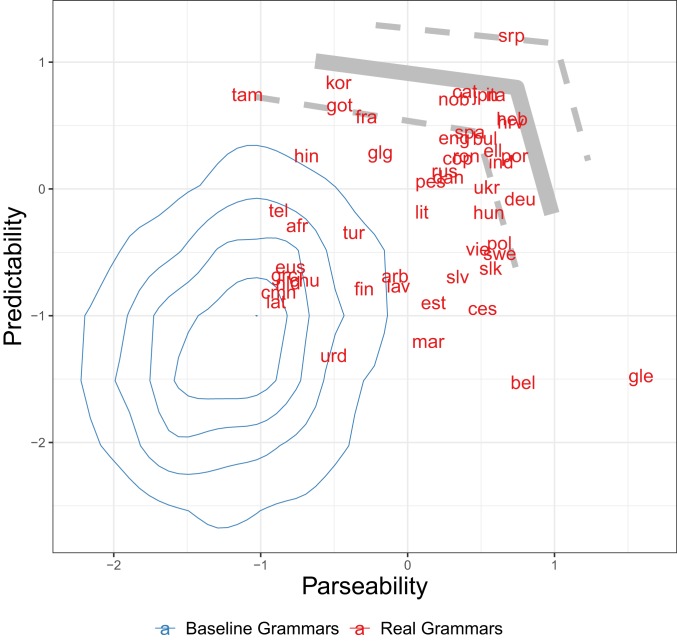
Predictability and parseability of the real word-order grammars of 51 languages (red), indicated by International Organization for Standardization codes, compared to baseline word-order grammars (blue distribution). Predictability and parseability scores are z-scored within language, to enable comparison across languages. The gray curve indicates the approximate Pareto frontier of computationally optimized grammars, averaged over the 51 languages, with dashed SDs.

In Fig. 4, we plot predictability and parseability of the grammars of 51 languages, together with the distribution of random baseline grammars, and the approximate Pareto frontier defined by computationally optimized grammars. This Pareto frontier is approximate because it is an average of the positions of the optimized grammars generated for the corpus of each language. To enable fair comparison with baselines and the estimated frontier, we represent real languages by grammars extracted from the actual orderings observed in the databases. These extracted grammars have the same representational constraints as the baseline and optimized grammars, including the fact that the orders are purely a function of the tree structure and do not take into account other factors, such as discourse structure, which are not annotated in the corpora. For a comparison of the raw word orders from corpora against appropriate baseline grammars, see *SI Appendix*, section S8.

In Fig. 4, we see that real grammars are attracted toward the approximate Pareto frontier and away from the region of the baseline grammars. The majority of real grammars are above and/or to the right of their baseline equivalents, demonstrating that they are relatively high in predictability and/or parseability; 100% of real grammars improve over their baselines on either predictability or parseability (*P* < 0.05, by one-sided t test, with Bonferroni correction and Hochberg step-up procedure); 90% of real grammars improve over the baselines in parseability (*P* < 0.05), and 80% improve in predictability (*P* < 0.05). See *SI Appendix*, section S3 for additional analyses.

## Study 2: Greenberg Word-Order Correlations

We have found that the grammars of human languages concentrate along the Pareto frontier of parseability and predictability. Which grammatical properties characterize Pareto-optimal languages in general, and which properties of human languages make them efficient? Here, we show that all languages close to the Pareto frontier—both real and counterfactual ones—are highly likely to satisfy Greenberg correlation universals. That is, optimizing for efficiency produces languages that satisfy these correlations. In contrast, the baseline grammars are constructed without any correlations between the ordering of different syntactic relations and will therefore mostly not satisfy those universals.

We first considered the 51 real languages. Among the grammars fit to the 51 languages, the number of satisfied correlations is strongly correlated with efficiency (ρ = 0.61, *P* < 0.0001), suggesting that satisfying the correlations improves language efficiency.

We next examine those grammars from Study 1 that we had computationally optimized for efficiency. We controlled for variation across different optima by creating eight optimized grammars for each of the 51 datasets of syntactic structures from real languages. For each real language, we created four optimized grammars with verb–object order and four object–verb grammars. We test whether the process of efficiency optimization produces the Greenberg correlations.

**Fig. 5.**
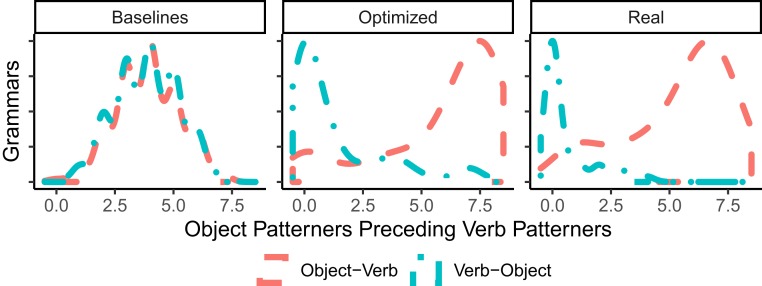
Efficiency optimization produces grammars where the orders of the eight relations in Table 1 are strongly correlated with the order of verb and object. We arrange grammars (baseline, optimized, real) by the number of relations where the language patterns with Japanese (as opposed to with Arabic) and plot a kernel-density estimate. Object–verb order leads to grammars where object patterners precede (like Japanese); verb–object order leads to verb patterners preceding (like Arabic). Baseline grammars show no such correlation.

For each grammar (baseline, optimized, and real), we computed how many of the eight relations in Table 1 had the same order as Japanese (in contrast to Arabic). Fig. 5 shows the results, separately for grammars with verb–object and object–verb orders. In optimized grammars, the order of the eight relations is strongly correlated with the placement of the object, similar to the 51 real languages in our sample. In contrast, baseline languages show no correlation.

We asked whether efficiency optimization predicts the eight correlations to hold in most languages. To answer this question, we constructed a Bayesian multivariate mixed-effects logistic regression model predicting which of the eight correlations an optimized grammar satisfies. We controlled for variation between the syntactic structures used in different languages and language families by entering the language and language family as random effects. See *SI Appendix*, section S4.3 for robustness to modeling choices.

**Fig. 6.**
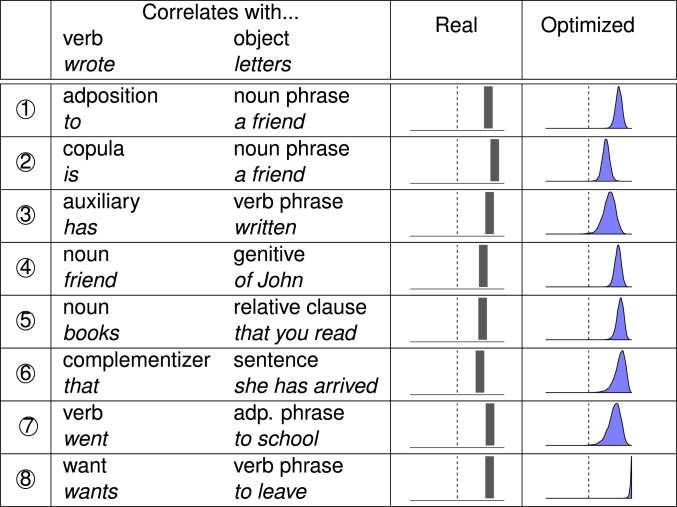
Efficiency optimization accurately predicts the Greenberg correlations. For each correlation, we provide its prevalence (between 0% and 100%) among the actual grammars of the 51 languages (Real), and the posterior distribution of the prevalence among grammars optimized for efficiency (Optimized) on datasets from the 51 languages. Efficiency optimization predicts all eight correlations to hold in the majority of grammars, matching the distribution observed in real languages.

In Fig. 6, we compare the prevalence of the eight correlations in real and optimized languages. For the real languages, we indicate how many of the 51 languages satisfy a correlation. For the optimized languages, we indicate the posterior distribution of the proportion of satisfying languages, obtained from the mixed-effects analysis. Grammars optimized for efficiency predict all eight correlations to hold at prevalences significantly greater than 50%, similar to actual human languages. In the multivariate mixed-effects analysis, efficiency optimization predicts all eight correlations to hold across languages (posterior probability, 0.9911). Optimizing for only predicability or only parseability does not predict all of the correlations (*SI Appendix*, section S4).

## Discussion

We found that the grammars of natural languages are more efficient than baseline grammars and that a large subset of the Greenberg word-order correlations can be explained in terms of optimization of grammars for efficient communication.

Our work makes crucial use of neural-network models for estimating the efficiency of languages. This method currently requires large computational resources; it still takes about 3 wk to create optimized grammars for 51 languages, even with specialized hardware. We believe that further advances in machine learning will reduce the computational cost, making this approach more widely applicable.

What makes the grammars of human languages efficient? Study 2 shows that Greenberg correlations are one key property that real languages share with optimal grammars. Prior work has suggested dependency-length minimization as another characteristic of efficient word order. This is the idea that word order minimizes the average distance between syntactically related words. It is known that human languages reduce this distance compared to random baselines (49–52). Our optimized grammars also share this property: we find that 100% of grammars optimized for efficiency also reduce average distance between related words compared to baselines (*P* < 0.05, by one-sided t test).

To some extent, the Greenberg correlations and dependency-length minimization are related, because the Greenberg correlations help reduce the distance between related words (4, 53). Consider again the sentence “I wrote letters to friends” (cf. Figs. 1 and 3). Both real and optimized grammars of English linearize its syntactic structure as follows:
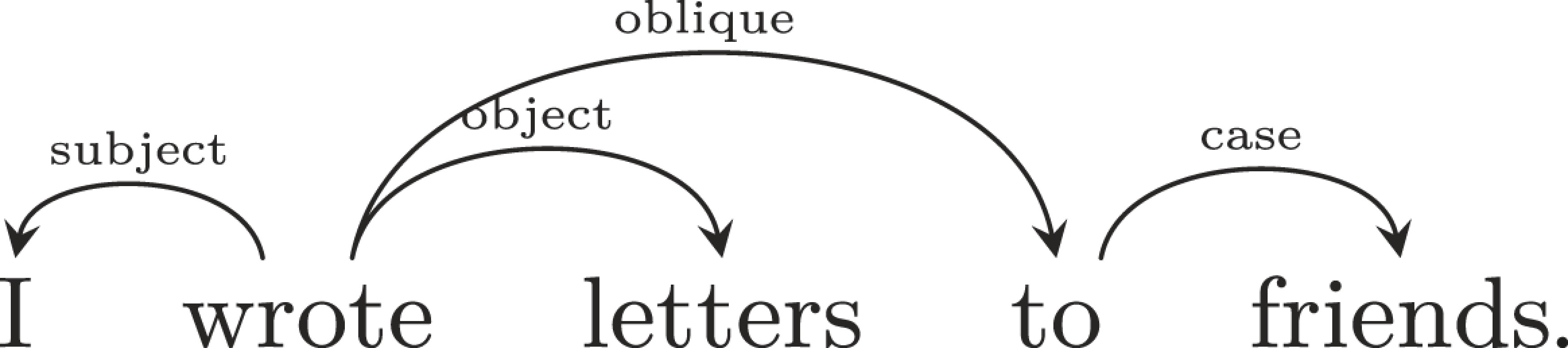


This ordering exhibits correlations 1 and 7 from Table 1. Among all possible ways of ordering this syntactic structure, this one also minimizes the average distance between any two syntactically related words, e.g., inverting “to” and “friends” would increase the distance between “wrote” and “to.”

It may come as a surprise that grammars that are efficient according to our metric also have low dependency length, even though dependency length is never considered explicitly during the calculation of efficiency nor the procedure for optimizing grammars. The result is especially surprising given that our efficiency metric does not incorporate any kind of memory limitations, whereas previous functional explanations for dependency-length minimization have typically been based on the idea of limited working-memory resources available during language production and comprehension (54, 55) (although see ref. 4 for a motivation of dependency-length minimization that is not based in memory limitations). Our results suggest that both Greenberg correlations and dependency-length minimization might be explainable purely in terms of maximizing the general parseability and predictability of utterances, without a need for further constraints. See *SI Appendix*, section S12 for further discussion, along with some simulations demonstrating how grammars that satisfy Greenberg correlations can be more efficient in a generic sense.

An idea related to functional optimization, as we have explored it here, is the idea that grammars are biased toward simplicity in terms of the number of parameters required to specify the grammar (56). For example, it has been proposed that languages have a single head-directionality parameter and that this accounts for the Greenberg correlations (17, 57). As an explanation of correlations, this idea turns out to overpredict correlations (13, 19), and more recent research in syntactic theory has provided evidence against it (58–60). Nevertheless, future research should examine whether there are more principled connections between communicative efficiency and grammar simplicity.

A major question for functional explanations for linguistic universals is: How do languages end up optimized? Do speakers actively seek out new communicative conventions that allow better efficiency? Or do languages change in response to biases that come into play during language acquisition (61, 62)? Our work is neutral toward such questions. To the extent that language universals arise from biases in learning or in the representational capacity of the human brain, our results suggest that those biases tilt toward communicative efficiency.

Unlike cross-linguistic efficiency studies in the domain of lexical semantics (33, 34, 46), we did not derive a single universal bound for the efficiency across all 51 languages in Study 1; instead, we constructed optimized grammars individually for each language. Each language L has its own distribution of tree structures that speakers communicate and different grammars may be optimal for different tree structure distributions (*SI Appendix*, section S3.5). Our results show that the word order of each language L is approximately optimal for the tree structures used in L.

While our work has shown that certain word-order universals can be explained by efficiency in communication, we have made a number of basic assumptions about how language works in constructing our word-order grammars: for example, that sentences can be syntactically analyzed into trees of syntactic relations. We believe a promising avenue for future work is to determine whether these more basic properties themselves might also be explainable in terms of efficient communication.

Our work provides evidence that the grammatical structure of languages is shaped by the need to support efficient communication. Beyond our present results, our contribution is to provide a computational framework in which theories of the efficiency optimization of languages can be tested rigorously. While our study has focused on syntax, our results suggest that this method can be fruitfully applied to testing efficiency explanations in other domains of language structure.

## Materials and Methods

### Corpus Data.

We use the Universal Dependencies (UD) 2.1 data (40). We use all languages for which at least 1 treebank with a training partition was available, a total of 51 languages. For each language where multiple treebanks with training sets were available, we pooled their training sets; similarly for development sets. Punctuation was removed. Universal dependencies represents as dependents some words that are typically classified as heads in syntactic theory. This particularly applies to the “cc,” “case,” “cop,” and “mark” dependencies. Following prior work studying dependency-length minimization (50), we applied automated conversion to a more standard formalism, modifying each treebank by inverting these dependencies and promoting the dependent to the head position. When a head had multiple such dependents, we iteratively applied the conversion until no such dependents were left. Language-specific relation types were truncated to their universal counterparts both in the design of word-order grammars and for modeling parseability.

### Word-Order Grammars.

We adapt the grammar model of ref. 43 to UD. A grammar assigns a parameter $x_{\tau }\in \left[-1,1\right]$ to every relation τ belonging to the 37 universal syntactic relations defined by UD 2.1. A syntactic structure, consisting of a set of words and syntactic relations between them, is then ordered into a string of words recursively starting from the root; the dependents of a word then are ordered around the head according to the values $x_{\tau }$ corresponding to their syntactic relations; those dependents where $x_{\tau }<0$ are ordered before the head; the others are ordered after the head. See *SI Appendix*, section S5.2 for the methodology used to extract the languages’ actual grammars from datasets and for validation against expert judgments.

### Formalizing Efficiency.

We adopt the formalization of language efficiency of ref. 30, closely related to the Information Bottleneck (63), which has recently been successfully applied to model lexical semantics (33). Very similar formalizations of Zipf’s ideas have been proposed across the information-theoretic literature on language (32, 34, 46, 64). See *SI Appendix*, section S2.1 for discussion.

In this framework, the overall efficiency of language is a weighted combination of terms representing the amount of information that utterances contain about the underlying messages and the cost of communication (30, 32–34, 46). We model the first term as the degree to which listeners can reconstruct syntactic structures from an utterance, i.e., the parseability of the language. This is formalized as the amount of information that utterances u provide about their underlying syntactic structures t:

$$R_{Pars}\;≔\;I\left[U,T\right]=\underset{t,u}{\sum }p\left(t,u\right)\log\frac{p\left(t|u\right)}{p\left(t\right)},$$ [1]

where the sum runs over all possible pairs of utterances u and syntactic structures t in the language.

Again following ref. 30, we formalize the complexity of a language as its entropy. This corresponds to the average word-by-word surprisal, the degree to which sentences are unpredictable from the general statistics of the language. Surprisal has been found to be a highly accurate and general predictor of human online processing difficulty (65–67). Entropy is also a general measure of the complexity of any system of messages (48). In expectation over all utterances u in a language, the negative surprisal describes the predictability, or negative entropy, of the utterances:

$$R_{Pred}≔-H\left[U\right]=\underset{u}{\sum }p\left(u\right)\log p\left(u\right),$$ [2]

where the sum runs over all possible sentences u in the language.

Maximizing one of the two scoring functions under a constraint on the other function (e.g., maximizing parseability under a constraint on the minimal predictability) amounts to maximizing a weighted combination of the two scoring functions (30):

$$R_{\mathrm{Eff}}\;≔\;R_{Pars}+\lambda R_{Pred},$$ [3]

with an interpolation weight λ∈[0,1) that controls the relative strength of the two pressures. When optimizing grammars for efficiency, we set λ ≔ 0.9 in Eq. **3** in order to give approximately equal weight to both components. See *SI Appendix*, section S2.2 for mathematical discussion of λ and robustness to other choices.

We estimate predictability using Long Short-Term Memory recurrent neural networks (68), general sequence models that are the strongest known predictors of the surprisal effect on human processing effort (69, 70). We estimate parseability using a generic neural-network architecture that casts recovery of syntactic structures as a minimum spanning-tree problem (71, 72). In order to reduce overfitting in the optimization process, we use an unlexicalized parsing setup and add part-of-speech tags when estimating predictability. Grammars are optimized for efficiency by simultaneous gradient descent on the parameters of the grammar and these neural models. All parseability and predictability values are reported on the held-out (“dev”) partitions from the predefined split for each UD corpus. See *SI Appendix*, sections S5–S8 for details and for robustness of our results to modeling choices, including evidence that our results are not specific to any particular language model or parser.

### Data Availability.

Code and results are available at https://github.com/m-hahn/grammar-optim. The efficiency optimization results from Fig. 6 were preregistered: https://aspredicted.org/th5pk.pdf (see also *SI Appendix*, section S4.6).

## Supplementary Material

Supplementary File
