# Supplementary material for: Universals of word order reflect optimization of grammars for efficient communication
Source: Proc Natl Acad Sci U S A. 2020 Jan 21;117(5):2347–53. doi: 10.1073/pnas.1910923117 (PMC7007543; doi:10.1073/pnas.1910923117)
Supplement: Supplementary File [file pnas.1910923117.sapp.pdf]

Supplemental Materials for "Universals of word order reflect optimization of grammars for efficient communication"

Michael Hahn
Department of Linguistics
Stanford University

Daniel Jurafsky
Department of Linguistics
Stanford University

Richard Futrell
Department of Language Science
University of California, Irvine

November 12, 2019

Contents

S1 Formalization of Greenberg Correlation Universals **2**

S2 Formalizing Communicative Efficiency **4**
S2.1 Derivation and Relation to Other Work . . . 4
S2.2 Choice of λ . . . 6

S3 Supplementary Analyses for Study 1 **7**
S3.1 Details and Additional Analyses . . . 7
S3.2 Analysis controlling for Families . . . 8
S3.3 Quantifying Degree of Optimality for Overall Efficiency . . . 8
S3.4 Parseability and Surprisal Metrics for Observed Orders and Extracted Grammars . . . 13
S3.5 Impact of Tree Structures on Optimality and Estimated Frontier . . . 13

S4 Supplementary Analyses for Study 2 **16**
S4.1 Correlation between Universals and Efficiency . . . 16
S4.2 Predictions for Individual Languages . . . 17
S4.3 Regression for Predicted Correlations . . . 17
S4.4 Comparing Efficiency to its Components . . . 19
S4.5 Results on all UD Relations . . . 19
S4.6 Previous Experiments . . . 20
S4.7 Comparison to other Formalizations of Greenberg's Correlations . . . 20

S5 Creating Optimized Grammars **24**
S5.1 Differentiable Ordering Grammars . . . 24
S5.2 Extracting Grammars from Datasets . . . 26
S5.3 Optimizing Grammars for Efficiency . . . 26

S6 Neural Network Architectures **28**

S7 Robustness to different language models and parsers **31**
S7.1 CKY Parsers . . . 31
S7.2 Distorted graph-based parsers . . . 31
S7.3 n-gram language models . . . 34

S8 Other Methods of Estimating Efficiency and Constructing Baselines in Study 1 **34**
S8.1 Lexicalized Models . . . 34
S8.2 Original UD Format . . . 34
S8.3 Nondeterministic Baseline Grammars . . . 38

S9 Effects of data sparsity **38**

www.pnas.org/cgi/doi/10.1073/pnas.1910923117

S10 Languages and Corpus Sizes 41

S11 Dependency Length Minimization 41

S12 Efficiency and correlating orders in toy grammars 44

S1 Formalization of Greenberg Correlation Universals

Here we describe how we selected the word order correlations in Table 1 of the main paper, and how we formalized these using syntactic relations defined by Universal Dependencies.

We base our formalization on the comprehensive study by Dryer [1].[1] Greenberg's original study was based on 30 languages; more recently, Dryer [1] documented the word order correlations based on typological data from 625 languages. Dryer [1] formulated these universals as correlations between the order of objects and verbs and the orders of other syntactic relations. We test our ordering grammars for these correlations by testing whether the coefficients for these syntactic relations have the same sign as the coefficient of the verb-object relation. Testing correlations is therefore constrained by the degree to which these relations are annotated in UD. The verb–object relation corresponds to the *obj* relation defined by UD. While most of the other relations also correspond to UD relations, some are not annotated reliably. We were able formalize eleven out of Dryer's sixteen correlations in UD. Six of these could not be expressed individually in UD, and were collapsed into three coarse-grained correlations: First, tense/aspect and negative auxiliaries are together represented by the *aux* relation in UD. Second, the relation between complementizers and adverbial subordinators with their complement clauses is represented by the *mark* relation. Third, both the verb-PP relation and the relation between adjectives and their standard of comparison is captured by the *obl* relation.

The resulting operationalization is shown in Table S1. For each relation, we show the direction of the UD syntactic relation: $\rightarrow$ indicates that the verb patterner is the head; $\leftarrow$ indicates that the object patterner is the head.

As described in *Materials and Methods*, we follow Futrell et al. [5] in converting the Universal Dependencies format to a format closer to standard syntactic theory, promoting adpositions, copulas, and complementizers to heads. As a consequence, the direction of the relations *case*, *cop*, and *mark* is reversed compared to Universal Dependencies. For clarity, we refer to these reversed relations as *lifted_case*, *lifted_cop*, and *lifted_mark*.

	Correlates with...		UD Relation	Greenberg [6]
	verb	object		
①	adposition	NP	$\xrightarrow{lifted\_case}$	3, 4
②	copula verb	predicate	$\xrightarrow{lifted\_cop}$	–
③	tense/aspect auxiliary<br>negative auxiliary	VP<br>VP	$\xleftarrow{aux}$	16, 13<br>–
④	noun	genitive	$\xrightarrow{nmod}$	2, 23
⑤	noun	relative clause	$\xrightarrow{acl}$	24
⑥	complementizer<br>adverbial subordinator	S<br>S	$\xrightarrow{lifted\_mark}$	–<br>–
⑦	adjective<br>verb	std. of comp.<br>PP	$\xrightarrow{obl}$	–<br>22
⑧	'want'	VP	$\xrightarrow{xcomp}$	15

Table S1: Greenbergian Correlations based on Dryer [1], with operationalizations with Universal Dependencies using the modified format of [5] (see text). For reference, we also provide the numbers of the closest corresponding universals stated in Greenberg's original study, to the extent that this is possible.

Excluded Correlations Here, we discuss in more detail the five correlations from Dryer's study that we had to exclude. First, we excluded three correlations that are not annotated reliably in UD, and are only relevant to some of the world's languages: Question particles, plural words (i.e., independent plural markers), and articles. All three types of elements occur at most in parts of the 51 UD languages, and none of them is annotated reliably in those languages where they occur. Among these three types of elements, the one most prominent in our sample of 51 languages is articles, which occur in many European languages. However, UD subsumes them under the *det* relation, which is also used for other highly

[1] Regarding the objections by Dunn et al. [2], we refer to the follow-ups by Levy and Daumé [3], and Croft et al. [4].

frequent elements, such as demonstratives and quantifiers. The other elements (question particles and plural words) are found at most in a handful of UD languages, and are not specifically annotated in these either.

We also excluded the verb-manner adverb correlation. UD does not distinguish manner adverbs from other elements labeled as adverbs, such as sentence-level adverbs and negation markers, whose ordering is very different from manner adverbs. All types of adverbs are unified under the *advmod* relation. In the real orderings in our sample of 51 UD languages, the dominant ordering of *advmod* almost always matches that of subjects – that is, *advmod* dependents are predominantly ordered after the verb only in VSO languages. This observed ordering behavior in the 51 languages is very different from that documented for manner adverbs by Dryer, showing that a large part of *advmod* dependencies as annotated in UD consists of elements that are not manner adverbs.

We further excluded the verb-subject correlation, which is not satisfied by much more than half of the world's languages (51 % among those with annotation in the *World Atlas of Language Structures* [7], with clear violation in 35.4 %). It is satisfied only in 33% of our sample of 51 UD languages, as quantified using the grammars we extracted. Dryer [1] counts this as a correlation since he describes the distribution of subject order as an interaction between a weak correlation with object order, and a very strong dominance principle favoring SV orderings. We focus on the modeling of correlations, and leave dominance principles to future research. We therefore excluded this correlation here.

Other Greenberg Universals Greenberg [6] stated a total of 45 universals. Twenty of these concern the structure of individual words (as opposed to word order, which we focus on here), and many of those have been argued to be explained by the "dual pressures" idea [8]. The other 25 universals concern word order; Dryer [1] reformulated most of these as correlations with verb-object order; these form the basis of our formalization in Table S1. There are a few other well-supported word order universals that are not correlations with verb-object order. This includes dominance principles [6, 9] such as the strong preference for subjects to precede objects. Furthermore, there has been interest in Greenberg's universals 18 and 20, which describe correlations not with verb-object order, but of different elements of noun phrases [10, 11, 12]. Future work should examine whether these universals can also be linked to efficiency optimization.

Evaluating Accuracy of Formalization An anonymous reviewer notes that the mapping between Dryer's relations and UD is not perfect, since some of the UD relations subsume other relations. Here we provide evidence that this is not impact our conclusions, since the ordering of the various relations subsumed under the UD label strongly agree typologically.

1. Correlation ③ captures correlations with inflected tense, aspect, and negation auxiliaries as stated by Dryer [1]; however, *aux* aso encompasses other types of auxiliaires, such as modals. We note that other authors, including Greenberg [6], have stated the correlation for all inflected auxiliaries; for further references, we refer to Plank and Filimonova [13, Number 501].

   We used the UD treebanks to confirm that different auxiliaries tend to pattern together, and that the most frequent order of the *aux* relation coincides with that of inflected tense-aspect or negation auxiliaries.

   We collected, for each UD language, all dependents of the *aux* dependency, occurring at least 10 times, and compared their dominant orders, which we operationalized as their more common order in the treebank (auxiliary–head or head–auxiliary). The dependency occurs in all but two very small treebanks (Telugu and Irish). In 43 languages, all extracted auxiliaries had the same dominant order, with the possible exception of uninflected particles labeled *aux* (Croatian, German, Polish, Ukrainian). In three languages (Ancient Greek, Russian), there were other auxiliaries with different dominant order, but these were modal or passive auxiliaries. Finally, in three languages (Afrikaans, Old Church Slavonic, and Persian), not all tense-aspect auxiliaries showed the same dominant order as the *aux* dependency overall. For instance, in Persian, the perfect auxiliary *budan* follows the main verb, whereas the future auxiliary *xaastan xaah-* precedes it [14, pp. 117, 121].

   Taken together, this shows that the dominant order of the *aux* relation strongly coincides with that of inflected tense-aspect auxiliaries, except for a small number of languages where different tense-aspect auxiliaries show different orders.

2. Correlation ④ is formalized using *nmod* which covers not only genitives, but also all other noun-modifying NPs and PPs. The evaluation of extracted grammars against WALS (Table S11) shows that, among the 37 languages where WALS has an entry, the dominant direction of *nmod* agrees with that of genitives, with two exceptions (Danish and Swedish[2].

[2]Danish and Swedish have genitives preceding the head marked with *-s* similar to English (reflected in the WALS entry), while noun-modifying PPs, including phrases similar to English *of* phrases, follow the head. In these two languages, the order of adnominal PPs, agreeing with the more frequent order of *nmod* relations, agrees with the verb-object relation, whereas prenominal -s genitives show the opposite ordering.

3. Correlation ⑤ is formalized using *acl*, which covers not just relative clauses, but also other adnominal clauses. In the WALS evaluation (Table S11), the dominant order of *acl* agrees with the WALS entry for relative clauses in all but three languages (Estonian, Finnish, Tamil) out of the 36 languages for which WALS has an entry. Also, UD provides a specific *acl:relcl* sub-label for relative clauses in 21 of the languages. In all but three languages, the dominant order is the same for the general *acl* label as for the specific *acl:relcl* one (exceptions: Estonian, Finnish, Hindi).

   The exceptions mainly arise because some languages have multiple common word orders for relativization: Hindi uses correlatives that can precede or follow the coreferent noun [15, 3.1.3] and relatives following the noun [15, 4.3]. Estonian and Finnish have finite relative clauses following nouns ([16, p. 176], [17, p. 256]) and nonfinite participial modifiers preceding it [16, Chapter 18].

   Finally, in Tamil, the divergence is caused by the treebank annotation convention for Tamil, where the label *acl* is used to mark certain elements of compounds, not for the participial phrases that correspond most closely to relative clauses of other languages.[3]

4. Correlation ⑦ is formalized using *obl*, which covers not only PPs and standards of comparison, but also adjunct NPs. In the WALS evaluation (Table S11), the dominant order of *obl* agrees with that annotated for obliques in all 18 languages for which WALS has an entry.

5. Correlation ⑧ is formalized using *xcomp*, which covers other control verbs, not just verbs of volition ('want').

   We used the UD treebanks to investigate whether there are differences in the ordering of 'want' and other verbs using the *xcomp* dependency.

   The dependency is annotated in all but two languages (Japanese and Turkish).

   For each language, we extracted all lemmas of words heading an *xcomp* dependency, occurring at least 10 times. In 39 languages, all extracted words had the same dominant order. Additionally, in four Germanic languages (Afrikaans, Danish, Dutch, and German), the verb of volition (Afrikaans *wil*, Danish *ville*, Dutch *willen*, German *wollen*) is mostly annotated with the *aux* relation due to UD annotation guidelines, but in all languages, its dominant order (verb of volition before its complement) agrees with the dominant order of the *xcomp* dependency (head-initial). In three historical languages (Ancient Greek, Latin, and Old Church Slavonic), verbs of volition agree with the dominant order of *xcomp*, while several other verbs that do not indicate volition show opposite dominant order. Finally, in Gothic, the verb of volition (*wiljan*) has opposite dominant order, resulting in an apparent violation of Correlation ⑧.

   Taken together, the order of 'want' and its complement tends to agree with that of most other *xcomp* dependencies, with the sole exception of Gothic.

S2 Formalizing Communicative Efficiency

S2.1 Derivation and Relation to Other Work

Here we discuss how our formalization of communicative efficiency relates to formalizations that have been proposed in the information-theoretic literature on language. Across the literature, the core idea is to maximize the **amount of information** that linguistic forms provide about meanings, while constraining **complexity and diversity** of forms:

$$\text{Informativity} - \lambda \cdot \text{Complexity}, \tag{1}$$

with some differences in the precise formalization of these two quantities [20, 21, 22, 23, 24, 25, 26, 27, 28, 29, 30, 31, 32, 33, 34, 35].

Derivation of our Formalization The basis for our precise formalization is the function proposed in [20, 21, 30, 34] as a general efficiency metric for communicative systems. If S denotes signals (e.g., words, sentences) and R denotes their referents (e.g., objects in a reference game), then this efficiency metric takes the form (notation slightly varies across these publications):

$$\mathrm{I}[S, R] - \lambda \cdot \mathrm{H}[S], \tag{2}$$

[3] In the original HamleDT [18, 19] version of the Tamil treebank, these relations were labeled as CC, marking compounds (`http://ufal.mff.cuni.cz/~ramasamy/tamiltb/0.1/dependency_annotation.html`). We did not attempt to modify this labeling convention.

where $\mathrm{I}[S, R]$ describes the **informativity** of the signals S about their referents R, and $\mathrm{H}[S]$ describes the **complexity** of the communication system, and $\lambda \geq 0$ trades off the two aspects of efficiency. While prior studies [20, 22, 28, 31] mostly considered settings where the signals S are individual words without further structure, the signals are entire sentences $\mathcal{U}$ in our setting. The underlying messages R which the speaker aims to convey are the syntactic structures $\mathcal{T}$. By the principle of compositionality [36], the meaning of a sentence is a function of the meanings of the parts and how they are combined. The syntactic structure specifies how the meanings of words are combined; therefore, recovering the syntactic structure is a prerequisite to understanding a sentence correctly. Hence, substituting utterances $\mathcal{U}$ for signals S, and syntactic structures $\mathcal{T}$ for underlying messages R, into (2), we arrive at the following efficiency metric for word order:

$$R_{Eff} := R_{Pars} + \lambda \cdot R_{Pred}, \tag{3}$$

where **parseability** is the amount of information that utterances provide about their underlying syntactic structures:

$$R_{Pars} := \mathrm{I}[\mathcal{U}, \mathcal{T}] = \sum_{t,u} p(t, u) \log \frac{p(t|u)}{p(t)}, \tag{4}$$

and **predictability** is the negative entropy or surprisal of the language:

$$R_{Pred} := -\mathrm{H}[\mathcal{U}] = \sum_{u} p(u) \log p(u). \tag{5}$$

Parseability $\mathrm{I}[\mathcal{U}, \mathcal{T}]$ is higher if utterances provide more information about their underlying syntactic structure. Due to the identity $\mathrm{I}[\mathcal{U}, \mathcal{T}] = \mathrm{H}[\mathcal{T}] - \mathrm{H}[\mathcal{T}|\mathcal{U}]$, parseability is maximized if every utterance can be parsed unambiguously—that is, if the listener's uncertainty about syntactic structures given received utterances, $\mathrm{H}[\mathcal{T}|\mathcal{U}]$, is zero. Predictability $-\mathrm{H}[\mathcal{U}]$ is higher if the distribution over utterances is concentrated on a few utterances, and is maximized if there is just a single utterance. It is also equal to the negative average of surprisal, which is a strong and linear predictor of human language processing effort [37, 38, 39].

Relation to Models of Semantic Typology Our model of language efficiency is closely related to models of semantic typology that quantify the efficiency of mappings between concepts and individual words, applied with great success across different domains such color words, container names, and kinship terms [22, 26, 28, 29, 31, 35]. We discuss how our metric (2-3) relates to metrics assumed in this literature, and describe why (2-3) is most appropriate to our setting.

This efficiency metric (2-3) is part of the Information Bottleneck family of models. The Information Bottleneck was introduced by Tishby et al. [40] and has recently been applied to modeling word meaning across different domains by Zaslavsky et al. [31] and Zaslavsky et al. [35]. In the standard Information Bottleneck, complexity is modeled using a mutual information term, instead of the entropy term appearing in (2). The setting for the standard Information Bottleneck is a case where there is a random variable X which contains information about some underlying variable of interest Y; the goal of the Information Bottleneck is to find a representation $\hat{X}$ of X which maximizes $\mathrm{I}[\hat{X}, Y]$ while minimizing $\mathrm{I}[\hat{X}, X]$. One key property of the standard Information Bottleneck is that it results in codes $\hat{X}$ that are nondeterministic.

The variant of the Information Bottleneck that we use has been explored in the machine learning literature by Strouse and Schwab [41] and dubbed the "Deterministic Information Bottleneck" because, in the setting studied by Strouse and Schwab [41], it results in codes that are a deterministic function of the information to be expressed. We use this version of the Information Bottleneck because (1) it has been proposed in previous literature as a generic formalization of efficiency [20], and (2) it is not clear what would count as the three variables Y, X, and $\hat{X}$ in our setting. In our setting we have unordered tree structures $\mathcal{T}$ to be conveyed, and utterances $\mathcal{U}$ representing them. It is not currently clear what would count as a third variable for the application of the standard Information Bottleneck, although we believe such formulations may be fruitful in the future.

A few other approaches to formalizing efficiency share the mutual information term for informativity in (2), while using complexity measures that are not explicitly information-theoretic. In studies of semantic typology by Regier et al. [42], Xu and Regier [26], Xu et al. [29], the complexity function is the number of different forms. As the entropy of a finite and uniform distribution is the logarithm of the number of objects, this complexity function arises from the entropy measure $\mathrm{H}[S]$ (2) in the special case where all forms are used at equal frequency. Notably, the models of Regier et al. [42] and Xu et al. [29] have since been reformulated successfully in the Information Bottleneck formalism [31, 35], bringing them even closer to our formalization of efficiency.

Relation to Models of Language Evolution Our model is also related to models of language evolution. Most closely related to our work, Kirby et al. [27] model language evolution as balancing the pressure towards simple languages with

the pressure for languages to be informative about the intended meanings. Formally, their model studies a Bayesian language learner who infers a language h from data d according to $P(h|d) \propto P(d|h)P(h)$, where $P(h)$ defines a prior distribution over languages, and $P(d|h)$ is the likelihood of observed data d under the grammar h, assuming that speakers produce utterances pragmatically. The prior $P(h)$ favors less complex languages; the likelihood $P(d|h)$ favors languages that communicate meanings unambiguously. We now show that this model instantiates the basic objective (1). If the dataset d consists of observed pairs (t, f) of meanings t and forms f, and the language h defines a set of possible pairs (t, f), then the log-likelihood as defined by their model can be written as follows (up to constants):[4]

$$\begin{aligned}
\log P(d|h) &= \sum_{(t,f)\in d} \log P(f|h,t) \\
&= \sum_{(t,f)\in d} \log P(f|h,t) \\
&\propto \sum_{(t,f)\in d} \log \frac{1}{|\{t' : (t',f) \in h\}|} \\
&= \sum_{(t,f)\in d} \log P(t|f),
\end{aligned}$$

where $P(t|f)$ is the probability that the observed form f referred to meaning t, as the model assumes uniform meaning distributions and uniform choice of appropriate forms. Replacing the sum over the dataset d by the expectation over the idealized full distribution over meaning-form pairs, this can be rewritten as

$$-\mathrm{H}[t|f] = \mathrm{I}[t,f] - \mathrm{H}[t], \tag{6}$$

where the first term is the mutual information between forms and meanings, as in our efficiency metric (2-3). The second term, the entropy of meanings, is a constant independent of the form–meaning mapping. The overall log probability assigned by the Bayesian learner thus comes out to (up to constants)

$$\log P(h|d) = \mathrm{I}[t,f] + \lambda \log P(h), \tag{7}$$

where the prior $P(h)$ favors simpler languages. This result shows that the model of Kirby et al. [27] predicts that language evolution favors languages optimizing a function of the form (1), with an informativity term identical to that of our model (2-3).

Relation to Formalizations of Pragmatics In addition to these models, which concern the efficiency and evolution of communication systems, there is closely related work formalizing the optimal choice of specific utterances in context. Our work is most closely related to the Rational Speech Acts model of pragmatic reasoning [23, 24, 25]. In line with the other models discussed here, it assumes that rational speakers choose utterances to optimize informativity about the referent object, and trade this off with the cost of the utterance, which is partly chosen to be the surprisal of the utterance [32, 33, 34]. Peloquin et al. [34] provide further discussion of the links between pragmatics and the efficiency metric (2-3).

Relation to Models in Other Disciplines Beyond the study of natural language, the efficiency metric (2) is also closely related to information-theoretic models in other disciplines. The tradeoff between informativity and complexity of communication systems is studied extensively in rate–distortion theory [43]. Our efficiency metric is closely related to the the *Infomax principle* from theoretical neuroscience, which is a theory of how information is encoded in neuronal signals. The Infomax principle derives parsimonious data representations by maximizing the mutual information between data and representations, subject to constraints on the representations [44]; a constraint on the representation entropy leads to a metric equivalent to (2) and to a version of the Free-Energy principle (see Section S3 in Friston [45]). A family of Infomax models called "Coherent Infomax" has been proposed by Kay and Phillips [46]; our efficiency metric is a special case within this framework.

S2.2 Choice of λ

In the efficiency objective (3)

$$R_{Eff} := R_{Pars} + \lambda R_{Pred}, \tag{8}$$

[4]We assume for simplicity that the error probability ϵ in the model is equal to 0.

the value of λ is constrained to be in $[0, 1)$. This means, surprisal must be weighted less strongly than parseability.

The reason is that greater values of λ can mathematically result in degenerate solutions. To show this, note that the following inequality always holds:

$$\mathrm{I}[\mathcal{U}; \mathcal{T}] \leq \mathrm{H}[\mathcal{U}]. \tag{9}$$

Therefore, if $\lambda \geq 1$, the efficiency objective satisfies $R_{Eff} = \mathrm{I}[\mathcal{U}; \mathcal{T}] - \lambda \mathrm{H}[\mathcal{U}] \leq 0$, and it takes the maximal possible value of zero if there is only a single utterance $\mathcal{U}$, in which case both $\mathrm{I}[\mathcal{U}; \mathcal{T}]$ and $\mathrm{H}[\mathcal{U}]$ are zero. This is a degenerate language with only a single utterance, which is simultaneously used to convey all meanings. While the design of our word order grammars (see *Materials and Methods*) precludes a collapse of all syntactic structures to a single utterance, this shows that an objective with $\lambda \geq 1$ cannot be a generally applicable description of the efficiency of communication systems. In conclusion, λ is constrained to be in $[0, 1)$, with values closer to 1 placing similar weights on both predictability and parseability, whereas values closer to 0 diminish the role of predictability.

In our experiments, we chose $\lambda = 0.9$ as a mathematically valid value that puts similar weight on both predictability and parseability. While the computational cost of grammar optimization precluded repeating the experiment for many values of λ, we also examined word order predictions for grammars optimized for only parseability or only predictability, in order to tease apart predictions made by these two components. As shown in Table S7, each of the eight correlations is predicted by at least parseability or predictability, without any contradictory predictions. That is, at λ close to its maximal value, the predictions of optimizing the two scoring functions individually add up to the predictions of efficiency optimization.[5] Small values of λ correspond to the case where predictability plays no role, and only parseability is optimized (Table S7), in which case not all correlations are predicted (Figure S8). This is confirmed by converging evidence from our preregistered preliminary experiments in Figure S9.

S3 Supplementary Analyses for Study 1

S3.1 Details and Additional Analyses

In Figure S1, we show the predictability-parseability planes for every one the 51 languages, together with Pareto frontiers estimated from optimized grammars. Figure 4 in the main paper shows the average of these per-language plots, with a kernel density estimate of the distribution of baseline grammars. In addition to the z-scored values in Figure S1 and the main paper, we also provide the raw numerical values, before z-scoring, in Figure S2.

Note that, in a few languages, the real grammar is at a position slightly *beyond* the estimated Pareto frontier. This can be attributed to two reasons: First, stochastic gradient descent introduces noise due to its stochasticity and will only approximately find an optimal solution; second, for some corpora, there may be some degree of distributional mismatch between the training partitions (on which grammars are optimized) and held-out partitions (on which efficiency is estimated). This in particular applies to very small corpora such as Irish (121 training sentences).

Method applied for z-transforming and for estimating Pareto frontier We z-transformed on the level of individual languages, normalizing the mean and SD parseability and predictability of the (1) real grammar, (2) the mean of predictability and parseability of all random grammars, (3) the grammar optimized for efficiency (at $\lambda = 0.9$, see Section S2.2), (4) grammar optimized for parseability only, and (5) grammar optimized for predictability only. For (3-5), we choose the grammar, among all eight optimized grammars, that has the highest estimated efficiency (paresability, predictability) value.

We define the *Pareto frontier* as the boundary of the *set of Pareto-efficient points*, that is, of those points such that no grammar (expressible in our formalism) has both higher predictability and higher parseability. We approximately estimate this frontier based on optimized grammars, by constructing a *lower bound* on this curve from the optimized grammars: Among the eight grammars optimized for efficiency (at $\lambda = 0.9$), we select the one with the highest estimated efficiency value; similarly for grammars optimized for parseability and predictability. Connecting these three grammars results in a piecewise linear curve that is guaranteed to be a lower bound on the true Pareto frontier (meaning that the true Pareto frontier can only lie above to the right of this curve). In cases where the grammar optimized for predictability (similarly parseability) has lower predictability (and parseability) than the grammar optimized for efficiency, we can replace its predictability value by that of the grammar optimized for efficiency: This is guaranteed to result in a point that is still Pareto-dominated by the grammar optimized for efficiency, and provides a tighter bound on the true curve. The resulting frontier is guaranteed to provide a lower bound on the true Pareto frontier, but is nonetheless approximate: the actual

[5] Results from one of the preliminary experiments reported in Figure S9 show that results are stable to small variation of λ: Essentially equivalent predictions are obtained for $\lambda = 1.0$. While $\lambda = 1.0$ is not a valid choice for communicative efficiency in general due to the possibility of collapse to a single utterance, our setting does not allow such a collapse, as the syntactic structure already determines which words are present in the sentence.

curve may not be piecewise linear, and it may also extend beyond the estimated curve, as the grammar optimization method is approximate.

Further Analysis of Optimality In the main paper, we tested whether real grammars are more efficient than the mean of baseline grammars, using a t-test. We also conducted the analysis using a Binomial test (one-sided), testing whether the real gramar is more efficient than the *median* of baseline grammars, avoiding any distributional assumption on the baseline grammars. As before, we used Hochberg's step-up procedure (Note that the tests for different languages are independent, as different baseline grammars are evaluated for each language), with $\alpha = 0.05$. In this analysis, real grammars improved in parseability for 80% of languages, in predictability for 69% of languages, and in either of both in 92% of languages ($p < 0.05$, with Bonferroni correction). In Table S2, we provide per-language results for the t-tests and binomial tests.

S3.2 Analysis controlling for Families

The UD treebanks overrepresent certain language families. This raises the question of whether the relative optimality of real grammars observed in Study 1 could be due to family-specific effects. We address this question in this section, by estimating the overall degree of optimization of languages for efficiency, controlling for differences between families. To this end, we constructed a Bayesian logistic mixed-effects model estimating, for each language L among the 51 UD languages, the rate $q_L \in [0, 1]$ of random baseline grammars that have *higher* efficiency (parseability, predictability) than the real grammar. We entered languages and language families as random effects:

$$logit(q_L) = \beta + u_L + v_{f_L} \tag{10}$$

where f_L is the language family of L. Here, β models the overall probability $logit(q_L)$ of a baseline grammar having higher efficiency than the real grammar, controlling for differences in the tree structures and real grammars of different languages and language families. If optimality of real grammars holds generally across families, and exceptions are due to to language- or family-specific effects, we expect β to be < 0 significantly. On the other hand, if optimality of real grammars does not generally hold across families, and the observed optimality is due to family-specific effects, then we expect $\beta \geq 0$.

We estimated the mixed-effects model (10) from the 50 baseline grammars for each language, using the same priors and sampling method as in the analysis in Study 2 (reported in Section S4.3).

Results for the posterior of β are shown in Table S3. For all three models, β is estimated to be < 0, showing that the observed optimality of real grammars holds across families, and exceptions are due to language- or family-specific effects. For instance, for efficiency, the posterior mean estimate $\beta = -5.88$ corresponds to less than 1% of baseline grammars showing higher efficiency than the real grammar, when controlling for language- and family-specific effects. Similar results hold for predictability and parseability individually.

S3.3 Quantifying Degree of Optimality for Overall Efficiency

In the main paper (Study 1), we showed that languages tend to be optimized for parseability and/or predictability. Efficiency is a combination of both components; in this section we address the question whether languages are generally optimized for efficiency as a multi-objective optimization problem of optimizing for parseability and predictability.

Recall the efficiency metric

$$R_\lambda := R_{Pars} + \lambda R_{Pred} \tag{11}$$

with the tradeoff parameter $\lambda \in [0, 1)$. For each possible value of $\lambda \in [0, 1)$ trading off parseability and predictability, we quantify what fraction of the baseline grammars are less efficient than the real language.

The results are plotted in Figure S3. For all languages, there are some values of λ where the real grammar improves at least half of the baseline grammars. In about 40 of the languages, the real grammar improves over almost all baseline grammars and for all values of λ. This shows that, while many languages do not improve over *all* baselines on *both* individual components, they mostly improve over the large majority of baselines on the combined objective of efficiency, even across different values of λ. For instance, the real grammar of Czech does not improve over all baselines in predictability (see Figure S1), but it has higher overall efficiency than the vast majority of baselines in efficiency, for all values $\lambda \in [0, 1)$. There are also languages for which the degree of optimality does strongly depend on λ; however, we note that estimated optimimality is stronger when estimating efficiency using lexicalized parsers that can take morphology into account (Figures S14-S15).

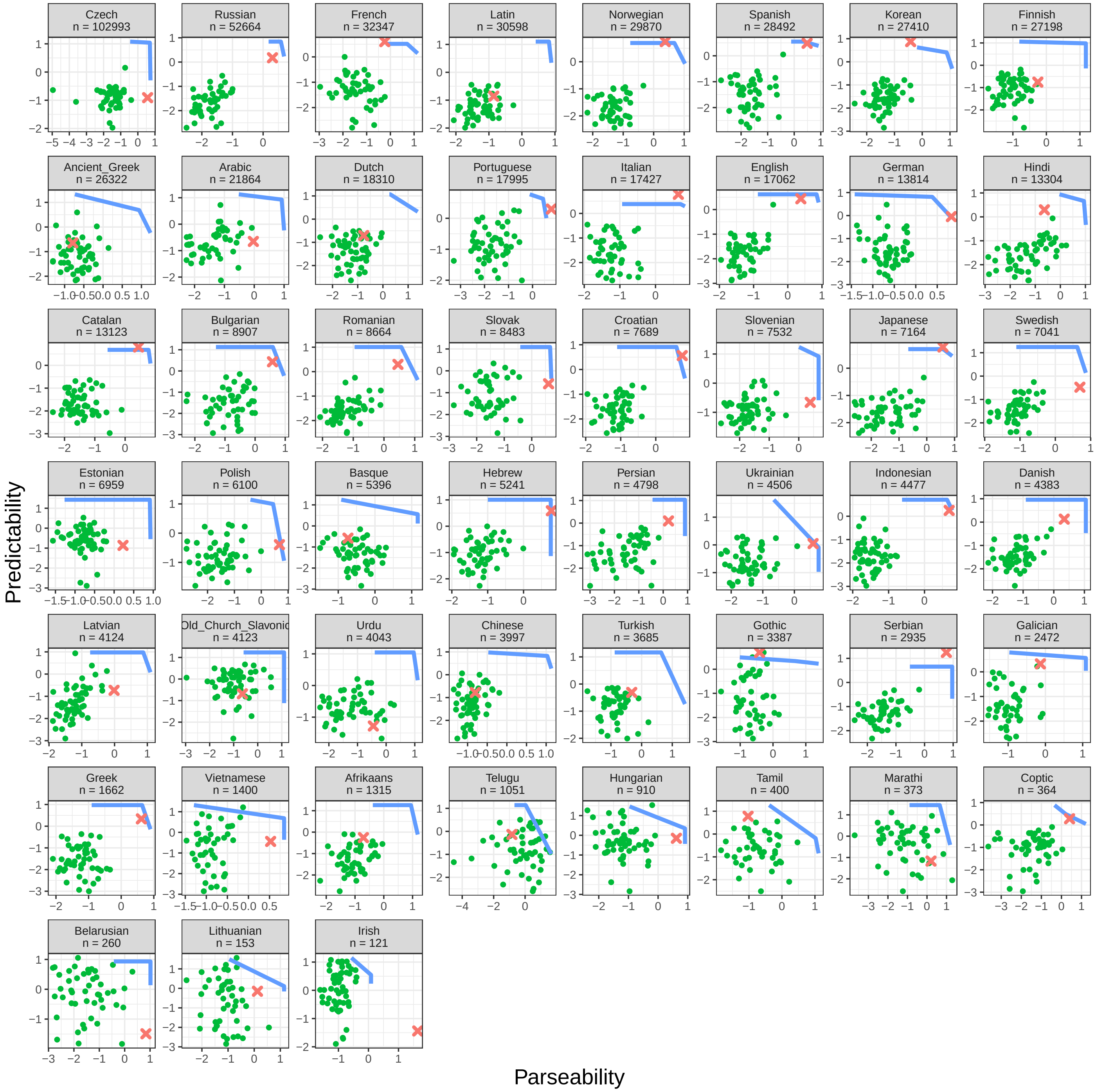

Figure S1: Predictability and parseability of 51 languages, ordered by corpus size, measured by the number of sentences in the training partition, from largest (Czech) to smallest (Irish). Green: random baselines, Red: real grammar, blue: approximate Pareto frontier, computed from the optimized grammars. All data are z-scored.

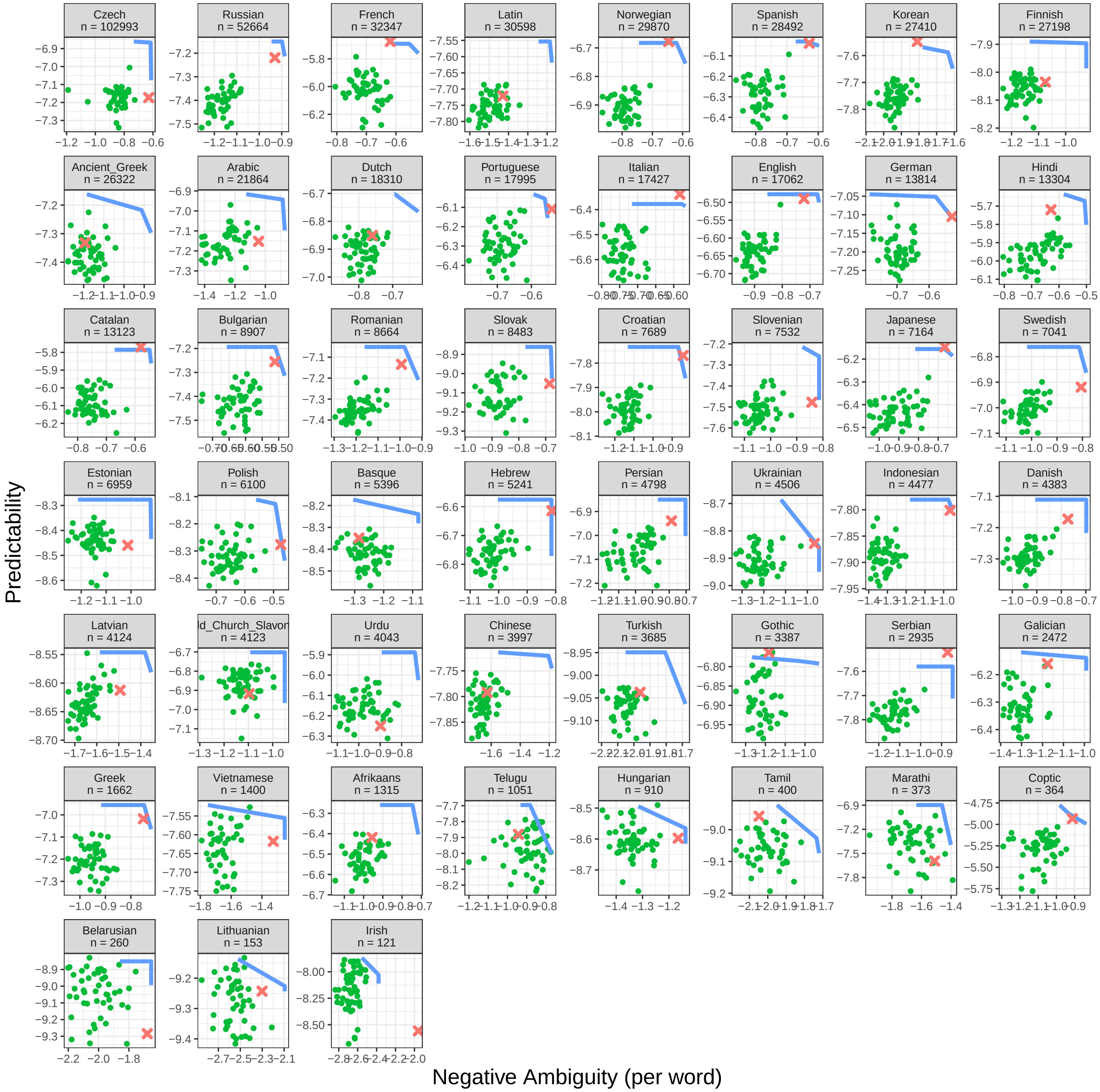

Figure S2: Raw numerical values estimated for Predictability (negative surprisal), and negative syntactic ambiguity $-H[T|U]$, before z-scoring. For more meaningful comparison, both quantities are normalized by the number of words in the corpus, i.e., we plot per-word negative surprisal and per-word difficulty in recovering syntactic structures. Note that the negative syntactic ambiguity $-H[T|U]$ equals parseability $I[T,U] = H[T] - H[T|U]$ up to a per-language constant $H[T]$, which we do not attempt to estimate. Further note that we use different scales in the different panels.

Language	Pred. (t)	Parse. (t)	Pred. (Binomial)			Parseab. (Binomial)		
	p	p	Est.	CI	p	Est.	CI	p
Afrikaans	5.29×10^{-13}	1.46×10^{-6}	0.96	$[0.89, 1]$	1.59×10^{-13}	0.8	$[0.69, 1]$	7.01×10^{-6}
Ancient Greek	1.17×10^{-7}	0.998	0.8	$[0.69, 1]$	7.01×10^{-6}	0.33	$[0.22, 1]$	0.997
Arabic	0.0774	$<2 \times 10^{-16}$	0.57	$[0.44, 1]$	0.196	0.98	$[0.92, 1]$	1.55×10^{-15}
Basque	2.69×10^{-13}	1	0.89	$[0.79, 1]$	2.9×10^{-9}	0.31	$[0.21, 1]$	0.999
Belarusian	1	$<2 \times 10^{-16}$	0.14	$[0.07, 1]$	1	1	$[0.95, 1]$	$<2 \times 10^{-16}$
Bulgarian	$<2 \times 10^{-16}$	$<2 \times 10^{-16}$	1	$[0.94, 1]$	8.88×10^{-16}	1	$[0.95, 1]$	$<2 \times 10^{-16}$
Catalan	$<2 \times 10^{-16}$	$<2 \times 10^{-16}$	1	$[0.95, 1]$	$<2 \times 10^{-16}$	1	$[0.95, 1]$	$<2 \times 10^{-16}$
Chinese	1.56×10^{-6}	0.0115	0.75	$[0.64, 1]$	0.000 117	0.7	$[0.58, 1]$	0.002 28
Coptic	0.001 75	$<2 \times 10^{-16}$	1	$[0.94, 1]$	1.78×10^{-15}	1	$[0.95, 1]$	$<2 \times 10^{-16}$
Croatian	$<2 \times 10^{-16}$	$<2 \times 10^{-16}$	1	$[0.94, 1]$	4.44×10^{-16}	1	$[0.95, 1]$	$<2 \times 10^{-16}$
Czech	0.438	$<2 \times 10^{-16}$	0.46	$[0.34, 1]$	0.756	1	$[0.94, 1]$	2.84×10^{-14}
Danish	$<2 \times 10^{-16}$	$<2 \times 10^{-16}$	1	$[0.95, 1]$	$<2 \times 10^{-16}$	1	$[0.95, 1]$	$<2 \times 10^{-16}$
Dutch	1.41×10^{-11}	2.33×10^{-7}	0.87	$[0.77, 1]$	6.54×10^{-9}	0.76	$[0.65, 1]$	5.68×10^{-5}
English	$<2 \times 10^{-16}$	$<2 \times 10^{-16}$	1	$[0.94, 1]$	1.78×10^{-15}	1	$[0.95, 1]$	$<2 \times 10^{-16}$
Estonian	0.942	$<2 \times 10^{-16}$	0.27	$[0.18, 1]$	1	1	$[0.95, 1]$	$<2 \times 10^{-16}$
Finnish	8.85×10^{-6}	$<2 \times 10^{-16}$	0.7	$[0.58, 1]$	0.002 74	1	$[0.95, 1]$	$<2 \times 10^{-16}$
French	4.22×10^{-9}	$<2 \times 10^{-16}$	1	$[0.94, 1]$	8.88×10^{-16}	0.98	$[0.91, 1]$	6×10^{-15}
Galician	8.48×10^{-15}	$<2 \times 10^{-16}$	1	$[0.94, 1]$	1.78×10^{-15}	0.95	$[0.87, 1]$	4.07×10^{-13}
German	$<2 \times 10^{-16}$	$<2 \times 10^{-16}$	0.98	$[0.91, 1]$	1.18×10^{-14}	1	$[0.95, 1]$	$<2 \times 10^{-16}$
Gothic	9.98×10^{-16}	2.21×10^{-5}	0.98	$[0.91, 1]$	6×10^{-15}	0.74	$[0.62, 1]$	0.000 268
Greek	$<2 \times 10^{-16}$	$<2 \times 10^{-16}$	1	$[0.95, 1]$	$<2 \times 10^{-16}$	1	$[0.95, 1]$	$<2 \times 10^{-16}$
Hebrew	$<2 \times 10^{-16}$	$<2 \times 10^{-16}$	1	$[0.95, 1]$	$<2 \times 10^{-16}$	1	$[0.95, 1]$	$<2 \times 10^{-16}$
Hindi	$<2 \times 10^{-16}$	3.43×10^{-8}	1	$[0.95, 1]$	$<2 \times 10^{-16}$	0.78	$[0.66, 1]$	2.6×10^{-5}
Hungarian	0.127	$<2 \times 10^{-16}$	0.66	$[0.54, 1]$	0.0135	1	$[0.95, 1]$	$<2 \times 10^{-16}$
Indonesian	$<2 \times 10^{-16}$	$<2 \times 10^{-16}$	1	$[0.95, 1]$	$<2 \times 10^{-16}$	1	$[0.95, 1]$	$<2 \times 10^{-16}$
Irish	0.982	$<2 \times 10^{-16}$	0.09	$[0.04, 1]$	1	1	$[0.95, 1]$	$<2 \times 10^{-16}$
Italian	$<2 \times 10^{-16}$	$<2 \times 10^{-16}$	1	$[0.94, 1]$	4.44×10^{-16}	1	$[0.94, 1]$	4.44×10^{-16}
Japanese	$<2 \times 10^{-16}$	$<2 \times 10^{-16}$	1	$[0.95, 1]$	$<2 \times 10^{-16}$	1	$[0.95, 1]$	$<2 \times 10^{-16}$
Korean	$<2 \times 10^{-16}$	$<2 \times 10^{-16}$	1	$[0.95, 1]$	$<2 \times 10^{-16}$	0.98	$[0.92, 1]$	1.55×10^{-15}
Latin	3.97×10^{-9}	3.51×10^{-11}	0.79	$[0.67, 1]$	1.79×10^{-5}	0.85	$[0.75, 1]$	6.92×10^{-8}
Latvian	1.14×10^{-6}	$<2 \times 10^{-16}$	0.76	$[0.65, 1]$	5.68×10^{-5}	1	$[0.95, 1]$	$<2 \times 10^{-16}$
Lithuanian	0.000 234	$<2 \times 10^{-16}$	0.62	$[0.5, 1]$	0.0492	0.98	$[0.91, 1]$	6×10^{-15}
Marathi	1	6.7×10^{-13}	0.18	$[0.1, 1]$	1	0.9	$[0.81, 1]$	6.42×10^{-10}
Norwegian	$<2 \times 10^{-16}$	$<2 \times 10^{-16}$	1	$[0.94, 1]$	1.42×10^{-14}	1	$[0.94, 1]$	2.22×10^{-16}
Old Church Slavonic	1	0.000 429	0.19	$[0.1, 1]$	1	0.73	$[0.62, 1]$	0.000 343
Persian	$<2 \times 10^{-16}$	$<2 \times 10^{-16}$	1	$[0.94, 1]$	2.22×10^{-16}	1	$[0.95, 1]$	$<2 \times 10^{-16}$
Polish	3.57×10^{-8}	$<2 \times 10^{-16}$	0.8	$[0.69, 1]$	4.35×10^{-6}	1	$[0.95, 1]$	$<2 \times 10^{-16}$
Portuguese	0.008 14	$<2 \times 10^{-16}$	1	$[0.95, 1]$	$<2 \times 10^{-16}$	1	$[0.95, 1]$	$<2 \times 10^{-16}$
Romanian	$<2 \times 10^{-16}$	$<2 \times 10^{-16}$	1	$[0.95, 1]$	$<2 \times 10^{-16}$	1	$[0.95, 1]$	$<2 \times 10^{-16}$
Russian	$<2 \times 10^{-16}$	$<2 \times 10^{-16}$	1	$[0.94, 1]$	4.44×10^{-16}	1	$[0.94, 1]$	2.22×10^{-16}
Serbian	$<2 \times 10^{-16}$	$<2 \times 10^{-16}$	1	$[0.94, 1]$	2.22×10^{-16}	1	$[0.95, 1]$	$<2 \times 10^{-16}$
Slovak	6.14×10^{-6}	$<2 \times 10^{-16}$	0.67	$[0.54, 1]$	0.0129	1	$[0.95, 1]$	$<2 \times 10^{-16}$
Slovenian	1.79×10^{-5}	$<2 \times 10^{-16}$	0.8	$[0.69, 1]$	7.01×10^{-6}	1	$[0.95, 1]$	$<2 \times 10^{-16}$
Spanish	5.09×10^{-13}	$<2 \times 10^{-16}$	1	$[0.94, 1]$	8.88×10^{-16}	1	$[0.95, 1]$	$<2 \times 10^{-16}$
Swedish	$<2 \times 10^{-16}$	$<2 \times 10^{-16}$	0.98	$[0.91, 1]$	6×10^{-15}	1	$[0.95, 1]$	$<2 \times 10^{-16}$
Tamil	5.43×10^{-13}	1	1	$[0.94, 1]$	1.78×10^{-15}	0.26	$[0.16, 1]$	1
Telugu	8.2×10^{-7}	1	0.8	$[0.69, 1]$	7.01×10^{-6}	0.22	$[0.13, 1]$	1
Turkish	6.95×10^{-7}	7.49×10^{-15}	0.88	$[0.78, 1]$	1.62×10^{-8}	0.94	$[0.86, 1]$	2.76×10^{-12}
Ukrainian	5.79×10^{-15}	$<2 \times 10^{-16}$	0.87	$[0.77, 1]$	6.54×10^{-9}	1	$[0.95, 1]$	$<2 \times 10^{-16}$
Urdu	1	7.27×10^{-11}	0.1	$[0.04, 1]$	1	0.85	$[0.74, 1]$	2.02×10^{-7}
Vietnamese	0.002 74	$<2 \times 10^{-16}$	0.54	$[0.41, 1]$	0.333	1	$[0.95, 1]$	$<2 \times 10^{-16}$

Table S2: Per-language results in Study 1. For each language, we show the following: (1) p-values obtained from a one-sided t-test, for the null that the mean predictability/parseability of random grammars is at least as high as that of the real grammar. (2) Results from one-sided binomial tests, for the null that the the real grammar is better than at most 50% of random grammars. In addition to the p-value, we report point estimate and 95% confidence interval for the fraction of random grammars that have values below real grammars.

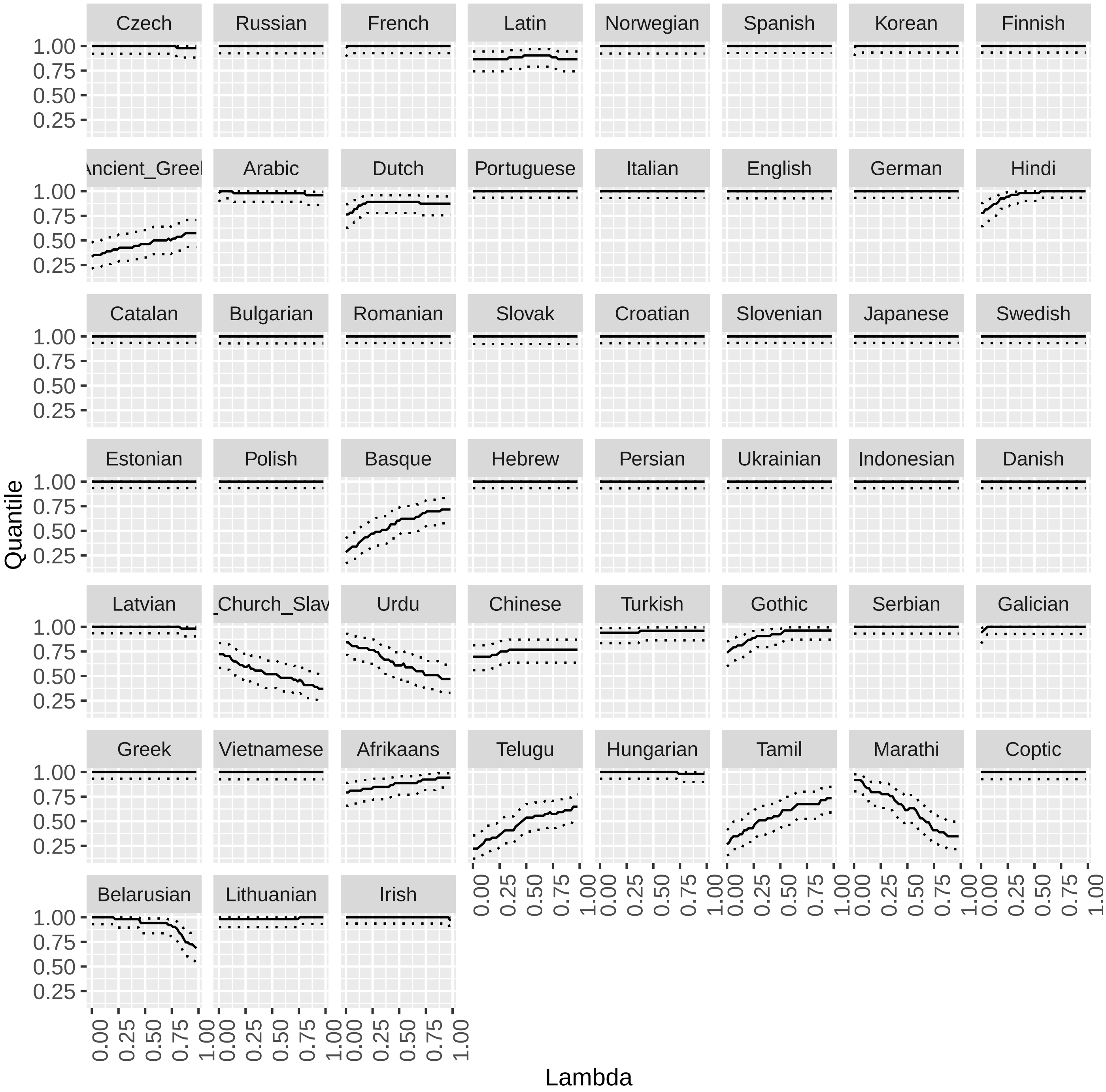

Figure S3: Optimality of real grammars for efficiency, compared to baselines, across values of λ: The x-axis shows $\lambda \in [0, 1)$, the y-axis shows the fraction of baselines that have lower efficiency than the real grammar at this value of λ, with 95% confidence bands obtained from a two-sided binomial test.

	Mean β	SD	Lower 95% CrI	Upper 95% CrI
Efficiency ($\lambda = 0.9$)	-5.88	1.08	-8.28	-3.97
Predictability	-3.48	0.88	-5.42	-1.85
Parseability	-5.55	1.08	-7.80	-3.67

Table S3: Models estimating the log-odds of a random baseline grammar improving over a real grammar in efficiency ($\lambda = 0.9$), surprisal, or parseability, with random effects for languages and language families. The strongly negative estimates of β confirm that, across languages and language families, real grammars improve over most baselines in predictability, parseability, and overall efficiency. This model shows that the optimization observed in Study 1 cannot be attributed to family-specific effects.

This analysis is similar to that reported by Zaslavsky et al. [31] in a study of color names; they found that observed color naming systems have higher efficiency than almost all baseline systems at *a specific value of* λ. Here, we have shown that grammars tend to be more efficient than baselines across most values of λ.

We further confirm this in Figure S4: We plot the real and optimized grammars together with a kernel density estimate of the distribution of baseline grammars. We add lines connecting those points that have the same efficiency R_λ as the real grammar, at very low ($\lambda = 0.0$, dotted line) and very high ($\lambda = 0.9$, dashed line) values of λ. Grammars to the bottom/left of this lines have lower efficiency than the real grammar, at these two given values of λ. The distribution of baseline grammars is largely to the bottom/left of at least one of the two lines, and often to the bottom/left of both lines. This highlights that, even when the real grammar does not appear strongly optimized at all for one individual component, it may still be more efficient than all baselines.

S3.4 Parseability and Surprisal Metrics for Observed Orders and Extracted Grammars

In Table S4, we report parsing and surprisal metrics that are commonly used in the NLP literature, both for the originally observed orders in the corpora, and the corpora ordered according to the real grammars as extracted and expressed in our grammar formalism. We observe similar performance on observed orders and the extracted grammars, across all metrics. We note that, while our parsing model is based on the strongest available dependency parsing method from the NLP literature [47, 48, 49], the parsing metrics here are mostly below the best numbers reported with this architecture [48] due to the use of an unlexicalized parsing model.

S3.5 Impact of Tree Structures on Optimality and Estimated Frontier

Language-Dependence of Tree Structure Distribution Unlike similar efficiency studies in the domain of lexical semantics [22, 28, 31], we did not derive a single universal bound for the efficiency across all 51 languages in Study 1; instead, we constructed optimized grammars individually for each language. In this section, we show why this is necessary: The efficiency of a grammar crucially depends on the tree structure distribution, and this tree structure distribution is language-specific. To show this, we compared the efficiency of the real grammar of English and Japanese with that obtained when applying the real grammar of *the other* language. The results are shown in Figure S5. In both languages, the respective real grammars (crosses) are more efficient than grammars from the other language (squares), even though the grammar from other language still is more efficient than the baseline grammars. This suggests that the grammars of languages, beyond reflecting generic optimization for efficiency across tree structures, may also be specifically optimized for their individual tree structure distributions. Furthermore, it demonstrates that the tree structure distribution, and therefore the optimality of a given grammar, is language-specific.

Estimated Frontier and Corpus Properties An anonymous reviewer notes that the shape of the estimated Pareto frontier (Figure S1) seems to vary between languages, and asks how the tree structure distributions impact the shape of the estimated frontier.

We conducted a series of linear regressions predicting (1) the predictability and parseability of the best grammar optimized for efficiency, (2) the parseability and predictability difference between this end and the end optimized for predictability, (3) the difference between this end and the end optimized for parseability. For more meaningful comparison, we analyzed values normalized for sentence length as reported in Figure S2.

We considered as independent variables the following quantities, computed on the training set: (1) median sentence length, (2) median tree depth, (3) mean arity, i.e., the mean number of dependents of each word[6], (4) the unigram entropy,

[6] The *median* is always 0 or 1 in the available corpora, we thus chose the mean as a more granular measure.

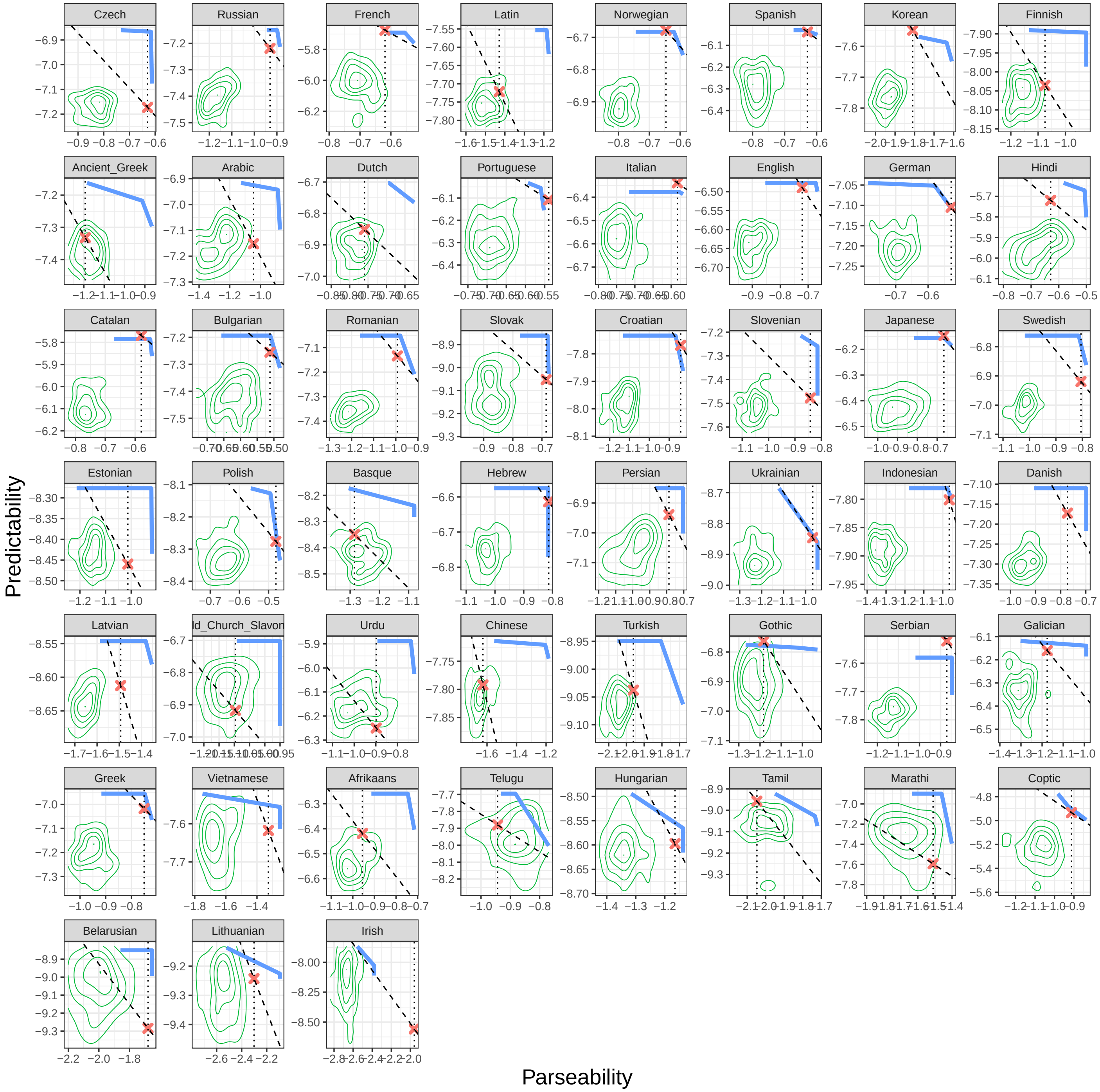

Figure S4: Per-language results as in Figure S2, representing the distribution of baseline grammars with a kernel density estimate. We add lines connecting those points that have the same efficiency as the real grammar at $\lambda = 0.0$ (dotted) and $\lambda = 0.9$ (dashed). Points to the bottom/left of these line have lower efficiency than the real grammar, at the given value of λ.

Language	Observed Orders					Extracted Grammars				
	UAS	LAS	U.Pars.	L.Pars.	Surp.	UAS	LAS	U.Pars.	L.Pars.	Surp.
Afrikaans	0.798	0.757	0.754	1.005	6.341	0.799	0.763	0.738	0.951	6.419
Ancient Greek	0.634	0.539	1.141	1.716	7.345	0.748	0.66	0.801	1.196	7.332
Arabic	0.785	0.726	0.73	1.036	6.872	0.77	0.709	0.759	1.06	7.152
Basque	0.714	0.562	0.879	1.512	8.344	0.736	0.624	0.858	1.293	8.349
Belarusian	0.684	0.606	1.263	1.865	9.127	0.675	0.615	1.21	1.73	9.285
Bulgarian	0.883	0.815	0.378	0.62	7.15	0.894	0.846	0.336	0.497	7.255
Catalan	0.864	0.806	0.457	0.681	5.691	0.873	0.838	0.447	0.586	5.769
Chinese	0.593	0.554	1.301	1.68	7.682	0.594	0.547	1.234	1.617	7.792
Coptic	0.829	0.749	0.634	1.041	4.869	0.84	0.772	0.597	0.891	4.933
Croatian	0.796	0.725	0.696	1.081	7.766	0.816	0.771	0.611	0.869	7.769
Czech	0.824	0.763	0.558	0.858	7.156	0.853	0.813	0.519	0.63	7.173
Danish	0.801	0.75	0.73	1.017	7.043	0.841	0.802	0.55	0.764	7.173
Dutch	0.839	0.782	0.573	0.897	6.826	0.835	0.792	0.57	0.808	6.851
English	0.834	0.788	0.552	0.837	6.396	0.843	0.806	0.498	0.728	6.489
Estonian	0.742	0.602	0.814	1.344	8.371	0.784	0.709	0.681	0.997	8.46
Finnish	0.728	0.616	0.814	1.306	7.959	0.754	0.686	0.755	1.07	8.035
French	0.856	0.8	0.493	0.752	5.72	0.873	0.832	0.425	0.617	5.675
Galician	0.777	0.718	0.77	1.213	6.12	0.774	0.718	0.784	1.175	6.16
German	0.832	0.777	0.53	0.84	7.09	0.896	0.859	0.337	0.523	7.105
Gothic	0.72	0.596	0.869	1.424	7.038	0.755	0.641	0.781	1.217	6.763
Greek	0.829	0.773	0.609	0.89	7.1	0.834	0.804	0.577	0.765	7.018
Hebrew	0.829	0.759	0.588	0.944	6.61	0.835	0.776	0.545	0.836	6.614
Hindi	0.867	0.791	0.38	0.642	5.599	0.861	0.803	0.486	0.614	5.72
Hungarian	0.741	0.622	0.909	1.419	8.572	0.758	0.678	0.855	1.18	8.597
Indonesian	0.8	0.749	0.685	1.062	7.735	0.818	0.767	0.616	0.969	7.801
Irish	0.659	0.542	1.244	2.122	7.772	0.721	0.598	1.023	1.84	8.558
Italian	0.858	0.802	0.471	0.736	6.342	0.879	0.839	0.391	0.588	6.338
Japanese	0.872	0.766	0.389	0.726	6.092	0.877	0.782	0.385	0.696	6.146
Korean	0.623	0.438	1.09	1.898	7.476	0.632	0.459	1.077	1.804	7.548
Latin	0.606	0.492	1.238	2.005	7.735	0.733	0.621	0.873	1.446	7.722
Latvian	0.65	0.53	1.121	1.767	8.629	0.658	0.597	1.07	1.493	8.612
Lithuanian	0.522	0.418	1.614	2.576	9.725	0.546	0.479	1.562	2.295	9.243
Marathi	0.719	0.57	1.006	1.809	7.203	0.76	0.631	0.896	1.42	7.594
Norwegian	0.859	0.801	0.447	0.761	6.678	0.879	0.829	0.378	0.653	6.678
Old Church Slavonic	0.748	0.619	0.79	1.342	7.304	0.794	0.676	0.672	1.089	6.917
Persian	0.814	0.755	0.632	0.869	6.908	0.828	0.78	0.587	0.803	6.939
Polish	0.852	0.782	0.461	0.725	8.389	0.91	0.858	0.357	0.481	8.276
Portuguese	0.869	0.817	0.443	0.676	6.049	0.891	0.847	0.346	0.536	6.109
Romanian	0.806	0.712	0.671	1.123	7.074	0.813	0.737	0.619	0.977	7.134
Russian	0.782	0.696	0.706	1.146	7.155	0.809	0.742	0.607	0.923	7.219
Serbian	0.825	0.757	0.617	0.992	7.556	0.832	0.766	0.576	0.894	7.521
Slovak	0.831	0.772	0.543	0.849	9.199	0.849	0.817	0.495	0.696	9.053
Slovenian	0.798	0.713	0.705	1.112	7.498	0.841	0.788	0.595	0.836	7.478
Spanish	0.855	0.789	0.484	0.777	6.246	0.869	0.825	0.429	0.637	6.039
Swedish	0.823	0.752	0.606	0.979	6.839	0.849	0.796	0.519	0.808	6.919
Tamil	0.658	0.572	1.245	1.896	9	0.663	0.565	1.438	1.857	8.957
Telugu	0.882	0.651	0.359	1.081	7.9	0.888	0.715	0.481	0.882	7.88
Turkish	0.58	0.423	1.376	2.119	8.966	0.572	0.448	1.358	1.959	9.038
Ukrainian	0.789	0.716	0.714	1.101	8.826	0.799	0.753	0.673	0.953	8.846
Urdu	0.816	0.736	0.617	0.984	5.771	0.822	0.756	0.58	0.893	6.25
Vietnamese	0.627	0.583	1.142	1.601	7.536	0.696	0.653	0.986	1.345	7.618

Table S4: Parsing and Surprisal metrics for observed orders (left), and for corpora ordered according to extracted real grammars (right). UAS and LAS refer to *Unlabeled* and *Labeled Attachment Scores*, respectively, indicating what fraction of words is assigned the correct head (UAS) or the correct head and relation label (LAS) when choosing heads and labels assigned the highest probability $p_\phi(\text{head}_i, \text{label}_i|u, i)$ (Equation S6) by the parser. U.Pars refers to the average value of $-\log p_\phi(\text{head}_i|u, i)$, which is a measure of the difficulty of recovering the raw tree structure, without relation labels. L.Pars refers to the average value of $-\log p_\phi(\text{head}_i, \text{label}_i|u, i)$, measuring the difficulty of recoovering tree structures including relation labels. Note that L.Pars corresponds to $\text{H}[\mathcal{T}|\mathcal{U}]$ normalized by the number of words. Finally, Surp. refers to the average word-by-word surprisal, which corresponds to the predictability measure $\text{H}[\mathcal{U}]$ normalized by the number of words.

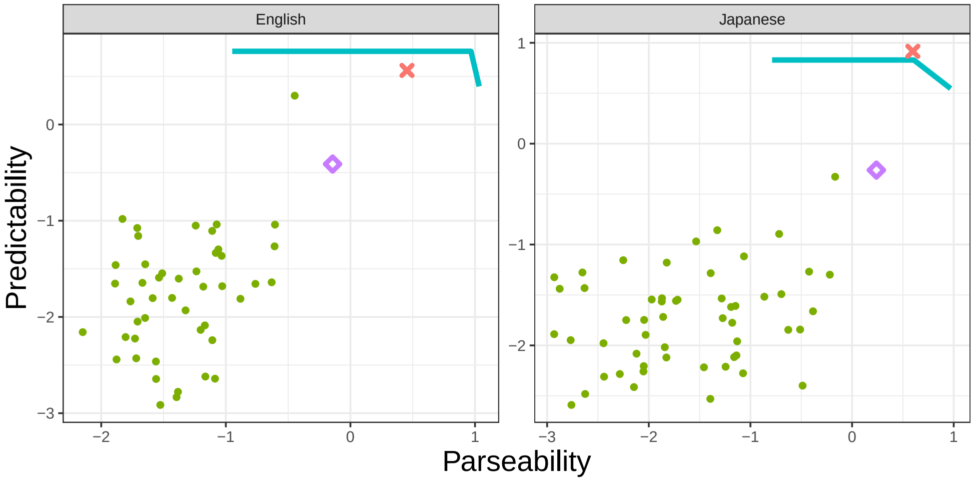

Figure S5: Languages L have grammars optimized specifically for the tree structure distributions of L: We show the real (cross) and baseline (dots) grammars for English and Japanese, together with the estimated Pareto frontier. Additionally, we plot the efficiency values obtained when applying the Japanese grammar to English tree structures (purple square, left), and when applying the English grammar to Japanese tree structures (purple square, right). In both languages, the respective real grammars (crosses) are more efficient than grammars from the other language (squares), even though the grammar from the other language still is more efficient than the baselines. This suggests that the grammars of languages are specifically optimized for their individual tree structure distributions.

and (5) the logarithm of the overall number of sentences.

These independent variables measure the complexity of syntactic strutures (1-3), the diversity of the vocabulary (4), and the amount of data available for constructing the neural network models (5). The resulting regressions are shown in Table S5.

Among factors measuring the complexity of syntactic structures (predictors (1)-(3)), the strongest effect is a positive effect of arity on predictability ($\beta = 7.76$, $SE = 1.51$, $p < 0.001$), suggesting that structures with more dependents per head lead to higher achievable predictability. In contrast, we observe little evidence for an impact of sentence length or tree depth. We also observe an effect of unigram entropy (4), showing that datasets with more diverse vocabulary reduce both predictability and parseability.[7] Finally, larger amounts of training data (5) lead to higher estimated predictability and parseability—this is expected, as more training data enables better statistical estimation of the distribution of sentences and syntactic structures. More training data also increases the difference between the efficiency-optimal and the predictability-optimal ends of the estimated curve, suggesting that more training data enables more precise estimation of the different extremes of the curve.

These results show that general quantitative properties of the available syntactic structures partially account for variation in the achievable parseability and predictability values. Note that at least some of these quantitative properties are impacted by factors external to the syntax of a language, e.g., the unigram entropy may be impacted by the genre of available texts. This result again suggests that it may not be possible to derive a language-independent bound on syntactic efficiency, in contrast with studies of semantic typology where there is a language-invariant parameterization of the possible meanings (e.g., [22, 26, 31]).

S4 Supplementary Analyses for Study 2

S4.1 Correlation between Universals and Efficiency

In Figure S6, we plot efficiency, parseability, and predictability (all are z-scored within language, as in Study 1) as a function of the number of satisfied correlations, for the real grammars of the 51 languages.

We found very similar results using Spearman's rank correlation (Efficiency: $\rho = 0.59$, $p = 9.8 \cdot 10^{-6}$; Parseability: $\rho = 0.55$, $p = 4.7 \cdot 10^{-5}$; Predictability: $\rho = 0.36$, $p = 0.012$).

[7] For predictability, a similar result about vocabulary size and estimated surprisal across many languages is reported by [50].

Predictor	Optimized for Efficiency			Distance to Pred. End			Distance to Pars. End		
	β	SE	t	β	SE	t	β	SE	t
(Intercept)	-9.9	1.15	-8.61***	0.02	0.09	0.18	-0.74	0.2	-3.61***
(1) MedianSentenceLength	0.06	0.04	1.54	-0.01	0	-1.85	0	0.01	-0.13
(2) MedianTreeDepth	-0.2	0.14	-1.42	0.02	0.01	2.06*	0.01	0.03	0.57
(3) MeanArity	7.76	1.51	5.15***	-0.04	0.12	-0.38	0.31	0.27	1.16
(4) UnigramEntropy	-1.11	0.11	-9.91***	0.02	0.01	2.63*	0.05	0.02	2.59*
(5) log(SentenceCount)	0.54	0.05	9.84***	-0.02	0	-4.89***	-0.01	0.01	-0.84
(Intercept)	-1.5	0.72	-2.07*	0	0.23	0	0.17	0.08	2.22*
(1) MedianSentenceLength	0.03	0.02	1.35	-0.01	0.01	-1.12	0	0	-1.39
(2) MedianTreeDepth	-0.11	0.09	-1.27	0.03	0.03	0.9	0.01	0.01	0.76
(3) MeanArity	0.68	0.95	0.71	-0.17	0.3	-0.55	-0.06	0.1	-0.56
(4) UnigramEntropy	-0.3	0.07	-4.31***	-0.06	0.02	-2.52*	-0.02	0.01	-2.11*
(5) log(SentenceCount)	0.28	0.03	7.97***	0.04	0.01	3.61***	0	0	0.78

Significance levels: * : $p < 0.05$, ** : $p < 0.01$, *** : $p < 0.001$

Table S5: Linear regression models predicting the position of the estimated Pareto frontier, from quantitative properties of the available syntactic tree structures. The top half provides models predicting predictability values, the bottom half provides models predicting parseability values. Columns correspond to the three pairs of independent variables defined in the text: predictability/parseability for the best grammar optimized for efficiency, the predictability/parseability distance to the end optimized for predictability, and the predictability/parseability distance to the end optimized for parseability.

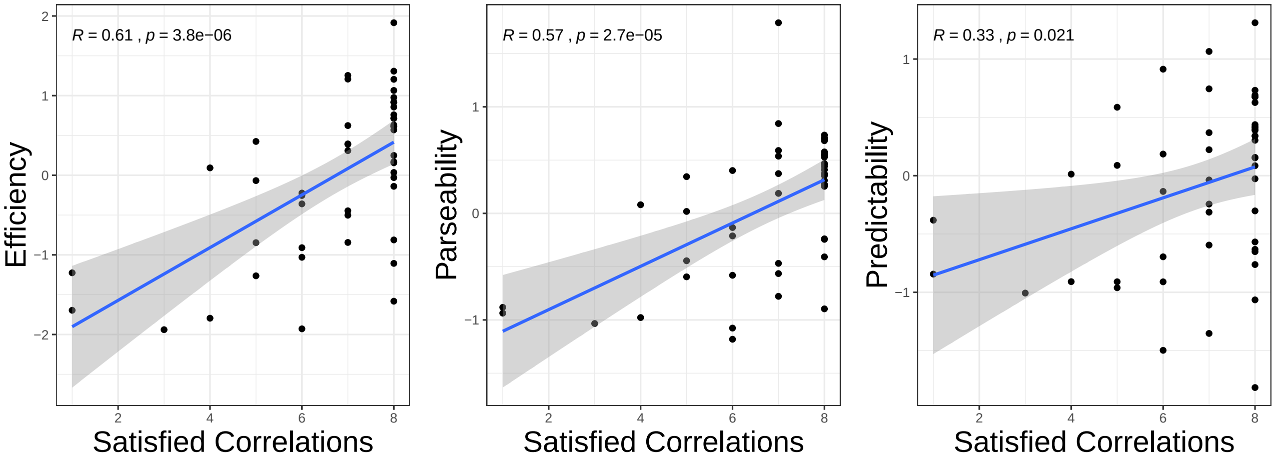

Figure S6: Correlation between the number of satisfied correlations (x-axis) and efficiency, parseability, and predictability (y-axis), for the 51 real languages.

S4.2 Predictions for Individual Languages

We show predictions for the eight correlations on the level of individual languages in Figure S7. We obtained these predictions for individual languages and each of the eight relations as follows. For each language and each of the objective functions (efficiency, predictability, parseability), we considered the optimized grammar that yielded the best value of this objective function among the eight optimized grammars (i.e., the grammar where the optimization procedure had been most successful). We interpreted this grammar as verb-object or object-verb depending on the order in the real grammar of the language.

S4.3 Regression for Predicted Correlations

Bayesian Mixed-Effects Regression We modeled the probabilities $p_{L,j}$ that a grammar optimized for data from language L satisfies the j-th correlation ($j = 1, ..., 8$) using a multilevel logistic model [51], with random intercepts for the language for whose data the grammar had been optimized, and for its language family, annotated according to `http://universaldependencies.org/`. Formally,

$$logit(p_{L,j}) = \alpha_j + u_{L,j} + v_{f_L,j} \tag{12}$$

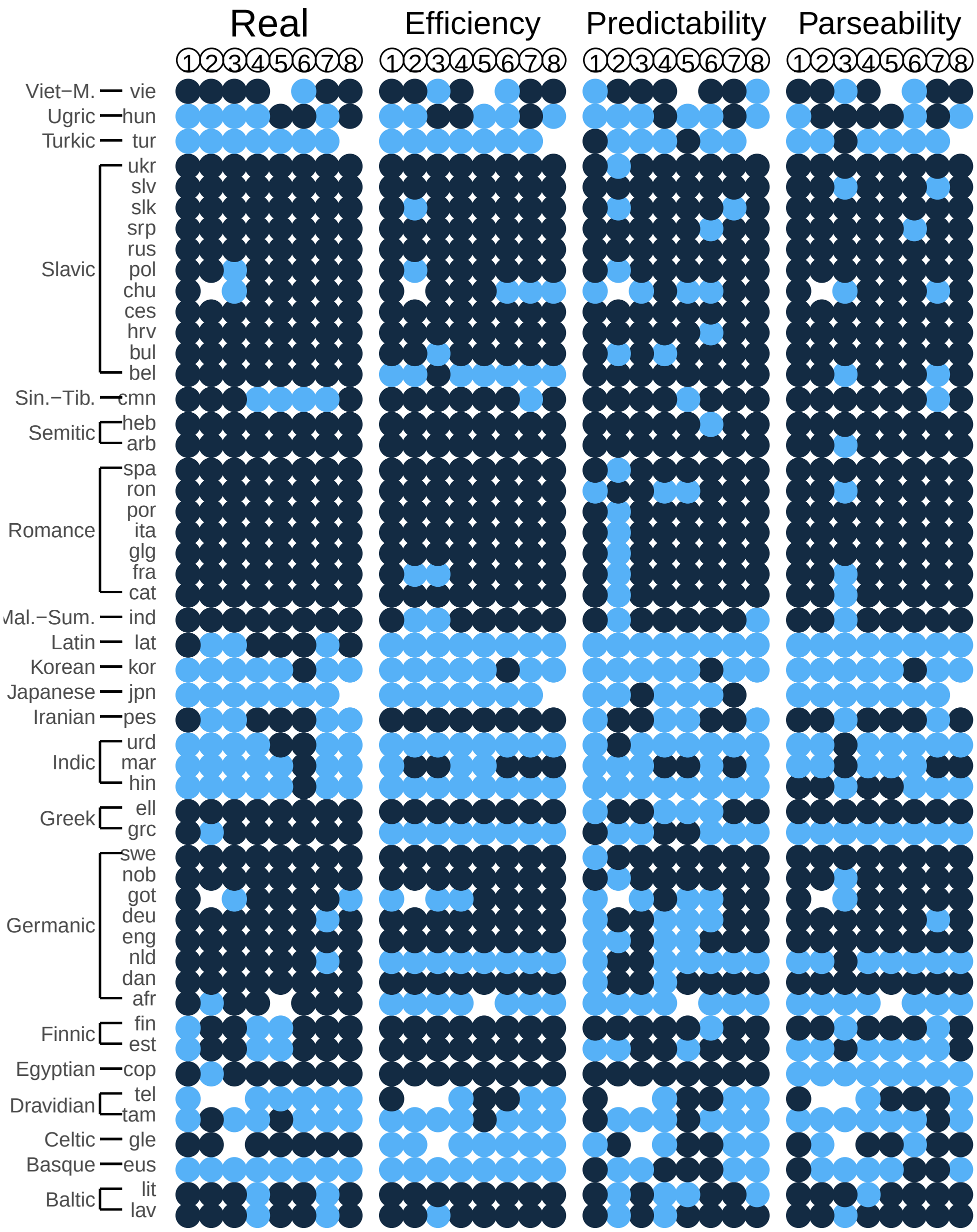

Figure S7: Order of the eight correlates across 51 languages, in the real grammars (left) and predicted by optimizing for efficiency, predictability, parseability (right). Dark blue: Verb patterner *precedes* object patterner (English, Arabic, ...). Light blue: Verb patterner *follows* object patterner (Japanese, Hindi , ...). White cells indicate that the relation is not annotated in the dataset for the given language.

	Prevalence	Bayesian			Frequentist			
		Mean	SD	$p(\beta \leq 0)$	β	SE	z	p
①	0.779	1.449	0.273	$< 1 \times 10^{-4}$	1.395	0.222	6.277	3.5×10^{-10}
②	0.678	0.761	0.171	1.0×10^{-4}	0.784	0.135	5.796	6.8×10^{-9}
③	0.696	1.003	0.424	0.012	0.943	0.342	2.753	0.006
④	0.782	1.586	0.318	$< 1 \times 10^{-4}$	1.512	0.251	6.013	1.8×10^{-9}
⑤	0.793	1.505	0.327	$< 1 \times 10^{-4}$	1.434	0.272	5.281	1.3×10^{-7}
⑥	0.757	1.133	0.43	0.006	1.072	0.352	3.041	0.002
⑦	0.748	1.093	0.388	0.003	1.026	0.322	3.185	0.001
⑧	0.911	3.854	0.878	$< 1 \times 10^{-4}$	3.823	0.782	4.887	1.0×10^{-6}

Table S6: Detailed results for Bayesian and Frequentist mixed-effects analyses for the eight correlations. We show (1) the raw prevalence of each correlation in the optimized grammars (8 grammars for each of the 51 languages), (2) for the Bayesian analysis, we provide posterior mean and SD of β, and the posterior probability that β has the opposite sign, (3) for the Frequentist analysis, we provide the point estimate, SE, z, and p-values (2-sided). The frequentist analysis confirms the results of the Bayesian analysis.

where f_L is the language family of L. The intercepts α_j ($j = 1, ...8$) encode the population-level prevalence of the correlations when controlling for differences between datasets from different languages and language families; $u_{L,j}$, $v_{f_L,j}$ encode per-language and per-family deviations from the population-level intercept α_j.

Following the recommendations of [52, 53], we used as a very weakly informative prior a Student's t prior with $\nu = 3$ degrees of freedom, mean 0, and scale $\sigma = 10$ (i.e., the PDF p is $\frac{1}{\sigma} p_3(x/\sigma)$, where p_3 is the PDF of the t-distribution with $\nu = 3$). We used this prior for $\alpha_j, \sigma_{L,j}, \tau_{L,j}$. A correlation that holds in 90% of cases would correspond to an intercept $\alpha \approx 2.19$ in the logistic model, well within the main probability mass of the prior.

We modeled full covariance matrices of per-language and per-family random intercepts over all eight correlations. We placed an LKJ prior ($\eta = 1$) on these matrices, as described in [53]. We used MCMC sampling implemented in Stan [54, 55] using the R package `brms` [56]. We ran four chains, with 5000 samples each, of which the first 2500 were discarded as warmup samples. We confirmed convergence using $\hat{R}$ and visual inspection of chains [51].

We obtained the posterior density plots in Figure 6 (Main Paper) and in Figure (S7) by applying the logistic transformation ($x \mapsto \frac{1}{1+\exp(-x)}$) to the posterior samples of α_j (12). As the logistic transformation is inverse to the logit transform (12), this corresponds to the posterior distribution of the prevalence (between 0.0 and 1.0) of each correlation, controlling for languages and language families.

Robustness To ascertain the robustness of our results, we also conducted a frequentist analysis using `lme4` [57]. For each of the correlations, we conducted a logistic mixed-effects analysis predicting whether a grammar satisfies the correlation, with random effects of language and language family. The results are shown in Table S6 together with those of the Bayesian analysis. The frequentist analysis agrees with the Bayesian model; all eight correlations are predicted to hold in more than half of the optimized grammars ($p < 0.01$ each).

Note that the Bayesian analysis also estimates a posterior distribution of the number of satisfied correlations (see Figure S8), providing an elegant solution to the multiple-comparisons problem arising from analysing the eight correlation.

S4.4 Comparing Efficiency to its Components

In Figure S8, we plot the posterior distribution of the number of correlations predicted to hold in most optimized grammars, as obtained from the Bayesian regression. For each posterior sample, we say that the j-th correlation holds if the value of α_j in that posterior sample is positive. In the figure, we plot the fraction of posterior samples in which a given number of correlations is satisfied. In addition to grammars optimized for efficiency, we also report the result for grammars optimized for predictability and for parseability alone. Efficiency predicts all eight correlations with high posterior probability; predictability and parseability alone do not.

S4.5 Results on all UD Relations

In this section, we provide the predicted prevalence of correlations between the *obj* dependency and all UD dependency types, along with the expected prevalence according to typological studies. We also report results for grammars optimized for predictability and parseability individually.

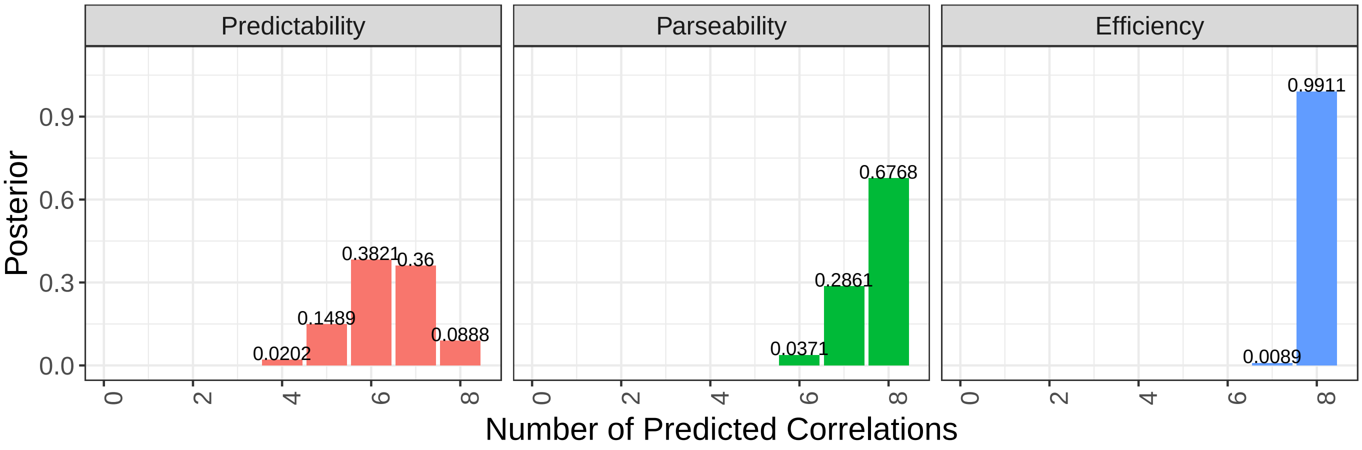

Figure S8: Posterior of the number of correlations correctly predicted by efficiency and its components, in the Bayesian multivariate mixed-effects logistic regression with random effects for languages and language families. We show results for grammars optimized for only Predictability (left), only Parseability (center), and full Efficiency (right).

We considered all UD syntactic relations occurring in at least two of the 51 languages. In Table S7, we present the data for the eight correlations discussed in the main paper, and for those other relations for which the typological literature provides data.[8] Additionally, in Table S8 we present data for the other UD relations, for which either no typological data is available, or which are not linguistically meaningful.

S4.6 Previous Experiments

In Table S9 we report the results of our two previous, preregistered, simulations[9] together with results from the main experiment. These experiments all had the same setup described in Section S6, which was fixed before starting simulations; differences are that (1) one simulation places fully equal weight on parseability and predictability ($\lambda = 1.0$), and (2) the final experiment uses three random seeds per grammar. Results across all three experiments agree; jointly optimizing grammars for parseability and predictability produces all eight correlations.

S4.7 Comparison to other Formalizations of Greenberg's Correlations

We followed Dryer [1] in treating Greenberg's correlations as pairwise correlations with verb–object order. While Greenberg's original study [6] also formalized most of these as correlations with verb–object order, a few were formalized as correlations between other relations that are only indirectly related to verb-object order (e.g., Universal 22 linking the position of the standard of comparison to the order of adpositions).

Justeson and Stephens [58] conducted a log-linear analysis on typological judgments of 147 languages, constructing an undirected graphical model modeling correlations among any pair of six syntactic relations (verb–object, adposition–noun, noun–genitive, noun–relative clause, noun–adjective, verb–subject). Results from their analysis suggested that some relations are directly correlated with the verb–object order, whereas other relations are only indirectly correlated with it. In particular, in their analysis, the noun–genitive relation (corresponding to Correlation ④ here) was not directly correlated with the verb–object correlation; instead, the typologically observed correlation was explained through correlations between the noun–genitive relation and other relations (such as the adposition–noun relation) that directly correlate with the verb–object relation. Note that this does not contradict the statement that verb–object and noun–genitive relations correlate; it shows that the observed correlation can be explained through a chain of other correlations.

Since the set of syntactic relations examined here is different from that examined by Justeson and Stephens [58], we cannot directly compare the predictions of efficiency optimization with their results. Nonetheless, we can show that efficiency optimization is compatible with a picture of Greenberg's correlation as a network of pairwise correlations among

[8] The *aux* syntactic relation in UD has the auxiliary (verb-patterner) as its dependent, and has direction *opposite* to the auxiliary-verb relation ③. Therefore, this relation is *anti-correlated* with the verb-object relation, while ③ is *correlated*. For simplicity, we display this as a corelation in this table.

[9] `http://aspredicted.org/blind.php?x=8gp2bt`, `https://aspredicted.org/blind.php?x=bg35x7`. For the results of the Locality simulations described in the first preregistration, see the Dependency Length Minimization results in Table S15, with discussion in Section S11.

	Relation	Real	Pred	Pars	Efficiency	Expected Prevalence
①	lifted_case					> 50% [1]
②	lifted_cop					> 50% [1]
③	aux					> 50% [1]
④	nmod					> 50% [1]
⑤	acl					> 50% [1]
⑥	lifted_mark					> 50% [1]
⑦	obl					> 50% [1]
⑧	xcomp					> 50% [1]
	advcl					> 50% [6, 106]
	ccomp					> 50% (cf. [107])
	csubj					> 50% (cf. [107])
	nsubj					See Section S1
	amod					≈ 50% [1]
	nummod					≈ 50% [108, 89A, 83A]

Table S7: Predictions on UD relations with predictions from the typological literature. The first section contains the eight correlations discussed in the main paper (See Section S1); the second section provides other relations for which predictions are available. The 'Real' column provides the prevalence among the 51 languages in the Universal Dependencies data. We provide posterior prevalences for grammars optimized for Efficiency, and for grammars optimized for Pars(eability) and Pred(ictability) alone, obtained from the Bayesian mixed-effects analysis controlling for languages and language families (as in Figure 6 of the main paper). In the last column, we indicate what prevalence is expected according to the typological literature.

Relation	Real	Pred	Pars	Efficiency	Expected Prevalence
appos					Unknown
lifted_cc					Unknown
expl					Unknown
iobj					Unknown
vocative					Unknown
compound					Uninterpretable
det					Uninterpretable
dislocated					Uninterpretable
dep					Uninterpretable
advmod					Uninterpretable
conj					UD Artifact
discourse					UD Artifact
fixed					UD Artifact
flat					UD Artifact
goeswith					UD Artifact
list					UD Artifact
orphan					UD Artifact
parataxis					UD Artifact
reparandum					UD Artifact

Table S8: Predictions on UD relations for which no predictions are available in the typological literature. "Uninterpretable" UD relations are those which collapse so many different linguistic relationships that they are not linguistically meaningful. "UD artifact" relations are those whose order is determined strictly by UD parsing standards, such that their order is not linguistically meaningful: these include dependencies such as the connection between two parts of a word that have been separated by whitespace inserted as a typo (*goeswith*). We provide results for grammars optimized for Efficiency, and for grammars optimized for Pars(eability) and Pred(ictability) alone.

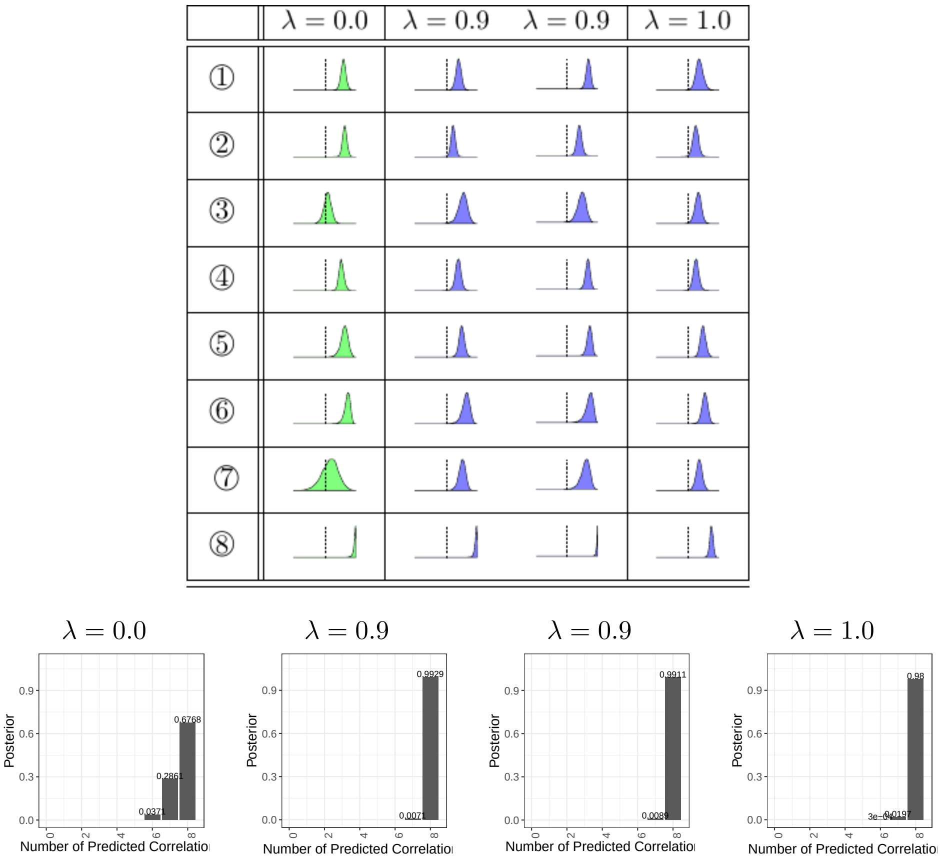

Table S9: Results from optimization experiments for different values of λ, including our two previous preregistered experiments (Section S4.6). For comparison, we also show results for $\lambda = 0$, corresponding to optimizing for parseability only (same results as reported in Tables (S7-S8)). For $\lambda = 0.9$, we report results from one preliminary preregistered experiment (center left) and the final experiment (center right). For $\lambda = 1.0$, we report the other preliminary preregistered experiment. Giving similar weight to parseability and predictability – that is, λ close to 1 – results in more accurate word order predictions than choosing a small value of λ such as $\lambda = 0.0$. Note that λ cannot take values smaller than zero, or greater than one, see Section S2.2.

different syntactic relations, and in particular the result that the correlation between the verb–object and noun–genitive relations is mediated through other correlations.

First, we directly test the optimized grammars for two additional correlations found by Justeson and Stephens [58]: For the relations examined here, beyond correlations with verb–object order, they found additional correlations between (1) the noun–genitive and adposition–noun dependencies, and (2) between the noun–relative clause and adposition–noun dependencies, *beyond* the correlation mediated through the individual correlations with the verb–object dependency. We ran the same Bayesian logistic mixed-effects analysis for these two correlations. Results are shown in Figure S10. Both correlations are very strongly supported by grammars optimized for efficiency.

Second, we directly applied the log-linear analysis described by Justeson and Stephens [58] to optimized grammars. We represent each grammar via the directions $v_1, \dots v_9$ of the nine relations indicated in Table 1 of the main paper (verb–object, and ①-⑧), we coded these as -0.5 for Japanese-like order, and $+0.5$ for Arabic-like order. This analysis models the relative frequency $p_{(v_1,\dots,v_9)}$ of a particular configuration of such a configuration $(v_1, \dots, v_9)$ by a log-linear model:

$$\log p_{(v_1,\dots,v_9)} = u_0 + \sum_{i=1}^{9} u_i v_i + \sum_{i,j \in C} u_{i,j} v_i v_j \tag{13}$$

where C is some set of (unordered) pairs of relations $\in \{1, \dots, 9\}$, modeling those pairs of relations that directly correlate with each other, and where $u_0, i_i, i_{i,j}$ are real-valued parameters. For instance, if all relations directly correlate with the verb–object order, and not with any other relation, C would contain all the unordered pairs containing the verb–object

		Prevalence	Mean	SD	$p(\beta \leq 0)$
G-N (nmod)	N-Adp (lifted_case)	0.919	4.482	1.058	$< 1 \times 10^{-4}$
Rel-N (acl)	N-Adp (lifted_case)	0.898	4.653	1.286	$< 1 \times 10^{-4}$

Table S10: Detailed results for the two correlations found by Justeson and Stephens [58] that do not involve the verb–object dependency, for grammars optimized for efficiency. Both correlations are strongly supported by optimized grammars, holding in about 90% of optimized grammars. Compare Table S6.

relation.

We inferred the best-fitting such model by selecting the pairs in C via forward-selection using AIC. The best-fitting model includes a set C of 13 correlating pairs, with $AIC = 274.18$. This resulting model is shown in Figure S9; following [58], we show those links between nodes that are included in this selected model. In agreement with the results of [58], a network is identified in which all relations are connected at least indirectly, but several relations are not directly connected to the verb–object relation: In particular, in accordance with the typological data analysed by [58], the observed correlation between the verb–object and noun–genitive relation is entirely mediated through correlations with other relations (adposition–noun and verb–adpositional phrase) that directly correlate with the verb–object relation. A difference is that, in our analysis and unlike the analysis by [58], the noun–relative clause dependency is not directly linked to the verb–object relation; this might be because our analysis takes a different set of relations into account compared to [58].

We also note that, unlike our mixed-effects models, this log-linear model does not have random effects, as we found that adding random effects to the log-linear model led to nonconvergence. This means that it does not account for differences in the tree structures between languages and language families; as a result, the mixed-effects analyses for individual correlation pairs may be more conservative than this log-linear model. Future work should replicate the analysis of [58] on a larger typological database and with more relations, to enable a direct comparison with the network structure predicted by efficiency optimization.

S5 Creating Optimized Grammars

In this section, we describe the method we employ for creating grammars that are optimized for efficiency, and how we extract grammars describing the actual ordering rules of languages. We carry out grammar optimization in an extended space of grammars that interpolates continuously between different grammars (Section S5.1). More specifically, we include probabilistic relaxations of grammars, which describe probability distributions over different ways of ordering a syntactic structure into a sentence. This makes efficiency a *differentiable* function of the grammar parameters, and enables efficient optimization with stochastic gradient descent, as we describe in Section S5.3.

This method addresses a major challenge noted in previous work optimizing grammars, namely that the predictability (and parseability) of an individual sentence depends on the entire distribution of the language. Previously, Gildea and Jaeger [59] optimized grammars for dependency length and trigram surprisal using a simple hill-climbing method on the grammar parameters, which required reestimating the trigram surprisal model in every iteration. Such a method would be computationally prohibitive for efficiency optimization, as it would require reestimating the neural network models after every change to the grammar, which would amount to reestimating them hundreds or thousands of times per grammar. Our method, by allowing for the use of stochastic gradient descent, addresses this challenge, as we describe in Section S5.3.

S5.1 Differentiable Ordering Grammars

We extended the parameter space of grammars by continuously interpolating between grammars, making efficiency a *differentiable* function of grammar parameters. The parameters of such a **differentiable word order grammar** are as follows. For each dependency label type τ, we have (1) a **Direction Parameter** $a_\tau \in [0, 1]$, and (2) a **Distance Parameter** $b_\tau \in \mathbb{R}$. Each dependent is ordered on the left of its head with probability a_τ and to the right with probability $1 - a_\tau$. Then for each set of co-dependents $\{s_1, \ldots, s_n\}$ placed on one side of a head, their order from left to right is determined by iteratively sampling from the distribution $\text{softmax}(b_{\tau_1}, \ldots, b_{\tau_n})$ (for dependents preceding the head) or $\text{softmax}(-b_{\tau_1}, \ldots, -b_{\tau_n})$ (for dependents following the head) (for the definition of Softmax, see [60, p. 184]) without replacement.

If $a_\tau \in \{0, 1\}$, and the distances between values of b_τ (for different τ) become very large, such a differentiable grammar becomes deterministic, assigning almost full probability to exactly one ordering for each syntactic structure. In this case, the grammar can be converted into an equivalent grammar of the form described in Materials and Methods, by extracting a single parameter in $[-1, 1]$ for each relation τ.

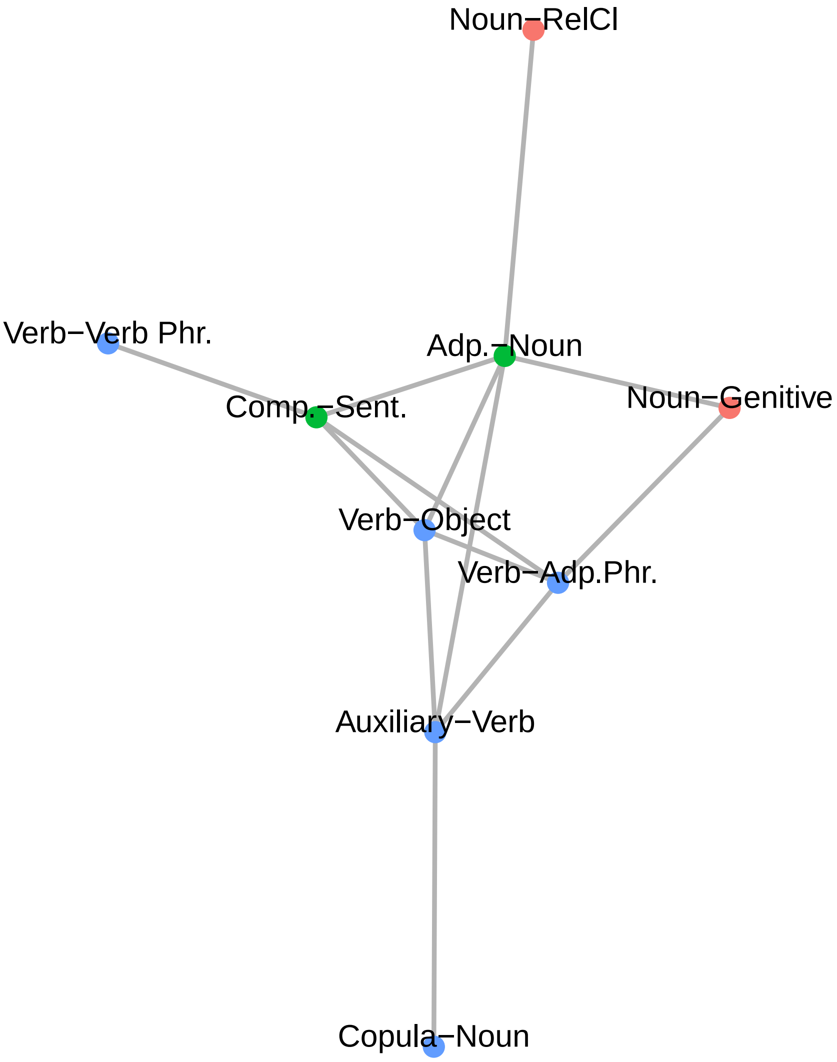

Figure S9: Network of pairwise correlations among the nine syntactic relations examined in this study, estimated from grammars optimized for efficiency, identified using a log-linear model following Justeson and Stephens [58]. The verb–object relation is at the center of the network. Relations between verbs and their dependents are colored in blue; relations between nouns and their dependents are colored in red; other relations are colored in green.

	English			Japanese		
Relation	Par.	a_τ	b_τ	Par.	a_τ	b_τ
object (*obj*)	0.1	0.04	−1.46	−0.1	0.99	−0.72
oblique (*obl*)	0.3	0.13	1.25	−0.3	0.99	0.73
case (*lifted_case*)	0.2	0.07	−0.89	−0.2	0.92	0.02

Figure S10: Sample Coefficients from grammars extracted from the real English and Japanese orderings (Section S5.2), for the relations occurring in Figure 3 (Main Paper). We show parameters in $[-1, 1]$ for deterministic word order grammars as described in *Materials and Methods*, and the coefficients (a_τ, b_τ) for corresponding differentiable ordering grammars. For the deterministic grammars ('Par.'), positive coefficients indicate that the dependent will be placed after the head. For the differentiable grammars, $a_\tau > 0.5$ indicates predominance of ordering of dependents before heads, and larger b_τ indicates greater distance between head and dependent.

We provide an example in Figure S10, illustrating grammar parameters for the relations in Figure 3 of the main paper.

Note that the grammatical formalism simplifies some aspects of the word order regularities of natural languages. For instance, it does not represent cases where ordering varies between main and embedded clauses, as it does not condition ordering decisions on the larger context. It also does not model nonprojective orderings, which—while generally rare—do occur in many languages. More complex and powerful ordering grammar models have been proposed [61, 62]; however, they have similar limitations, and for our purposes, the model adopted here has the advantage of being simple and interpretable.

S5.2 Extracting Grammars from Datasets

We extract grammars for each actual language by fitting a differentiable ordering grammar maximizing the likelihood of the observed orderings. To prevent overfitting, we regularize each a_τ, b_τ with a simple Bayesian prior $logit(a_\tau) \sim \mathcal{N}(0, 1)$, $b_\tau \sim \mathcal{N}(0, 1)$. We implemented this regularized optimization as mean-field ELBO variational inference in Pyro [63]. We then extract the posterior means for each parameter a_τ, b_τ, and convert the resulting differentiable grammar into an ordinary ordering grammar.

We validated the extracted grammars by comparing the dominant orders of six syntactic relations that are also annotated in the World Atlas of Linguistic Structures (WALS, [64]). Among the eight Greenbergian correlations that we were able to test, five are annotated in WALS: adpositions, complementizers, relative clauses, genitives, and oblique PPs. In Table S11, we compare our grammars with WALS on these five relations, and the verb–object relation. WALS has data for 74% of the entries[10], and lists a dominant order for 91% of these. The grammars we extracted from the corpora agree with WALS in 96 % of these cases.

S5.3 Optimizing Grammars for Efficiency

In this section, we describe how we optimized grammar parameters for efficiency. A word order grammar can be viewed as a function $\mathcal{L}_\theta$, whose behavior is specified by parameters θ, which takes an unordered dependency tree t as input and produces as output an ordered sequence of words $u = \mathcal{L}_\theta(t)$ linearizing the tree. More generally, if $\mathcal{L}_\theta$ is a differentiable ordering grammar (Section S5.1), then $\mathcal{L}_\theta(t)$ defines a *probability distribution* $p_{\mathcal{L}_\theta}(u|t)$ over ordered sequences of words u. In the limit where $\mathcal{L}_\theta$ becomes deterministic, the distribution $p_{\mathcal{L}_\theta}(u|t)$ concentrates on a single ordering u.

Recall the definition of efficiency

$$R_{Eff} := R_{Pars} + \lambda R_{Pred}, \tag{14}$$

where

$$R_{Pars} := \mathrm{I}[\mathcal{U}, \mathcal{T}] = \sum_{t,u} p(t, u) \log \frac{p(t|u)}{p(t)} \tag{15}$$

$$R_{Pred} := -\mathrm{H}[\mathcal{U}] = \sum_{u} p(u) \log p(u), \tag{16}$$

where $t \sim \mathcal{T}$ is the distribution over syntactic structures as found in databases of the language, and $u \sim p_{\mathcal{L}_\theta}(u|t)$ denotes the corresponding linearized sentences.

[10] Serbian and Croatian are listed as a single language Serbian-Croatian in WALS. In the table, we compare those with the grammar we extracted for Croatian, noting that it fully agrees with the Serbian grammar.

Language	Objects		Adpositions		Compl.		Rel.Cl.		Genitive		PP	
Afrikaans	DH	?	HD	?	HD	?	–	?	HD	?	HD	?
Anc.Grk.	DH	?	HD	?	HD	?	HD	?	HD	?	HD	?
Arabic	HD	HD	HD	HD	HD	HD	HD	?	HD	HD	HD	HD
Basque	DH	DH	DH	DH	DH	DH	DH	DH	DH	DH	DH	DH
Belarusian	HD	*	HD	?	HD	?	HD	HD	HD	HD	HD	*
Bulgarian	HD	HD	HD	HD	HD	HD	HD	HD	HD	*	HD	HD
Catalan	HD	HD	HD	HD	HD	?	HD	HD	HD	HD	HD	?
Chinese	HD	HD	HD	*	DH	?	DH	DH	DH	DH	DH	DH
Coptic	HD	HD	HD	HD	HD	HD	HD	HD	HD	HD	HD	HD
Croatian	HD	HD	HD	HD	HD	HD	HD	?	HD	*	HD	?
Czech	HD	HD	HD	HD	HD	HD	HD	HD	HD	*	HD	?
Danish	HD	HD	HD	HD	HD	HD	HD	HD	*HD*	DH	HD	HD
Dutch	DH	*	HD	HD	HD	HD	HD	HD	HD	HD	DH	*
English	HD	HD	HD	HD	HD	HD	HD	HD	HD	*	HD	HD
Estonian	HD	HD	DH	DH	HD	HD	*DH*	HD	DH	DH	HD	HD
Finnish	HD	HD	DH	DH	HD	HD	*DH*	HD	DH	DH	HD	HD
French	HD	HD	HD	HD	HD	HD	HD	HD	HD	HD	HD	HD
Galician	HD	?	HD	?	HD	?	HD	?	HD	?	HD	?
German	HD	*	HD	HD	HD	HD	HD	HD	HD	HD	DH	*
Gothic	HD	?	HD	?	HD	?	HD	?	HD	?	HD	?
Greek	HD	HD	HD	HD	HD	HD	HD	HD	HD	HD	HD	?
Hebrew	HD	HD	HD	HD	HD	HD	HD	HD	HD	HD	HD	?
Hindi	DH	DH	DH	DH	HD	HD	DH	*	DH	DH	DH	?
Hungarian	*DH*	HD	DH	DH	HD	HD	HD	*	DH	DH	DH	?
Indonesian	HD	HD	HD	HD	HD	HD	HD	HD	HD	HD	HD	HD
Irish	HD	HD	HD	HD	HD	HD	HD	HD	HD	HD	HD	HD
Italian	HD	HD	HD	HD	HD	HD	HD	HD	HD	HD	HD	?
Japanese	DH	DH	DH	DH	DH	DH	DH	DH	DH	DH	DH	DH
Korean	DH	DH	DH	DH	*HD*	DH	DH	DH	DH	DH	DH	?
Latin	DH	?	HD	?	HD	?	HD	?	HD	?	DH	?
Latvian	HD	HD	HD	HD	HD	HD	HD	HD	DH	DH	DH	?
Lithuanian	HD	HD	HD	HD	HD	HD	HD	HD	DH	DH	DH	?
Marathi	DH	DH	DH	DH	HD	*	DH	DH	DH	DH	DH	?
Norwegian	HD	HD	HD	HD	HD	HD	HD	HD	HD	*	HD	?
O.C.Slav.	HD	?	HD	?	HD	?	HD	?	HD	?	HD	?
Persian	DH	DH	HD	HD	HD	HD	HD	HD	HD	HD	DH	?
Polish	HD	HD	HD	HD	HD	HD	HD	HD	HD	HD	HD	?
Portuguese	HD	HD	HD	HD	HD	?	HD	HD	HD	HD	HD	?
Romanian	HD	HD	HD	HD	HD	HD	HD	HD	HD	HD	HD	?
Russian	HD	HD	HD	HD	HD	HD	HD	HD	HD	HD	HD	?
Serbian	HD	?	HD	?	HD	?	HD	?	HD	?	HD	?
Slovak	HD	?	HD	?	HD	?	HD	?	HD	?	HD	?
Slovenian	HD	HD	HD	HD	HD	?	HD	?	HD	*	HD	?
Spanish	HD	HD	HD	HD	HD	HD	HD	HD	HD	HD	HD	HD
Swedish	HD	HD	HD	HD	HD	HD	HD	HD	*HD*	DH	HD	HD
Tamil	DH	DH	DH	DH	DH	*	*HD*	DH	DH	DH	DH	DH
Telugu	DH	DH	DH	DH	DH	*	DH	DH	DH	DH	DH	?
Turkish	DH	DH	DH	DH	DH	*	DH	DH	DH	DH	DH	DH
Ukrainian	HD	HD	HD	HD	HD	HD	HD	HD	HD	?	HD	?
Urdu	DH	DH	DH	DH	HD	HD	HD	*	DH	DH	DH	?
Vietnamese	HD	HD	HD	HD	*DH*	HD	–	HD	HD	HD	HD	HD

Table S11: Comparing grammars extracted from databases to linguistic judgments in the World Atlas of Linguistic Structures. For each of the six syntactic relation, the first column provides the ordered coded in the extracted grammar; the second column provides the order coded in WALS (DH for dependent-head, HD for head-dependent order). '?' indicates that WALS has no data. $*$ indicates that WALS does not list a dominant order; as Dryer [65] describes, this can mean that neither order is dominant in the language, or that insufficient data was available when compiling WALS. Finally, '–' indicates that the relation does not occur in the Universal Dependencies corpus.

These quantities are estimated using two neural models, as described in Section S6: A **parser** recovers syntactic structures from utterances by computing a distribution $p_\phi(t|u)$, parameterized via parser parameters ϕ. The degree to which a parser with parameters ϕ succeeds in parsing a sentence u with structure t is[11]

$$R^{\phi}_{Pars}(u,t) = \log p_\phi(t|u). \tag{17}$$

A **language model**, with some parameters ψ, calculates the word-by-word surprisal of an utterance:

$$R^{\psi}_{Pred}(u) = \sum_{i=1}^{|u|} \log p_\psi(u_i|u_{1\ldots i-1}). \tag{18}$$

Using this and Gibbs' inequality [66], we can rewrite Efficiency (14), for a given grammar θ, equivalently into the parseability and predictability achieved with the best parser and language models:

$$R^{\theta}_{Eff} := \max_{\phi,\psi} R^{\theta,\phi,\psi}_{Eff}, \tag{19}$$

where we have written

$$R^{\theta,\phi,\psi}_{Eff} := \mathbb{E}_{t\sim\mathcal{T}}\, \mathbb{E}_{u\sim p_{\mathcal{L}_\theta}(u|t)} \left[R^{\phi}_{Pars}(u,t) + \lambda R^{\psi}_{Pred}(u)\right]. \tag{20}$$

In order to find an optimal grammar θ, we thus need to compute

$$\arg\max_{\theta}\ R^{\theta}_{Eff} = \arg\max_{\theta}\ \max_{\phi,\psi} R^{\theta,\phi,\psi}_{Eff}. \tag{21}$$

Importantly, $R^{\theta,\phi,\psi}_{Eff}$ is differentiable in θ, ϕ, ψ:

$$\partial_\theta R^{\theta,\phi,\psi}_{Eff} = \mathbb{E}_t\, \mathbb{E}_{u\sim p_{\mathcal{L}_\theta}(u|t)} \left[[\partial_\theta \log p_{\mathcal{L}_\theta}(u|t)] \cdot \left(R^{\phi}_{Pars}(u,t) + \lambda R^{\psi}_{Pred}(u)\right)\right] \tag{22}$$

$$\partial_\phi R^{\theta,\phi,\psi}_{Eff} = \mathbb{E}_t\, \mathbb{E}_{u\sim p_{\mathcal{L}_\theta}(u|t)} \left[\partial_\phi R^{\phi}_{Pars}(u,t)\right] \tag{23}$$

$$\partial_\psi R^{\theta,\phi,\psi}_{Eff} = \mathbb{E}_t\, \mathbb{E}_{u\sim p_{\mathcal{L}_\theta}(u|t)} \left[\lambda \cdot \partial_\psi R^{\psi}_{Pred}(u)\right], \tag{24}$$

where (22) is derived using the *score-function* or *REINFORCE* theorem [67]. Note that the derivatives inside the expectations on the right hand sides can all be computed using backpropagation for our neural network architectures.

We can therefore apply stochastic gradient descent to jointly optimize θ, ϕ, ψ: In each optimization step, we sample a dependency tree t from the database, then sample an ordering from the current setting of θ to obtain a linearized sentence $\mathbf{w} \sim p_\theta(\cdot|t)$. Then we do a gradient descent step using the estimator given by the expressions in the square brackets in (22-24).

Optimizing for only parseability (or predictability) is very similar—in this case, the terms involving R^{ϕ}_{Pred} (or R^{ψ}_{Pars}) are removed.

At the beginning of the optimization procedure, we initialize all values $a_\tau := 0.5$, $b_\tau := 0$ (except for the *obj* dependency, for which we fix a_τ to 0 or 1, see Section S6). The neural parser and language model are also randomly initialized at the beginning of optimization. Empirically, we observe that optimizing differentiable ordering grammars for efficiency leads to convergence towards deterministic behavior, allowing us to extract equivalent deterministic grammars as described in Section S5.1.

See Section S6, paragraph 'Optimization Details' for the stopping criterion and learning rates used in this optimization scheme.

S6 Neural Network Architectures

In this section, we describe the details of the neural network architectures. Choices follow standard practice in machine learning. All choices, except where explicitly noted otherwise, were made before evaluating word order properties, and the efficiency of real grammars.

[11] Note that, in the definition of R_{Pars} (28), the term $p(t)$ is a constant independent of ϕ and the word order grammar $\mathcal{L}_\theta$; it can therefore be ignored in the optimization process.

Estimating Predictability We choose a standard LSTM language model [68, 69], as such recurrent neural models are the strongest known predictors of the surprisal effect on human processing effort [70, 71]. This model uses a recurrent neural network to compute the predictability of a sentence $u = u_1 ... u_n$[12]:

$$\log p_\psi(u) = \sum_{i=1}^{n} \log p_\psi(u_i | u_{1...i-1}) \tag{25}$$

where ψ are the parameters of the recurrent LSTM network, optimized on training data (see paragraph 'Optimization Details').

We estimate the average predictability of a language as a Monte Carlo estimate on held-out data:

$$R_{Pred} := -\operatorname{H}[\mathcal{U}] = \sum_u p(u) \log p_\psi(u) \approx \frac{1}{|\text{Heldout Data}|} \sum_{u \in \text{Heldout Data}} \log p_\psi(u) \tag{26}$$

by averaging over all sentences u occurring in the corpus.

For computational reasons, we restrict the vocabulary to the most frequent 50,000 words in the treebanks for a given language. Given the moderate size of the corpora, this limit is only attained only for few languages. In each time step, the input is a concatenation of embeddings for the word, for language-specific POS tags, and for universal POS tags. The model predicts both the next word and its language-specific POS tag in each step. Using POS tags is intended to prevent overfitting on small corpora. This choice was made before evaluating the efficiency of real grammars, and before evaluating word order properties.

Estimating Parseability We use a biaffine attention parser architecture [72, 73, 47]. This architecture is remarkably simple: the words of a sentence are encoded into context-sensitive embeddings using bidirectional LSTMs, then a classifier is trained to predict the head for each work. The classifier works by calculating a score for every pair of word embeddings (w_i, w_j), indicating the likelihood that the jth word is the head of the ith word. This is a highly generic architecture for recovering graph structures from strings, and is a simplification of graph-based parsers which reduce the parsing problem to a minimal spanning tree problem [74]. The parseability of a sentence $u = u_1 \dots u_n$ with syntactic structure t is computed as

$$\log p_\phi(t|u) = \sum_{i=1}^{n} \log p_\phi(\text{head}_i, \text{label}_i | u, i) \tag{27}$$

where $\text{head}_i \in \{\text{ROOT}, 1, \dots, n\}$ is the index of the head of u_i in the syntactic structure, and label_i is its syntactic relation as formalized in UD; ϕ denotes the parameters estimated on the training data (see paragraph 'Optimization Details'). The overall parseability is estimated as a Monte Carlo estimate on held-out data:

$$R_{Pars} := \operatorname{I}[\mathcal{U}, \mathcal{T}] = \sum_{t,u} p(t,u) \log \frac{p_\phi(t|u)}{p(t)} \approx \frac{1}{|\text{Heldout Data}|} \sum_{t,u \in \text{Heldout Data}} \log \frac{p_\phi(t|u)}{p(t)} \tag{28}$$

The constant $p(t)$ only depends on the language (but not on the word order rules), and can thus be ignored when comparing different grammars applied to the same language, and when optimizing grammars for a given language; we therefore do not attempt to explicitly estimate it.

To reduce overfitting on small corpora, we choose a delexicalized setup, parsing only from POS tags. Preliminary experiments showed that a parser incorporating word forms overfitted long before the ordering grammar had converged; parsing from POS tags prevents early overfitting. This decision was made before evaluating word order properties.

Hyperparameters Neural network models have hyperparameters such as the number of hidden units, and the learning rate. For predictability and parseability optimization, we first selected hyperparameters on the respective objectives for selected languages on the provided development partitions. These parameters are shown in Table S12. Then, for each language and each objective function, we created eight random combinations of these selected hyperparameter values, and selected the setting that yielded the best value of the respective objective function (efficiency, predictability, parseability) on the language. We then used this setting for creating optimized word order grammars.

All word and POS embeddings are randomly initialized with uniform values from $[-0.01, 0.01]$. We do not use pretrained embeddings [75]; while these could improve performance of language models and parsers, they would introduce confounds from the languages' actual word orders as found in the unlabeled data.

[12]Technically, $u_1 ... u_{n-1}$ are words, and u_n is an end-of-sentence token, to ensure the probability distribution over all sentences is normalized.

Optimization	Learning Rate<br>Momentum	5e-6, 1e-5, 2e-5, 5e-5<br>0.8, 0.9
Language Model	Learning Rate<br>Dropout Rate<br>Embedding Size (Words)<br>Embedding Size (POS)<br>LSTM Layers<br>LSTM Dimensions	0.5, 0.1, 0.2<br>0.0, 0.3, 0.5<br>50<br>20<br>2<br>128
Parser	Learning Rate<br>Dropout Rate<br>Embedding Size<br>LSTM Layers<br>LSTM Dimensions	0.001<br>0.2<br>100<br>2<br>200

Table S12: Hyperparameters

Improved Unbiased Gradient Estimator We employ two common variance reduction methods to improve the estimator (22), while keeping it unbiased. For predictability, note that the surprisal of a specific word only depends on the preceding words (not on the following words), and thus only depends on ordering decisions made up to that word. We represent the process of linearizing a tree as a dynamic stochastic computation graph, and use these independence properties to apply the method described in Schulman et al. [76] to obtain a version of (22) with lower variance. Second, we use a word-dependent moving average of recent per-word losses (the word's surprisal in the case of predictability, and the negative log-probability of the correct head and relation label in the case of parseability) as control variate [67]. These two methods reduce the variance of the estimator and thereby increase the speed of optimization and reduce training time, without biasing the results. For numerical stability, we represent $a_\tau \in [0, 1]$ via its logit $\in \mathbb{R}$. Furthermore, to encourage exploration of the parameter space, we add an entropy regularization term [77] for each Direction Parameter a_τ, which penalizes a_τ values near 0 or 1. The weight of the entropy regularization was chosen together with the other hyperparameters.[13]

These techniques for improving (22) are well-known in the machine learning literature, and we fixed these before evaluating optimized grammars for word order properties.

Optimization Details We update word order grammar parameters θ using Stochastic Gradient Descent with momentum. For the language model parameters ϕ, we use plain Stochastic Gradient Descent without momentum, as recommended by Merity et al. [78]. For the parser parameters ψ, we use Adam [79], following Dozat et al. [47]. The learning rates and other optimization hyperparameters were determined together with the other hyperparameters.

All corpora have a predefined split in training and held-out (development) sets. We use the training set for optimizing parameters, and apply Early Stopping [80] using the held-out set.

For **estimating the parseability or predictability** of a given grammar, we optimize the neural model on data ordered according to this grammar, and report the parseability/predictability on the held-out set to avoid overfitting to the training set. For Early Stopping, we evaluate on the held-out set at the end of every epoch.

For **optimizing grammars**, we jointly apply gradient descent to the grammar parameters and the neural models, using the gradient estimator (22-24). For Early Stopping, we evaluate on the held-out set in intervals of 50,000 sentences, using a Monte-Carlo estimate of $R_{Eff}^{\theta,\phi,\psi}$ (S5.3), sampling a single linearized sentence for each syntactic structure in the held-out set. When reporting the parseability/predictability of an optimized grammar, we evaluate these values for its fully deterministic version (Section S5.1) to allow fair comparison with baseline grammars.

The choice of optimization methods and the stopping criterion were fixed before we investigated language efficiency or word order correlations.

Optimized Grammars As described in the main paper, for each language, we created 8 optimized languages for each optimization criterion. We enforced balanced distribution of object–verb and verb–object ordering among optimized languages by fixing a_τ for the *obj* dependency to be 0.0 in four of these languages, and 1.0 in the other four. This maximizes statistical precision in detecting and quantifying correlations between the verb–object relation and other relations.

For efficiency optimization, for each grammar, we ran efficiency optimization with three different random seeds, selecting among these the seed that yielded the best overall efficiency value. We did this in order to control for possible variation

[13] Explored values: 0.0001, 0.001.

across random seeds for the stochastic gradient descent optimization method. As described in our preregistration `http://aspredicted.org/blind.php?x=ya4qf8`, this choice was made after conducting a preliminary version of Study 2 reported in Section S4.6; results reported there show qualitatively identical results regarding the prediction of the eight word order correlations by efficiency optimization.

S7 Robustness to different language models and parsers

Here we take up the question of the extent to which our results are dependent on the particular parser and language model used in the optimization process. We want to know: when we optimize a word order grammar for efficiency, have we produced a language which is highly efficient *in general*, or one which is highly efficient *for a specific parser*? We wish to argue that natural language syntax is optimized for efficiency in general, meaning that syntactic trees are highly recoverable from word orders in principle. If it turns out that our optimized languages are only optimal for a certain parser from the NLP literature, then we run the risk of circularity: it may be that the reason this parser was successful in the NLP literature was because it implicitly encoded word order universals in its inductive biases, and thus it would be no surprise that languages which are optimized for parseability also show those universals.

In this connection, we note that the parser and language model architectures we use are highly generic, and do not encode any obvious bias toward natural-language-like word orders. The LSTM language model is a generic model of sequence data which is also been used to model financial time series [81] and purely theoretical chaotic dynamical systems [82]; the neural graph-based parser is simply solving a minimal spanning tree problem [74]. Nevertheless, it may be the case that a bias toward word order universals is somehow encoded implicitly in the hyperparameters and architectures of these models.

Here we address this question by demonstrating that our languages optimized for efficiency are also optimal under a range of different language models and parsers. These results show that our optimization process creates languages in which strings are generally predictable and informative about trees, without dependence on particular prediction and parsing algorithms.

S7.1 CKY Parsers

We constructed simple Probabilistic Context-Free Grammars (PCFGs) from corpora and word order grammars, using a simplified version of the models of [83] (Model 1). In our PCFGs, each head independently generates a set of left and right dependents. We formulate this as a PCFG where each rule has the form:

$$\text{POS}_H \rightarrow \text{POS}_H\ \text{POS}_D$$

for head-initial structures, and

$$\text{POS}_H \rightarrow \text{POS}_D\ \text{POS}_H$$

for head-final structures, where each symbol is a POS tag. Thus, POS tags act both as terminals and as nonterminals.

We estimated probabilities by taking counts in the training partition, and performing Laplace smoothing with a pseudocount $\alpha = 1$ for each possible rule of this form. For such a PCFG, exact parsing is possible using Dynamic Programming, and specifically the CKY algorithm [84].

This parsing strategy is very different from the neural graph-based parser: While the graph-based parser solves a minimum spanning tree problem, the CKY algorithm uses dynamic programming to compute the exact probabilities of trees given a sentence, as specified by the generative model encoded in the PCFG. Second, while the graph-based neural parser uses machine learning to induce syntactic knowledge from data, the CKY parser performs exact probabilistic inference. In this sense, the CKY algorithm does not have any architectural biases in itself. On the other hand, the PCFG makes severely simplifying independence assumptions, compared to the universal approximation capabilities of neural network-based systems.

We used the CKY algorithm to compute the syntactic ambiguity $\text{H}[\mathcal{T}|\mathcal{U}]$ on the validation partition of the English and Japanese UD corpora, for random and optimized ordering grammars. Results (Figure S11) show that optimized grammars are more parseable than baseline grammars, for exact parsing of a simple PCFG.

S7.2 Distorted graph-based parsers

In this section, we provide evidence against the idea that the graph-based parser might have a built-in bias toward certain kinds of orderings.In particular, we address the idea that the graph-based parser might have a bias toward parses involving short dependencies, which we call a **locality bias**. We address this by changing the order in which the parser sees words,

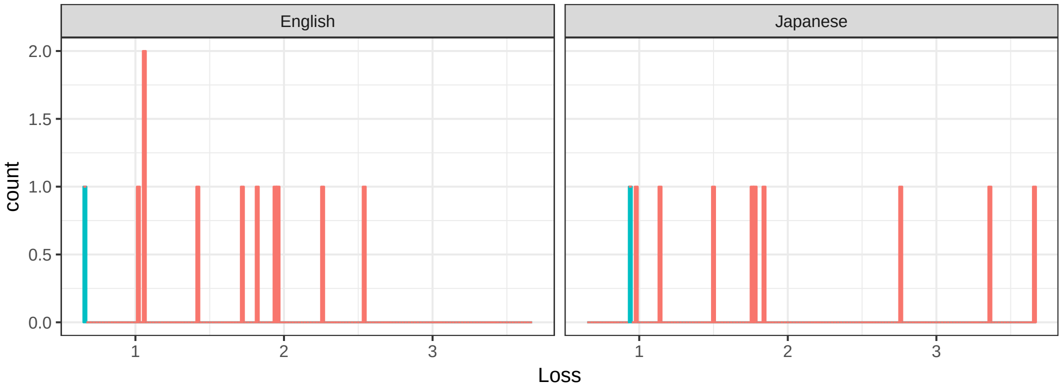

Figure S11: Parsing loss H[$\mathcal{T}|\mathcal{U}$] (lower is better) computed by a simple CKY parser, for random word order grammars (red) and word order grammars optimized for efficiency (blue). We report H[$\mathcal{T}|\mathcal{U}$] normalized by sentence length.

such that word adjacency in the input to the parser does not correspond to linear adjacency in the true utterance. If the parser has a locality bias, then this bias will be disrupted when it sees words in these distorted orders. We consider a number of possible distorted orders:

Even–odd order. A sequence of n words originally ordered as $w_1w_2w_3w_4 \cdots w_n$ is reordered by separating the even and odd indices: $w_2w_4w_6 \cdots w_{n-1}w_1w_3w_5 \cdots w_n$ (assuming n odd). Therefore all words that are adjacent in the original order will be separated by a distance of $\approx n/2$ in the distorted order, while all words of distance 2 in the original order will become adjacent.

Interleaving order. In interleaving ordering, a sequence originally ordered as $w_1w_2w_3 \cdots w_n$ is split in half at the middle (index $m = \lceil n/2 \rceil$), and the two resulting sequences are interleaved, yielding $w_1w_mw_2w_{m+1}w_3w_{m+3} \cdots w_n$. Thus all words that were originally adjacent will have distance 2 in the distorted order, with the intervening word coming from a very distant part of the sentence.

Inwards order. A sequence originally ordered as $w_1w_2w_3 \cdots w_{n-1}w_n$ is ordered from the edges of the string inwards, as $w_1w_nw_2w_{n-1} \cdots w_{\lceil n/2 \rceil}$. This corresponds to folding the string in on itself once, or equivalently, splitting the sequence in half at the middle, then interleaving the two resulting sequences after reversing the second one. The result is that the most non-local possible dependencies in the original order become the most local dependencies in the distorted order.

Lexicographic order. A sequence is reordered by sorting by POS tags, and randomizing the order within each block of identical POS tags. To each word, we then add a symbol encoding the original position in the sequence. For instance

PRON VERB PRON

may be reordered as

PRON 1 PRON 3 VERB 2

or

PRON 3 PRON 1 VERB 2

The numbers are provided to the parser as atomic symbols from a vocabulary ranging from 1 to 200; numbers greater than 200 (which may occur in extremely long sentences) are replaced by an out-of-range token.

The result is that distance between words in the input is not indicative at all of the presence of absence of syntactic relations between them.

Experiments Using English and Japanese data, we trained parsers for ten random word order grammars and for the best grammar optimized for efficiency, with the input presented in each of the distorted orderings. Resulting parsing scores are shown in Figure S12. In all settings, the language optimized for efficiency achieved lower parsing loss (i.e., higher parseability) than random ordering grammars, showing that the parser's preference for optimized languages cannot be attributed to a locality bias.

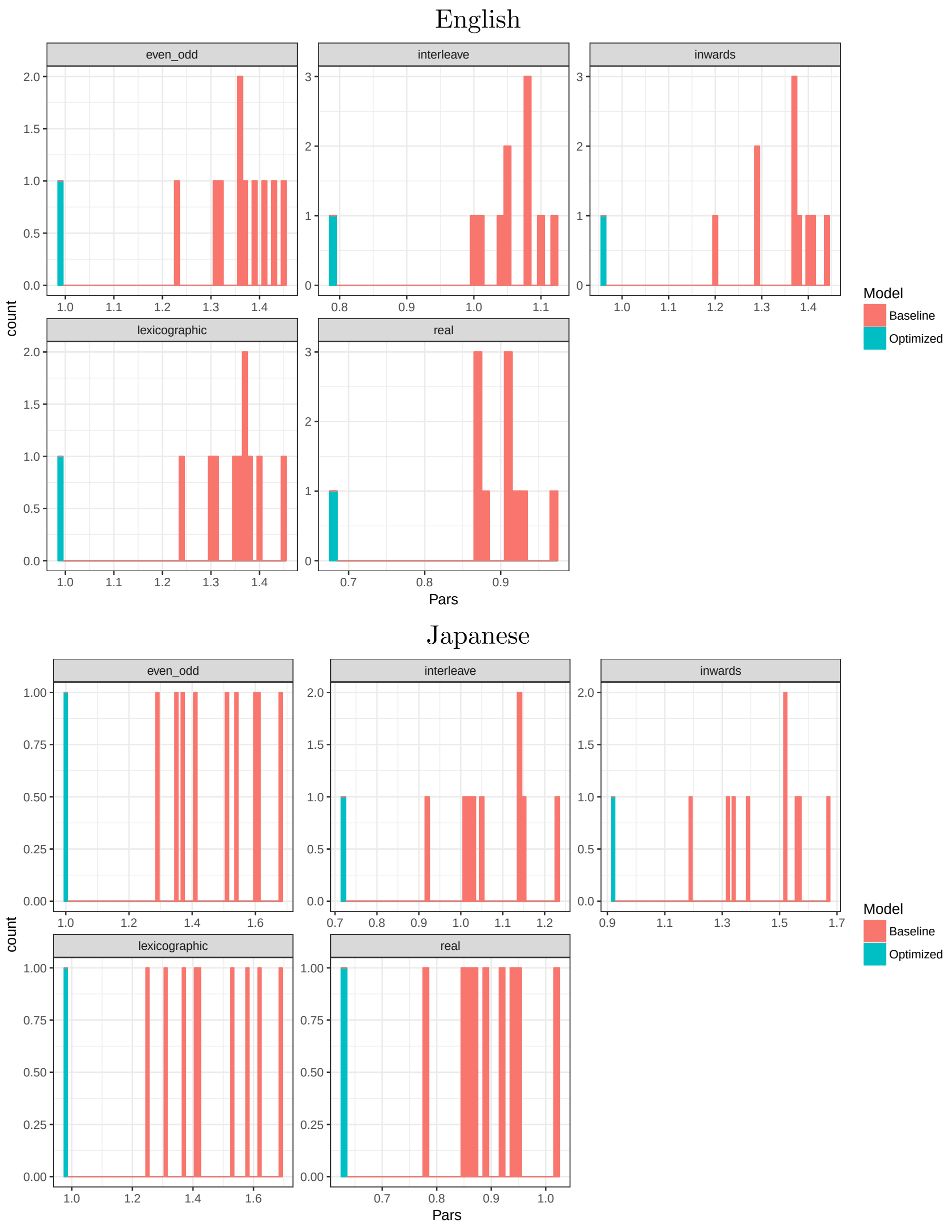

Figure S12: Parseability of baseline grammars and grammars optimized for efficiency, in English (top) and Japanese (bottom), measured by parsing loss $\mathrm{H}[\mathcal{T}|\mathcal{U}]$ (lower is better), for the four distorted orderings, and the actual orderings ('real'). We report $\mathrm{H}[\mathcal{T}|\mathcal{U}]$ normalized by sentence length.

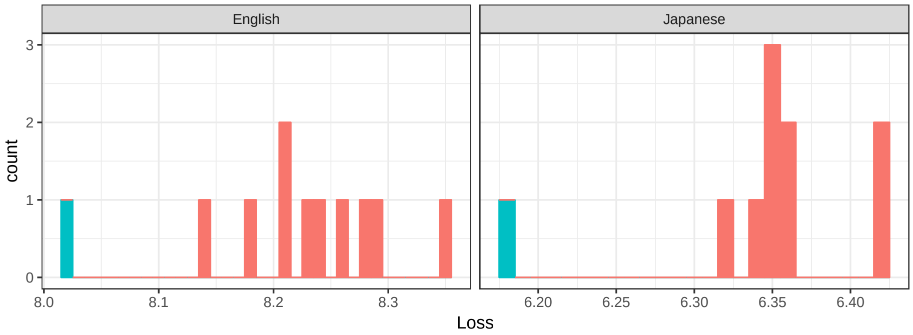

Figure S13: Surprisal (i.e., negative predictability, lower is better) computed from Bigram model, on English and Japanese data ordered according to random ordering grammars (red) and ordering grammars optimized for efficiency (blue).

S7.3 n-gram language models

We model predictability using LSTM language models, which are are the strongest known predictors of the surprisal effect on human processing effort [70, 71]. In previous work, such as [59], predictability has often been measured using n-gram models.

Here, we show that languages optimized for LSTM predictability are also optimal for n-gram predictability. Specifically, we constructed bigram models with Kneser-Ney smoothing [85, 86]. A bigram model predicts each word taking only the previous word into account. This contrasts with LSTMs, which take the entire context into consideration. Thus, bigram models and LSTMs stand on opposing ends of a spectrum of language models taking more and more aspects of the context into account.

We estimated language models on the training partitions, and used the validation partitions to estimate surprisal. We conducted this for ten random and the best optimized ordering grammars on English and Japanese data. Results (Figure S13) show that languages optimized for efficiency are also optimal for a bigram language model.

S8 Other Methods of Estimating Efficiency and Constructing Baselines in Study 1

S8.1 Lexicalized Models

In Study 1, we calculate parseability on the part-of-speech level, and also add part-of-speech tags when calculating predictability. These choices are intended to prevent early overfitting during the grammar optimization process (Section S6). However, such unlexicalized parsers are less accurate than parsers taking acual word-forms into account, and adding part-of-speech tags might provide additional disambiguation that is absent in the original word-level input. Here, we show that these limitations do not affect conclusions from Study 1, by replicating Study 1 with both parsers and language models operating entirely on word forms, without POS tags. Results are shown in Figure S14 and Table S13. We compare real and baseline grammars; here, we do not have an estimate of the Pareto frontier, as the grammar optimization process uses part-of-speech tags (Section S6). In agreement with the previous results (Figure S1), real grammars are mostly to the top right of their corresponding baselines. We further confirm this in Figure S15, which shows that most real grammars have higher efficiency than most baselines across permissible values of λ. In fact, comparing Figure S15 to Figure S3 suggests that optimality of real grammars is *more* pronounced when modeling predictability and parseability fully on the level of word forms.

S8.2 Original UD Format

As described in *Materials and Methods*, we follow [5] in applying automated conversion of tree structures to a more standard formalism, modifying each treebank by inverting dependencies of types *cc*, *case*, *cop*, and *mark*. This converted version is intended to more closely reflect assumptions about syntactic structure shared across a wide range of linguistic theories, addressing criticism of the Universal Dependencies representation [87].

In this section, we provide evidence that this conversion does not affect our results by replicating the comparison between real and baseline grammars in Study 1 using the original Universal Dependencies (UD) representation. As in Study 1, we represented the real grammars by extracting grammars from the observed orderings; for each language, we

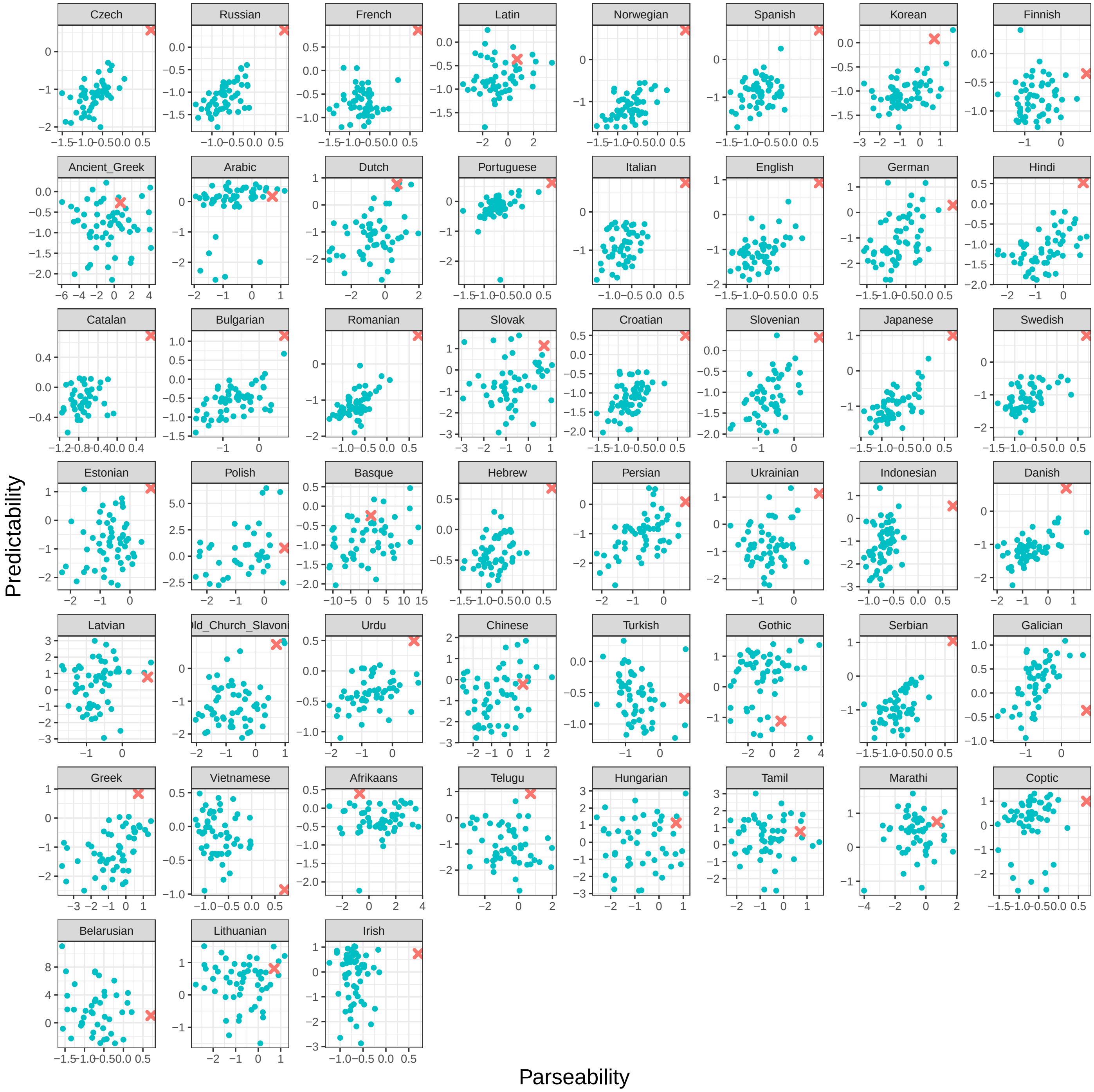

Figure S14: Study 1, replication with lexicalized models: Predictability and parseability of 51 languages, for *lexicalized* models, compare Figure S1.

Language	Pred. (t)	Parse. (t)	Pred. (Binomial)			Parseab. (Binomial)		
	p	p	Est.	CI	p	Est.	CI	p
Afrikaans	0.009 45	1	1	[0.95, 1]	$<2 \times 10^{-16}$	0.23	[0.14, 1]	1
Ancient Greek	2.4×10^{-10}	4.18×10^{-5}	0.84	[0.73, 1]	2.17×10^{-7}	0.75	[0.63, 1]	0.000 178
Arabic	0.0702	$<2 \times 10^{-16}$	0.56	[0.44, 1]	0.209	0.96	[0.89, 1]	4.28×10^{-14}
Basque	6.39×10^{-12}	0.0607	0.93	[0.84, 1]	1.02×10^{-11}	0.55	[0.43, 1]	0.295
Belarusian	0.0417	$<2 \times 10^{-16}$	0.56	[0.44, 1]	0.209	1	[0.95, 1]	$<2 \times 10^{-16}$
Bulgarian	$<2 \times 10^{-16}$	$<2 \times 10^{-16}$	1	[0.95, 1]	$<2 \times 10^{-16}$	1	[0.95, 1]	$<2 \times 10^{-16}$
Catalan	1.27×10^{-5}	$<2 \times 10^{-16}$	1	[0.95, 1]	$<2 \times 10^{-16}$	1	[0.95, 1]	$<2 \times 10^{-16}$
Chinese	0.000 172	2.14×10^{-13}	0.66	[0.54, 1]	0.0111	0.89	[0.8, 1]	5.09×10^{-10}
Coptic	4.06×10^{-6}	$<2 \times 10^{-16}$	0.85	[0.75, 1]	6.92×10^{-8}	1	[0.95, 1]	$<2 \times 10^{-16}$
Croatian	$<2 \times 10^{-16}$	$<2 \times 10^{-16}$	1	[0.95, 1]	$<2 \times 10^{-16}$	1	[0.95, 1]	$<2 \times 10^{-16}$
Czech	$<2 \times 10^{-16}$	$<2 \times 10^{-16}$	1	[0.94, 1]	2.22×10^{-16}	1	[0.94, 1]	8.88×10^{-16}
Danish	$<2 \times 10^{-16}$	$<2 \times 10^{-16}$	1	[0.95, 1]	$<2 \times 10^{-16}$	0.98	[0.91, 1]	6×10^{-15}
Dutch	$<2 \times 10^{-16}$	8.99×10^{-12}	0.98	[0.92, 1]	1.55×10^{-15}	0.85	[0.75, 1]	4.03×10^{-8}
English	$<2 \times 10^{-16}$	$<2 \times 10^{-16}$	1	[0.95, 1]	$<2 \times 10^{-16}$	1	[0.95, 1]	$<2 \times 10^{-16}$
Estonian	$<2 \times 10^{-16}$	$<2 \times 10^{-16}$	1	[0.95, 1]	$<2 \times 10^{-16}$	1	[0.95, 1]	$<2 \times 10^{-16}$
Finnish	3.92×10^{-13}	$<2 \times 10^{-16}$	0.92	[0.84, 1]	3.53×10^{-11}	1	[0.95, 1]	$<2 \times 10^{-16}$
French	7.81×10^{-8}	$<2 \times 10^{-16}$	1	[0.95, 1]	$<2 \times 10^{-16}$	1	[0.95, 1]	$<2 \times 10^{-16}$
Galician	0.343	$<2 \times 10^{-16}$	0.18	[0.1, 1]	1	1	[0.95, 1]	$<2 \times 10^{-16}$
German	8.14×10^{-16}	$<2 \times 10^{-16}$	0.93	[0.84, 1]	5.5×10^{-12}	1	[0.95, 1]	$<2 \times 10^{-16}$
Gothic	1	4.68×10^{-8}	0.07	[0.03, 1]	1	0.83	[0.73, 1]	3.64×10^{-7}
Greek	$<2 \times 10^{-16}$	1.49×10^{-11}	1	[0.95, 1]	$<2 \times 10^{-16}$	0.91	[0.82, 1]	1.95×10^{-10}
Hebrew	0.000 744	$<2 \times 10^{-16}$	1	[0.95, 1]	$<2 \times 10^{-16}$	1	[0.95, 1]	$<2 \times 10^{-16}$
Hindi	$<2 \times 10^{-16}$	$<2 \times 10^{-16}$	1	[0.95, 1]	$<2 \times 10^{-16}$	0.98	[0.91, 1]	6×10^{-15}
Hungarian	1.28×10^{-7}	5.52×10^{-14}	0.79	[0.68, 1]	1.12×10^{-5}	0.91	[0.81, 1]	3.54×10^{-10}
Indonesian	$<2 \times 10^{-16}$	$<2 \times 10^{-16}$	0.98	[0.92, 1]	1.55×10^{-15}	1	[0.95, 1]	$<2 \times 10^{-16}$
Irish	0.000 174	$<2 \times 10^{-16}$	0.85	[0.76, 1]	1.18×10^{-9}	1	[0.96, 1]	$<2 \times 10^{-16}$
Italian	9.09×10^{-11}	$<2 \times 10^{-16}$	1	[0.94, 1]	4.44×10^{-16}	1	[0.94, 1]	4.44×10^{-16}
Japanese	$<2 \times 10^{-16}$	$<2 \times 10^{-16}$	1	[0.95, 1]	$<2 \times 10^{-16}$	1	[0.95, 1]	$<2 \times 10^{-16}$
Korean	$<2 \times 10^{-16}$	1.82×10^{-15}	0.98	[0.92, 1]	1.55×10^{-15}	0.93	[0.84, 1]	1.02×10^{-11}
Latin	4.92×10^{-11}	2.99×10^{-9}	0.85	[0.75, 1]	6.92×10^{-8}	0.87	[0.76, 1]	3.49×10^{-8}
Latvian	0.0107	$<2 \times 10^{-16}$	0.52	[0.4, 1]	0.446	0.98	[0.92, 1]	1.55×10^{-15}
Lithuanian	5.64×10^{-5}	3.79×10^{-15}	0.75	[0.64, 1]	0.000 134	0.94	[0.86, 1]	2.76×10^{-12}
Marathi	1.07×10^{-5}	2.24×10^{-13}	0.74	[0.62, 1]	0.000 268	0.91	[0.81, 1]	3.54×10^{-10}
Norwegian	$<2 \times 10^{-16}$	$<2 \times 10^{-16}$	1	[0.94, 1]	2.22×10^{-16}	1	[0.94, 1]	2.22×10^{-16}
Old Church Slavonic	$<2 \times 10^{-16}$	$<2 \times 10^{-16}$	0.96	[0.89, 1]	2.22×10^{-14}	0.96	[0.89, 1]	2.22×10^{-14}
Persian	$<2 \times 10^{-16}$	$<2 \times 10^{-16}$	0.94	[0.86, 1]	2.76×10^{-12}	1	[0.95, 1]	$<2 \times 10^{-16}$
Polish	1.4×10^{-5}	$<2 \times 10^{-16}$	0.73	[0.61, 1]	0.000 508	1	[0.95, 1]	$<2 \times 10^{-16}$
Portuguese	0.0269	$<2 \times 10^{-16}$	1	[0.95, 1]	$<2 \times 10^{-16}$	1	[0.95, 1]	$<2 \times 10^{-16}$
Romanian	$<2 \times 10^{-16}$	$<2 \times 10^{-16}$	1	[0.95, 1]	$<2 \times 10^{-16}$	1	[0.95, 1]	$<2 \times 10^{-16}$
Russian	$<2 \times 10^{-16}$	$<2 \times 10^{-16}$	1	[0.95, 1]	$<2 \times 10^{-16}$	1	[0.95, 1]	$<2 \times 10^{-16}$
Serbian	$<2 \times 10^{-16}$	$<2 \times 10^{-16}$	1	[0.95, 1]	$<2 \times 10^{-16}$	1	[0.95, 1]	$<2 \times 10^{-16}$
Slovak	$<2 \times 10^{-16}$	1.85×10^{-15}	0.93	[0.84, 1]	1.9×10^{-11}	0.94	[0.86, 1]	1.46×10^{-12}
Slovenian	$<2 \times 10^{-16}$	$<2 \times 10^{-16}$	0.98	[0.91, 1]	1.18×10^{-14}	1	[0.95, 1]	$<2 \times 10^{-16}$
Spanish	1.87×10^{-12}	$<2 \times 10^{-16}$	1	[0.95, 1]	$<2 \times 10^{-16}$	1	[0.95, 1]	$<2 \times 10^{-16}$
Swedish	$<2 \times 10^{-16}$	$<2 \times 10^{-16}$	1	[0.95, 1]	$<2 \times 10^{-16}$	1	[0.95, 1]	$<2 \times 10^{-16}$
Tamil	0.0113	$<2 \times 10^{-16}$	0.58	[0.46, 1]	0.136	0.91	[0.82, 1]	1.95×10^{-10}
Telugu	$<2 \times 10^{-16}$	2.05×10^{-11}	1	[0.95, 1]	$<2 \times 10^{-16}$	0.89	[0.79, 1]	1.63×10^{-9}
Turkish	0.711	$<2 \times 10^{-16}$	0.47	[0.35, 1]	0.708	0.96	[0.89, 1]	1.59×10^{-13}
Ukrainian	$<2 \times 10^{-16}$	$<2 \times 10^{-16}$	0.98	[0.92, 1]	1.55×10^{-15}	1	[0.95, 1]	$<2 \times 10^{-16}$
Urdu	0.0205	$<2 \times 10^{-16}$	1	[0.94, 1]	4.44×10^{-16}	0.96	[0.88, 1]	3.06×10^{-13}
Vietnamese	1	$<2 \times 10^{-16}$	0.02	[0, 1]	1	1	[0.95, 1]	$<2 \times 10^{-16}$

Table S13: Study 1, replication with lexicalized models: Per-language results in Study 1, with *lexicalized* parsers and word-level-only language models. Compare Table S2

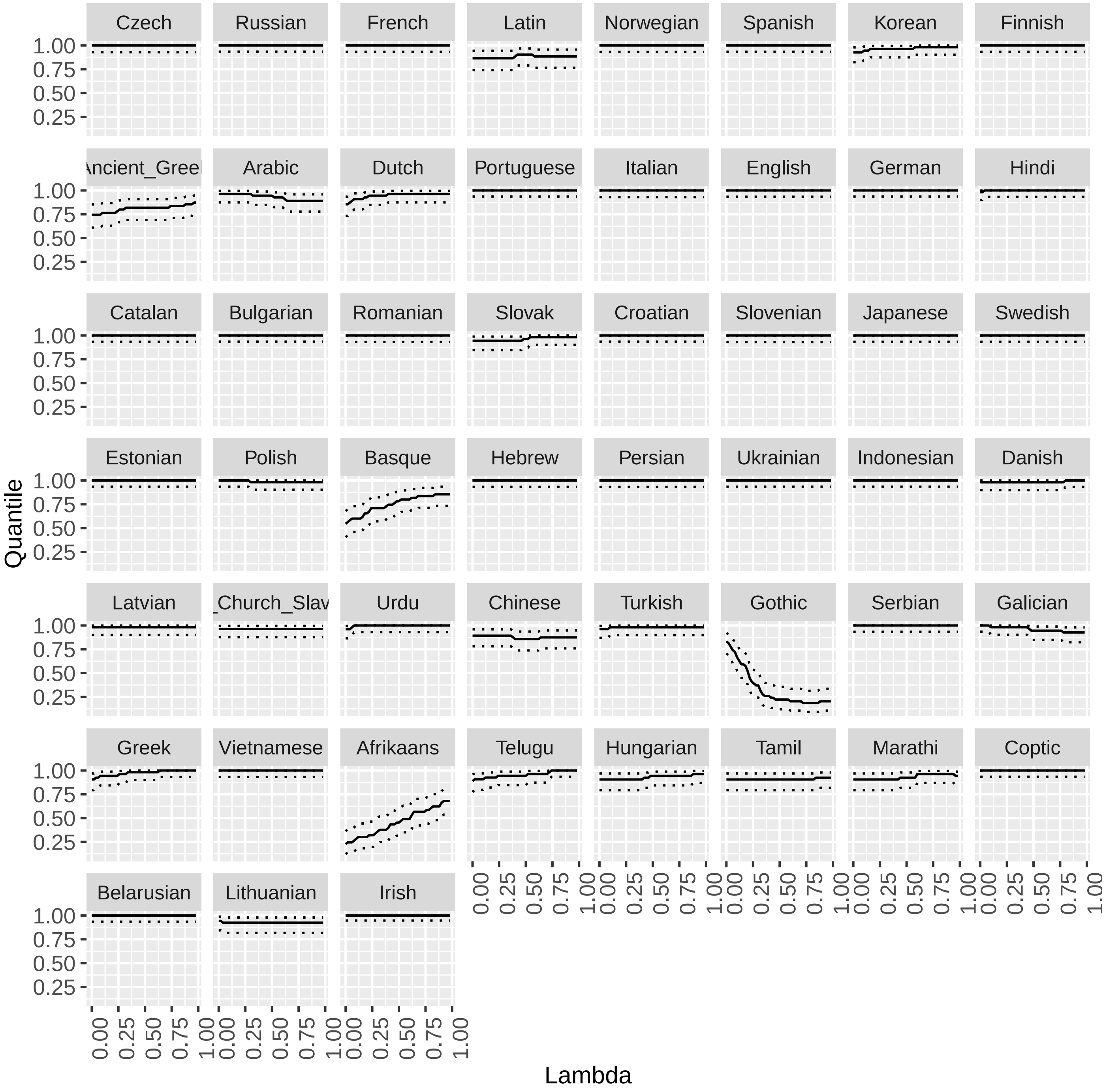

Figure S15: Study 1, replication with lexicalized models: Optimality of real grammars for efficiency, compared to baselines, across values of λ: The x-axis shows $\lambda \in [0, 1)$, the y-axis shows the fraction of baselines that have lower efficiency than the real grammar at this value of λ, with 95% confidence bands obtained from a two-sided binomial test. Compare Figure S3.

constructed a new set of 50 baseline grammars. Results are shown in Figures S16 and S17. The results agree with those found on the converted versions; across languages, real grammars are at the top-right of the baseline distributions, and (with the exception of Telugu, a language with a small corpus)

S8.3 Nondeterministic Baseline Grammars

In Study 1, we considered deterministic ordering grammars, and represented real languages using deterministic grammars extracted from observed orderings. This allowed us to ensure that we only compare baseline and real grammars that have exactly the same representational constraints, and utilize the same information encoded in the tree structures.

In this section, we consider baselines that allow word order freedom to degrees comparable to that found in orders observed in the actual corpus data. In order to obtain baselines whose freedom is comparable to that of real languages, we constructed baselines that have the same Branching Direction Entropy [88] as observed in the original corpora. The Branching Direction Entropy measures the extent of freedom in choice between head-final and head-initial orderings, and it is a corpus-based quantitative measure of word order freedom [88]. For a given syntactic relation, its branching direction entropy measures the entropy of the Bernoulli random variable that is 1 whenever the head is ordered before the dependent, and 0 if the dependent is ordered before the head. The branching direction entropy is 0 if only one of the two orders can occur, and it is $\log 2$ if both orders are equally frequent.

We constructed baseline grammars that match the branching direction entropies found in the original orders found in the corpora. To this end, we converted the baseline grammars into differentiable ordering grammars (Section S5.1). Such grammars have parameters a_τ, b_τ for each relation τ. For every one of the 37 syntactic relations, we chose a_τ so as to match the the direction entropy to that observed in the actual orderings found in the UD corpus. For b_τ, we considered the limit where the values b_τ for different relations τ are very far apart, making the relative ordering of siblings on the same side of the head fully deterministic. That is, these ordering grammars match word order freedom as quantified by Branching Direction Entropy, and show no additional degrees of order freedom.

Comparing deterministic and nondeterministic grammars Here, we compared nondeterministic baseline grammars to their deterministic versions, for one language with relatively free order (Czech), and for two languages with relatively fixed order (English and Japanese). Results are shown in Figure S18. For every one of the baseline grammars, we show both its deterministic and its nondeterministic version. Nondeterministic grammars are less efficient than deterministic grammars, in particular in languages with greater degrees of word order freedom (Czech). This shows that deterministic baseline grammars provide conservative baselines: They have higher efficiency than baseline grammars with word order freedom comparable to the orders found in the original corpora, and thus provide conservative baselines for comparison with other deterministic grammars.

Comparing observed orders to baselines with matched degree of nondeterminism Here, we compare the efficiency of the orders observed in the corpora with baselines whose degree of nondeterminism, quantified by branching direction entropy, is matched to that of the observed orders. We show results in Figures S19 and S20. Figure S19 shows that observed orders are mostly to the top and/or right of baselines with matched degree of nondetermminism. Figure S20 shows that, with the exception of Telugu (a language with a small corpus), the observed orders have higher efficiency than most baselines at least for some values of λ.

S9 Effects of data sparsity

Here, we investigate whether the difference between real and baseline grammars is affected by the size of available datasets. We are addressing the following confound: It is conceivable that with enough data, our neural network language models and parsers would do equally well on real grammars and baseline grammars. If the difference between random and real grammars is due to data sparsity in this way, then we expect that the difference will decrease as the amount of training data is increased. If, on the other hand, there is an inherent difference in efficiency between random and real grammars, we expect that the difference will persist as training data is increased.

We considered Czech, the UD language with the largest amount of available treebank data (approx. 2.2 million words), up to $\approx$ 300 times more data than is available for some other UD languages. We considered both a random ordering grammar, and the best ordering grammar optimized for parseabaility. For both of these ordering grammars, we trained the parser on successively larger portions of the training data (0.1 %, 1 %, 5%, 10%, 20 %, ..., 90 %, 100 %) and recorded parsing accuracy. Furthermore, for the random grammar, we varied the number of neurons in the BiLSTM (200, 400, 800) to test whether results depend on the capacity of the network.

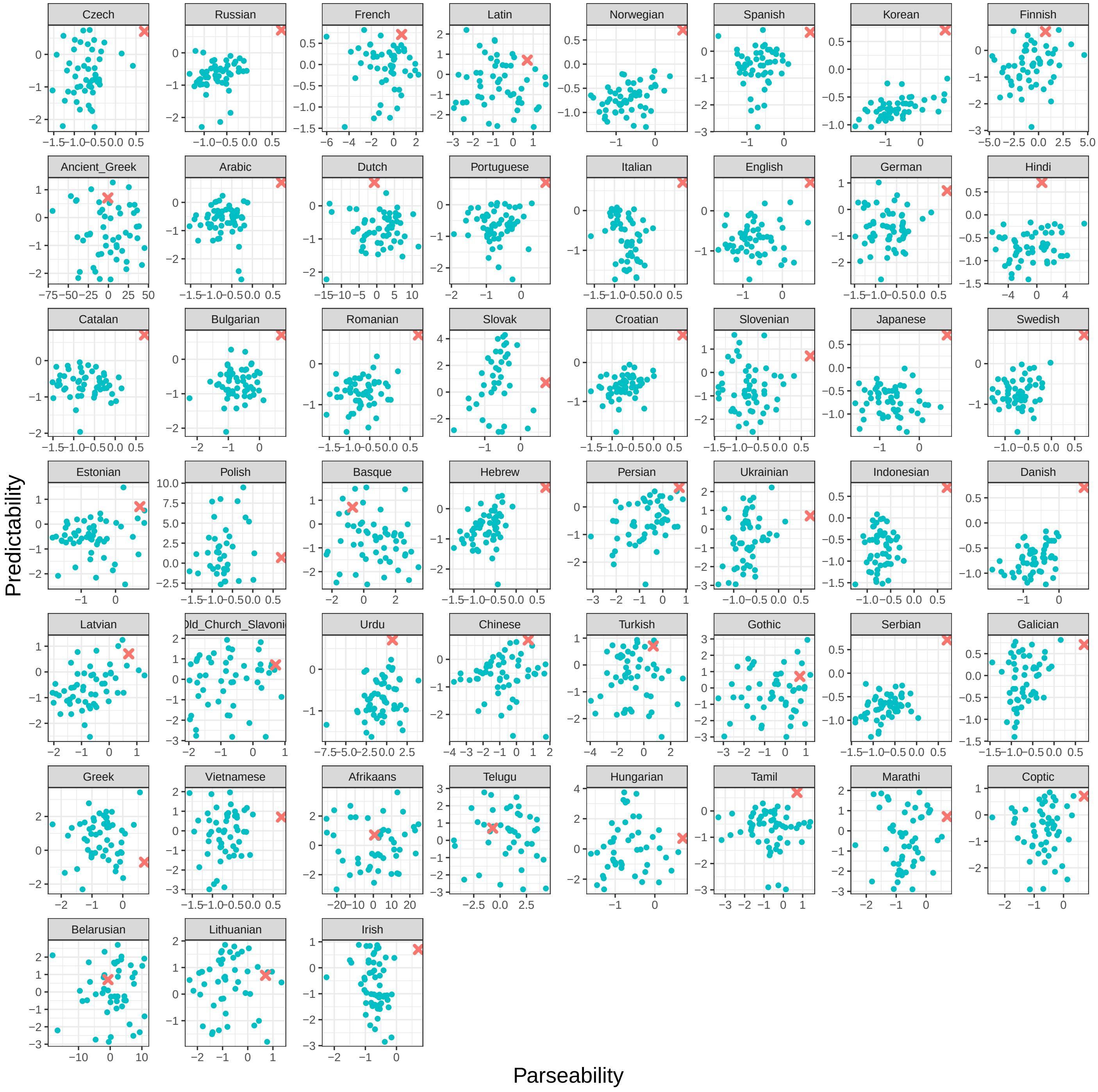

Figure S16: Study 1, replication with the original UD format: Predictability and parseability of real and baseline grammars in 51 languages, compare Figure S1.

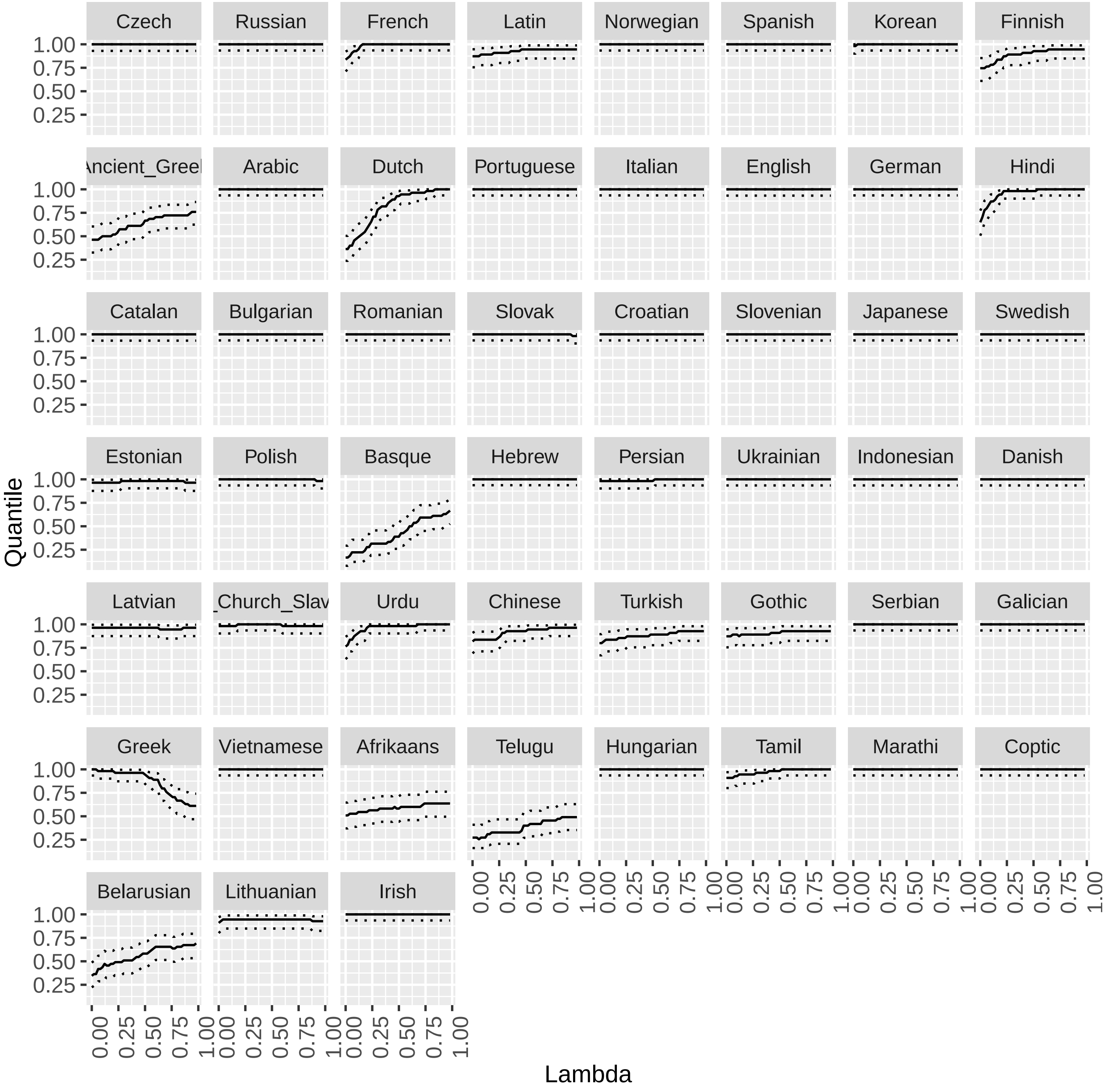

Figure S17: Study 1, replication with the original UD format: Optimality of real grammars for efficiency, compared to baselines, across values of λ: The x-axis shows $\lambda \in [0, 1)$, the y-axis shows the fraction of baselines that have lower efficiency than the real grammar at this value of λ, with 95% confidence bands obtained from a two-sided binomial test. Compare Figure S3.

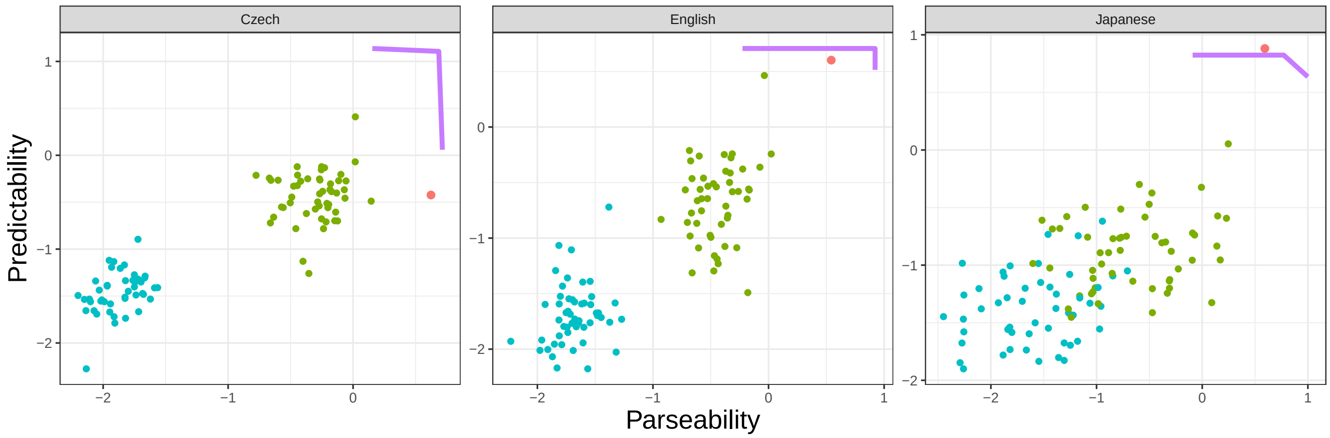

Figure S18: Parseability and predictability for three languages, including both deterministic (green, light) and nondeterministic (blue, dark) versions of the 50 baseline grammars.

The resulting curves are shown in Figure S21. A gap in parsing loss of about 0.2 nats appears already at 0.01 % of the training data (2000 words), and persists for larger amounts of training data. This shows that the observed efficiency differences between grammars cannot be attributed to data sparsity.

S10 Languages and Corpus Sizes

In Table S14, we list the 51 languages with ISO codes and families, with the size of the available data per language. We included all UD 2.1 languages for which a training partition was available.

S11 Dependency Length Minimization

Prior work has suggested *Dependency Length Minimization* (DLM) as a characteristic of efficient word order [5, 89, 90, 91]. This is the idea that word order minimizes the average distance between syntactically related words. It is known that human languages reduce dependency length compared to random baselines [92, 5, 90, 91]. Prior work has suggested principles akin to DLM as approximating efficiency optimization of grammars [93, 94, 30, 95]. It is a heuristic formalization of the idea that long dependencies should create high memory requirements in parsing and prediction [93, 96, 97, 30]. Indeed, [30] argues specifically that it emerges from efficiency optimization.

Dependency length is typically quantified as the average distance between all pairs of syntactically related words, measured by the number of intervening words [92, 5]. Dependency length quantified in this manner is a heuristic measure of complexity: The actual empirically-measured processing complexity induced by long dependencies is not a linear function of length and depends crucially on the types of dependencies involved [98] and the specific elements intervening between the head and dependent [96, 97, 99].

We asked whether efficiency optimization predicts dependency length minimization effects. We first computed dependency length for grammars optimized for efficiency. We found that 100% of grammars optimized for efficiency reduce average dependency length compared to baseline grammars ($p < 0.05$, by one-sided t-test). This suggests that the reduction of dependency length observed in natural language is indeed predicted by efficiency maximization, confirming theoretical arguments made in prior work [93, 94, 30, 95]. Next, we constructed grammars that minimize average dependency length, using the same gradient descent method as we used for efficiency optimization (Section S5.3). We expect that such grammars should have shorter dependency length than the real grammars, or grammars optimized for efficiency. In Figure S22, we plot the mean dependency length for optimized, real, and baseline orderings.[14] We find that optimizing grammars for efficiency reduces dependency length to a similar degree as found in the actual orderings in the corpora, almost up to the limit given by directly optimizing for dependency length. We also plot more detailed results for four languages in Figure S23, plotting dependency length as a function of sentence length as reported in prior work [100, 5]. Optiziming grammars for efficiency produces dependency lengths similar to those found in the actual orderings.

[14]We show results for the actually observed orderings, not for corpora ordered according to extracted grammars as in Study 1; results are similar for those extracted grammars.

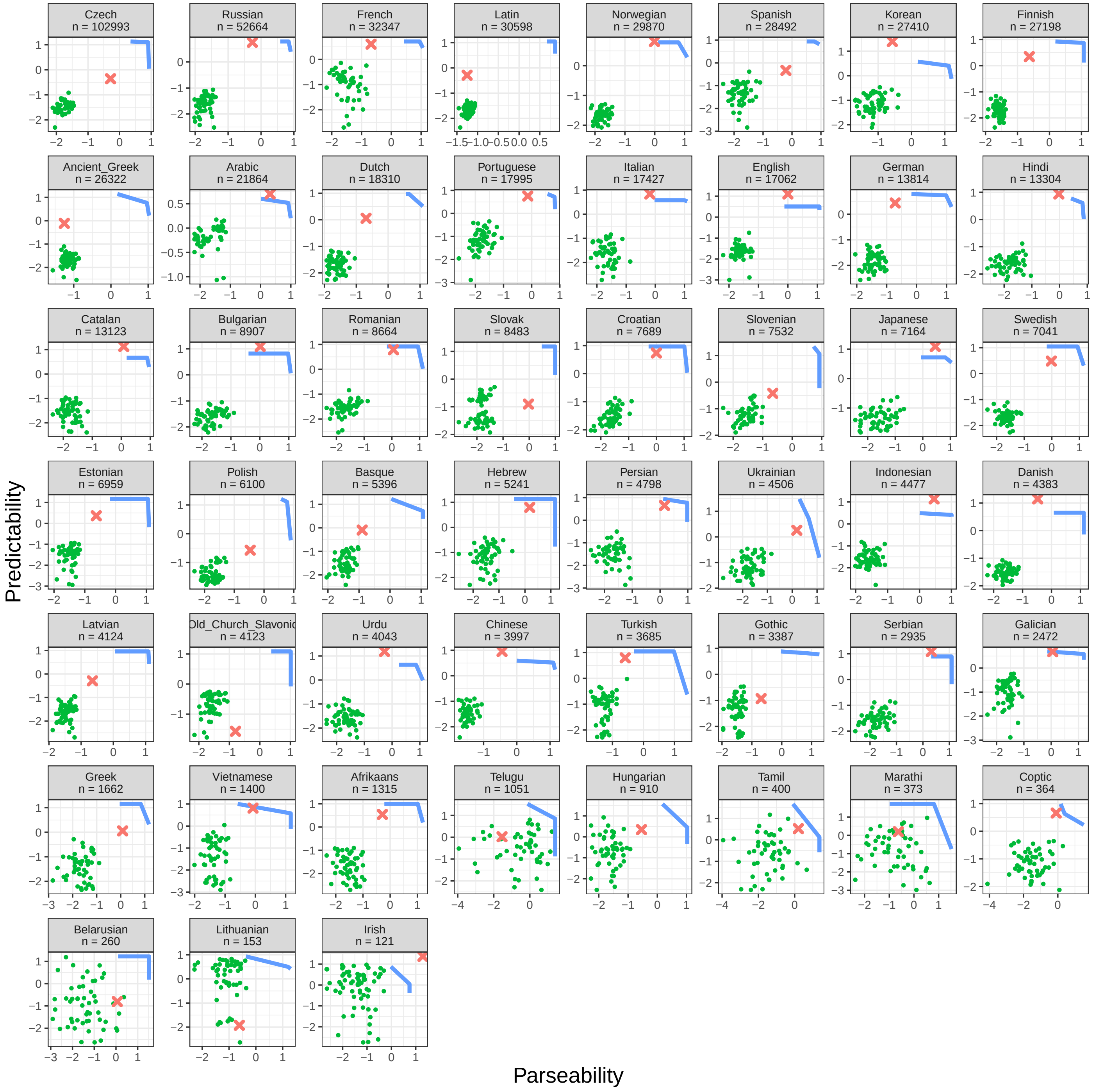

Figure S19: Comparing observed orders (red crosses) with baselines (green) whose degree of nondeterminism is matched to the observed order. Compare Figure S1.

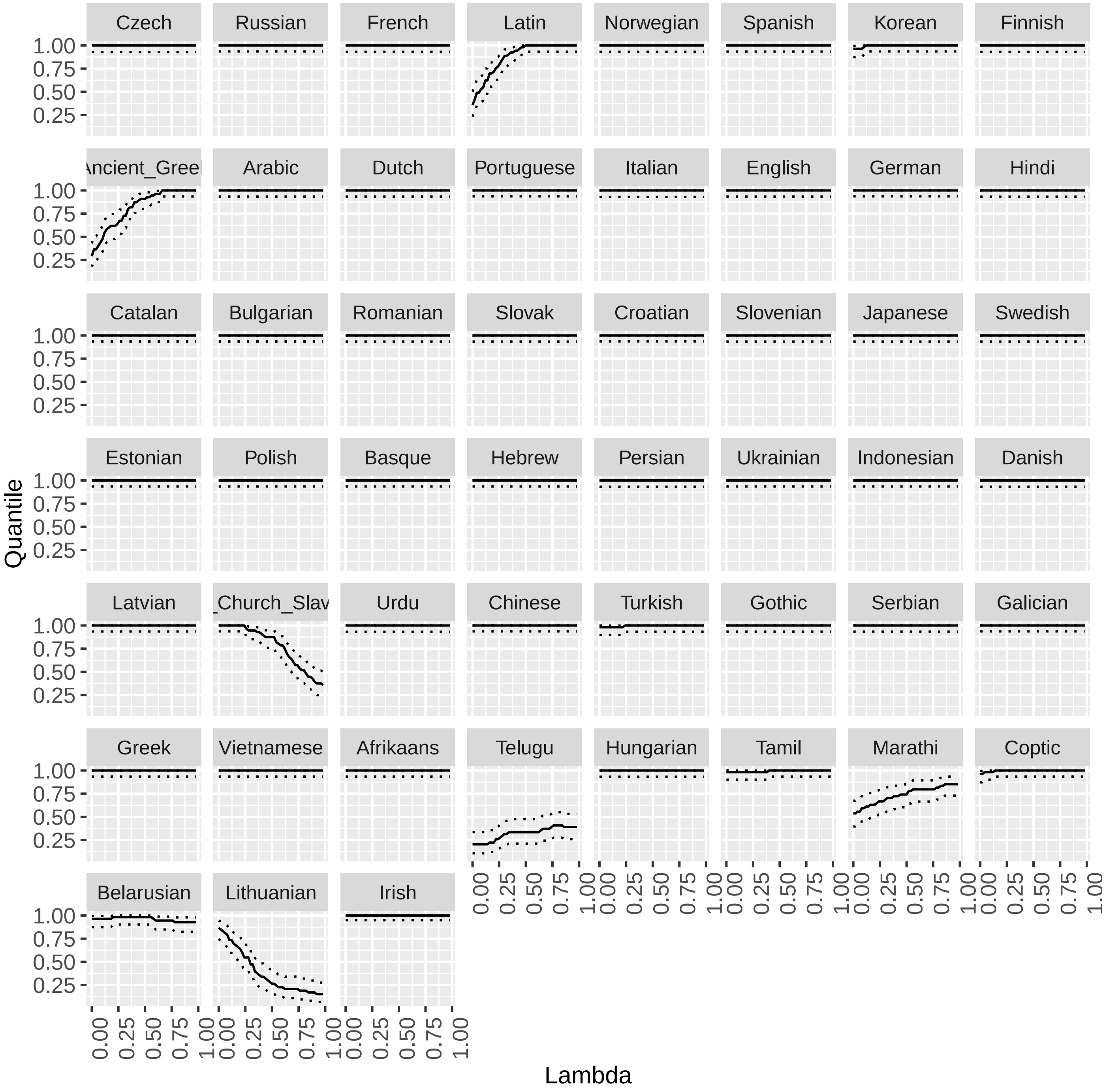

Figure S20: Optimality of observed orders for efficiency, compared to nondeterministic baselines, across values of λ: The x-axis shows $\lambda \in [0, 1)$, the y-axis shows the fraction of baselines that have lower efficiency than the observed orders at this value of λ, with 95% confidence bands obtained from a two-sided binomial test. Compare Figure S3.

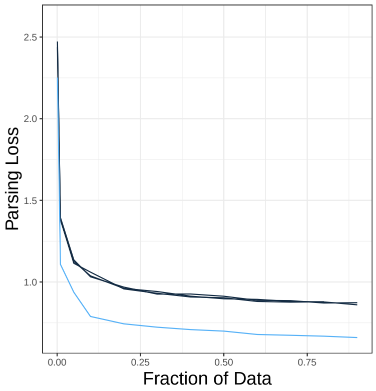

Figure S21: Parsing loss (H[$\mathcal{T}|\mathcal{U}$], normalized by sentence length) for optimized (light blue) and random (black) ordering grammar on Czech data, as a function of the fraction of total training data provided.

Next, we examined the word order properties of grammars optimized for DLM. In Table S15, we report the posterior prevalence of word order correlations in grammars optimized for DLM; our results show that optimizing for DLM makes predictions similar to efficiency optimization. We find that these grammars also exhibit the eight correlations, similar to grammars directly optimized for efficiency. This is itself a novel result, suggesting that it is in part through favoring short dependencies that efficiency predicts word order universals, an idea that has been proposed in prior theoretical studies, though never tested computationally on large-scale text data [101, 102, 103, 104, 93, 94]. On other correlations, predictions of DLM also resemble those of efficiency optimization. However, it predicts strong correlations with *amod* (adjectival modifiers) and *nummod* (numeral modifiers) (see bottom of Table S15), which are not borne out typologically. In these cases, efficiency optimization predicts prevalences closer to 50%, in line with typological data.

In conclusion, these results suggest that the phenomenon of dependency length minimization is a by-product of efficiency optimization, providing support to theoretical arguments from the linguistic literature [93, 30, 95]. Furthermore, optimizing for dependency length correctly predicts a range of word order facts, though it appears to *overpredict* correlations when compared to direct optimization for communicative efficiency.

S12 Efficiency and correlating orders in toy grammars

When we optimize grammars for efficiency, we find that the optimized grammars exhibit dependency length minimization and the Greenbergian word order correlations. To some extent, this result is surprising, because previous functional explanations for DLM (and the Greenbergian correlations, which have been argued to arise from DLM) have been based on the idea of limitations in working memory, and yet our models do not instantiate any explicit working memory pressures; see also Section S7.2 above for evidence against the idea that a locality bias arises from our parsers. Our results therefore suggest that DLM and word order correlations might arise purely because they enable tree structures to be better recovered from trees, and/or they make sequences more predictable.

Here we perform some simulation studies to bolster the argument that DLM and word order correlations can enhance the recoverability of tree structures in a generic sense, without any appeal to memory limitations. To do so, we experiment with toy grammars that can be defined to either (1) exhibit word order correlations or (2) not, and we test whether the grammars of type (1) are more or less parseable than the grammars of type (2). We measure parseability using a CYK PCFG parser, thus removing any potential confounds arising from the neural network parsing model.

Our toy grammar consists of the following head-outward generative model [105]. Verbs generate verb dependents (*xcomp*) and noun dependents (*obj*), independently. The overall number N of dependents is $NB(1, p_{\text{branching}})$, the number of *obj* dependents is $Binom(p_{obj}, N)$. Nouns can generate verb dependents (*acl*), of number $NB(1, p_{\text{acl}})$.

Trees are linearized using one of two grammars: One (‘Correlating’) places *obj*, *xcomp*, and *acl* dependents on the same side of the head, and (in accordance with crosslinguistic tendencies) places the *obj* dependents closer to the head than *xcomp* dependents. The other grammar (‘Anti-Correlating’) places *xcomp* and *acl* dependents opposite to *obj* dependents.

An example is provided in Figure S24. We show how the two grammars linearize the same syntactic dependency structure: The correlating grammar (left) linearizes the three relation types towards the right of the head; the anti-correlating one places *obj* dependencies on the left and the other dependencies on the right. This example provides some intuitive idea of why the correlating grammar might lead to improved parseability: Note that the red boldface token labeled ‘N’ occupies the same structural position in both versions. In the anti-correlating version (right), when given only

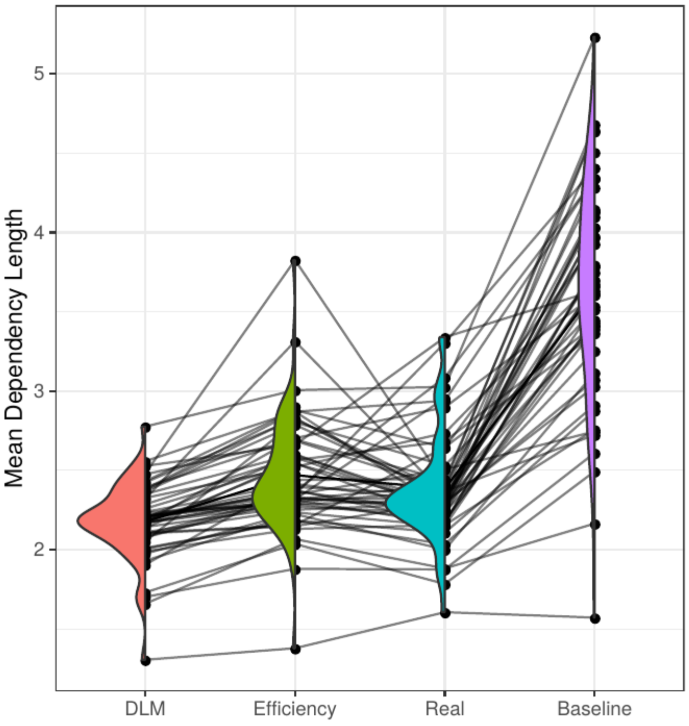

Figure S22: Average dependency length for grammars optimized to minimize dependency length (DLM, left), optimized for efficiency (second), the real orderings found in corpora (third), and random baseline grammars (right). The lines connect the mean points for each of the 51 languages in our sample.

the token sequence, without the syntactic structure, this word could a priori be an *obj* dependent of any of the three verbs occurring to its right. In the correlating version (left), this 'N' token can only possibly be a dependent of the verb occurring to its left.

In order to test this intuition on the level of the entire tree distribution, we formulated this model as a binary-branching PCFG, and used a CKY parser to estimate $I[\mathcal{T},\mathcal{U}]$ from 10,000 random sample sentences.

We computed this for different settings of $p_{\text{branching}} \in [0, 0.5]$ and $p_{obj} \in [0, 1]$, at $p_{\text{acl}} \in \{0, 0.3\}$.[15] For these settings, we computed the difference in $I[\mathcal{T},\mathcal{U}]$ between the two grammars.

Results are shown in Figure S25. For almost all parameter regimes, the correlating grammars have better parseability than the anti-correlating grammars. This is especially the case for grammars with high $p_{\text{branching}}$.

This simulation shows that the Greenbergian word order correlations can in principle improve parseability in the controlled setting of such a model, without any appeal to memory limitations; we leave a full graph-theoretical understanding of this phenomenon to future work.

References

[1] Matthew S Dryer. The Greenbergian word order correlations. *Language*, 68(1):81–138, 1992.

[2] M. Dunn, S.J. Greenhill, S.C. Levinson, and R.D. Gray. Evolved structure of language shows lineage-specific trends in word-order universals. *Nature*, 473(7345):79–82, 2011.

[3] Roger Levy and Hal Daumé. Computational methods are invaluable for typology, but the models must match the questions. *Linguistic Typology*, 15(2):393–399, 2011.

[4] William Croft, Tanmoy Bhattacharya, Dave Kleinschmidt, D Eric Smith, and T Florian Jaeger. Greenbergian universals, diachrony, and statistical analyses. *Linguistic Typology*, 15(2):433–453, 2011.

[15] For $p_{\text{acl}} = 0.3$, we only computed values for $p_{\text{branching}} \leq 0.4$, due to high computational cost on long sentences resulting from $p_{\text{branching}} = 0.5$ and $p_{\text{acl}} = 0.3$.

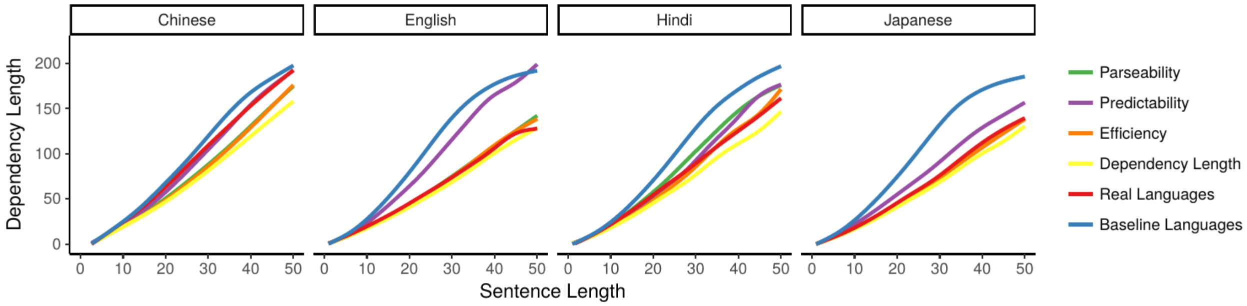

Figure S23: Total dependency length as a function of sentence length, for four diverse languages. We show results for optimized grammars (parseability, predictability, efficiency), for grammars specifically optimized to minimize dependency length, of the actual real orderings, and of the baseline grammars.

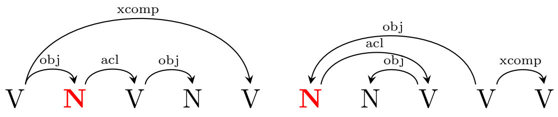

Figure S24: Linearizations of a syntactic dependency structure, under a correlating grammar (left) and an anti-correlating grammar (right).

[5] Richard Futrell, Kyle Mahowald, and Edward Gibson. Large-scale evidence of dependency length minimization in 37 languages. *Proceedings of the National Academy of Sciences*, 112(33):10336–10341, 2015. doi: 10.1073/pnas.1502134112. URL `http://www.pnas.org/content/early/2015/07/28/1502134112.abstract`.

[6] Joseph H. Greenberg. Some universals of grammar with particular reference to the order of meaningful elements. In Joseph H. Greenberg, editor, *Universals of Language*, pages 73–113. MIT Press, Cambridge, MA, 1963.

[7] Matthew S. Dryer. Order of subject, object and verb. In Matthew S. Dryer and Martin Haspelmath, editors, *The World Atlas of Language Structures Online*. Max Planck Institute for Evolutionary Anthropology, Leipzig, 2013. URL `https://wals.info/chapter/81`.

[8] Martin Haspelmath. Against markedness (and what to replace it with). *Journal of linguistics*, 42(1):25–70, 2006.

[9] William A. Croft. *Typology and universals.* Cambridge Textbooks in Linguistics. Cambridge University Press, Cambridge, 2nd edition, 2003.

[10] G. Cinque. Deriving Greenberg's Universal 20 and its exceptions. *Linguistic inquiry*, 36(3):315–332, 2005.

[11] Jennifer Culbertson and David Adger. Language learners privilege structured meaning over surface frequency. *Proceedings of the National Academy of Sciences*, 111(16):5842–5847, 2014. ISSN 0027-8424. doi: 10.1073/pnas.1320525111. URL `http://www.pnas.org/content/111/16/5842`.

[12] Matthew S Dryer. On the order of demonstrative, numeral, adjective, and noun. *Language*, 94(4):798–833, 2018.

[13] Frans Plank and Elena Filimonova. The universals archive: A brief introduction for prospective users. *STUF-Language Typology and Universals*, 53(1):109–123, 2000.

[14] John Mace. *Persian Grammar: For reference and revision.* Routledge, 2015.

[15] Annie Montaut. A grammar of hindi (lincom studies in indo-european linguistics). *Munich: LINCOM GmbH*, 2004.

[16] Fred Karlsson. *Finnish: An essential grammar.* Routledge, 2013.

[17] Valter Tauli. Standard estonian grammar. part ii syntax. *Acta Universitatis Upsaliensis. Studia Uralica et Altaica Upsaliensia*, 14, 1983.

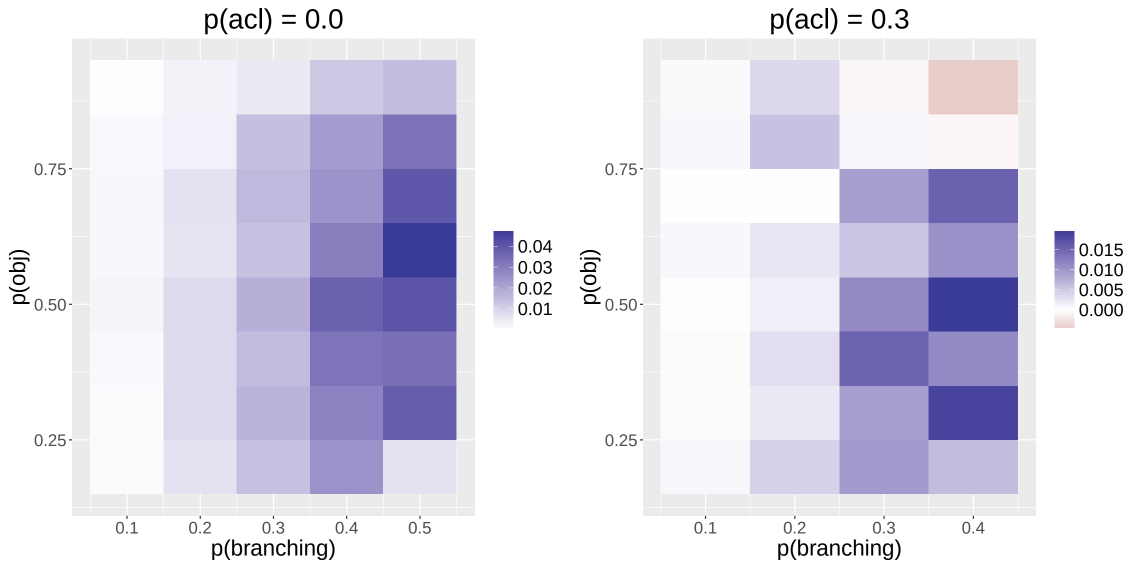

Figure S25: Difference in parseability $I[\mathcal{T},\mathcal{U}]$ (normalized by sentence length) between the correlating and anti-correlating grammars, for $p_{\text{acl}} = 0.0$ (left) and $p_{\text{acl}} = 0.3$ (right). Positive values indicate greater values of $I[\mathcal{T},\mathcal{U}]$ for correlating grammars, i.e. cases where the grammars that exhibit natural-language-like correlations are more parseable than grammars that do not.

[18] Daniel Zeman, David Marecek, Martin Popel, Loganathan Ramasamy, Jan Stepánek, Zdenek Zabokrtskỳ, and Jan Hajic. HamleDT: To parse or not to parse? In *Proceedings of the 8th International Conference on Language Resources and Evaluation*, pages 2735–2741, 2012.

[19] Loganathan Ramasamy and Zdeněk Žabokrtský. Tamil dependency parsing: results using rule based and corpus based approaches. In *Proceedings of the 12th international conference on Computational linguistics and intelligent text processing - Volume Part I*, CICLing'11, pages 82–95, Berlin, Heidelberg, 2011. Springer-Verlag. ISBN 978-3-642-19399-6. URL `http://portal.acm.org/citation.cfm?id=1964799.1964808`.

[20] Ramon Ferrer i Cancho and Ricard V Solé. Least effort and the origins of scaling in human language. *Proceedings of the National Academy of Sciences*, 100(3):788–791, 2003.

[21] Ramon Ferrer i Cancho and Albert Díaz-Guilera. The global minima of the communicative energy of natural communication systems. *Journal of Statistical Mechanics: Theory and Experiment*, 2007(06):P06009, 2007.

[22] Charles Kemp and Terry Regier. Kinship categories across languages reflect general communicative principles. *Science*, 336(6084):1049–1054, 2012.

[23] Michael C Frank and Noah D Goodman. Predicting pragmatic reasoning in language games. *Science*, 336(6084): 998–998, 2012.

[24] Noah D. Goodman and Andreas Stuhlmüller. Knowledge and implicature: Modeling language understanding as social cognition. *Topics in Cognitive Science*, 5(1):173–184, 2013. doi: 10.1111/tops.12007.

[25] Justine T. Kao, Jean Y. Wu, Leon Bergen, and Noah D. Goodman. Nonliteral understanding of number words. *Proceedings of the National Academy of Sciences*, 111(33):12002–12007, 2014. doi: 10.1073/pnas.1407479111.

[26] Yang Xu and Terry Regier. Numeral systems across languages support efficient communication: From approximate numerosity to recursion. In P. Bello, M. Guarini, M. McShane, and B. Scassellati, editors, *Proceedings of the 36th annual meeting of the Cognitive Science Society*, pages 1802–1807, Austin, TX, 2014. Cognitive Science Society.

[27] Simon Kirby, Monica Tamariz, Hannah Cornish, and Kenny Smith. Compression and communication in the cultural evolution of linguistic structure. *Cognition*, 141:87–102, 2015.

[28] Terry Regier, Charles Kemp, and Paul Kay. Word meanings across languages support efficient communication. In *The Handbook of Language Emergence*, pages 237–263. Wiley-Blackwell, Hoboken, NJ, 2015.

[29] Yang Xu, Terry Regier, and Barbara C. Malt. Historical semantic chaining and efficient communication: The case of container names. *Cognitive Science*, 40:2081–2094, 2016.

[30] Richard Futrell. *Memory and locality in natural language*. PhD thesis, Massachusetts Institute of Technology, Cambridge, MA, 2017.

[31] Noga Zaslavsky, Charles Kemp, Terry Regier, and Naftali Tishby. Efficient compression in color naming and its evolution. *Proceedings of the National Academy of Sciences*, 115(31):7937–7942, 2018.

[32] Erin D Bennett and Noah D Goodman. Extremely costly intensifiers are stronger than quite costly ones. *Cognition*, 178:147–161, 2018.

[33] Michael Hahn, Judith Degen, Noah Goodman, Dan Jurafsky, and Richard Futrell. An information-theoretic explanation of adjective ordering preferences. In *Proceedings of the 40th Annual Meeting of the Cognitive Science Society (CogSci)*, 2018.

[34] Benjamin Peloquin, Noah Goodman, and Mike Frank. The interactions of rational, pragmatic agents lead to efficient language structure and use. In *CogSci*, 2019.

[35] Noga Zaslavsky, Terry Regier, Naftali Tishby, and Charles Kemp. Semantic categories of artifacts and animals reflect efficient coding. In *CogSci*, 2019.

[36] Gottlob Frege. Über sinn und bedeutung. *Zeitschrift für Philosophie und philosophische Kritik*, 100(1):25–50, 1892.

[37] John T. Hale. A probabilistic Earley parser as a psycholinguistic model. In *Proceedings of the Second Meeting of the North American Chapter of the Association for Computational Linguistics and Language Technologies*, pages 1–8, 2001.

[38] Roger Levy. Expectation-based syntactic comprehension. *Cognition*, 106(3):1126–1177, 2008.

[39] Nathaniel J. Smith and Roger Levy. The effect of word predictability on reading time is logarithmic. *Cognition*, 128 (3):302–319, 2013.

[40] Naftali Tishby, Fernando Pereira, and William Bialek. The information bottleneck method. In *Invited paper to The 37th annual Allerton Conference on Communication, Control, and Computing*, 1999.

[41] DJ Strouse and David J Schwab. The deterministic information bottleneck. *Neural computation*, 29(6):1611–1630, 2017.

[42] Terry Regier, Paul Kay, and Naveen Khetarpal. Color naming reflects optimal partitions of color space. *Proceedings of the National Academy of Sciences*, 104:1436–1441, 2007.

[43] Toby Berger. *Rate Distortion Theory, A Mathematical Basis for Data Compression.* Prentice Hall, 1971.

[44] Ralph Linsker. Self-organization in a perceptual network. *Computer*, 21(3):105–117, 1988.

[45] Karl Friston. The free-energy principle: A unified brain theory? *Nature Reviews Neuroscience*, 11(2):127, 2010.

[46] Jim W Kay and WA Phillips. Coherent infomax as a computational goal for neural systems. *Bulletin of mathematical biology*, 73(2):344–372, 2011.

[47] Timothy Dozat, Peng Qi, and Christopher D Manning. Stanford's graph-based neural dependency parser at the conll 2017 shared task. *Proceedings of the CoNLL 2017 Shared Task: Multilingual Parsing from Raw Text to Universal Dependencies*, pages 20–30, 2017.

[48] Daniel Zeman and Jan Hajič, editors. *Proceedings of the CoNLL 2018 Shared Task: Multilingual Parsing from Raw Text to Universal Dependencies.* Association for Computational Linguistics, Brussels, Belgium, October 2018. URL `http://www.aclweb.org/anthology/K18-2`.

[49] Wanxiang Che, Yijia Liu, Yuxuan Wang, Bo Zheng, and Ting Liu. Towards better UD parsing: Deep contextualized word embeddings, ensemble, and treebank concatenation. In *Proceedings of the CoNLL 2018 Shared Task: Multilingual Parsing from Raw Text to Universal Dependencies*, pages 55–64, Brussels, Belgium, October 2018. Association for Computational Linguistics. URL `http://www.aclweb.org/anthology/K18-2005`.

[50] Sebastian J. Mielke, Ryan Cotterell, Kyle Gorman, Brian Roark, and Jason Eisner. What kind of language is hard to language-model? In *Proceedings of the 2019 Conference of the Association for Computational Linguistics (ACL)*, Florence, 2019. URL http://cs.jhu.edu/~jason/papers/#mielke-et-al-2019.

[51] Andrew Gelman, Hal S Stern, John B Carlin, David B Dunson, Aki Vehtari, and Donald B Rubin. *Bayesian data analysis*. Chapman and Hall/CRC, 2013.

[52] Joyee Ghosh, Yingbo Li, Robin Mitra, et al. On the use of cauchy prior distributions for bayesian logistic regression. *Bayesian Analysis*, 13(2):359–383, 2018.

[53] Paul-Christian Bürkner. Advanced bayesian multilevel modeling with the r package brms. *The R Journal*, 10(1): 395–411, 2018.

[54] Bob Carpenter, Andrew Gelman, Matthew D Hoffman, Daniel Lee, Ben Goodrich, Michael Betancourt, Marcus Brubaker, Jiqiang Guo, Peter Li, and Allen Riddell. Stan: A probabilistic programming language. *Journal of statistical software*, 76(1), 2017.

[55] Matthew D Hoffman and Andrew Gelman. The no-u-turn sampler: adaptively setting path lengths in hamiltonian monte carlo. *Journal of Machine Learning Research*, 15(1):1593–1623, 2014.

[56] Paul-Christian Bürkner. brms: An r package for bayesian multilevel models using stan. *Journal of Statistical Software, Articles*, 80(1):1–28, 2017.

[57] Douglas M. Bates, Martin Mächler, Ben Bolker, and Steve Walker. Fitting linear mixed-effects models using lme4. *Journal of Statistical Software*, 67(1):1–48, 2015. doi: 10.18637/jss.v067.i01.

[58] John S. Justeson and Laurence D. Stephens. Explanation for word order universals: a log-linear analysis. In *Proceedings of the XIV International Congress of Linguists*, Berlin, 1990. Mouton de Gruyter.

[59] Daniel Gildea and T. Florian Jaeger. Human languages order information efficiently. *arXiv*, 1510.02823, 2015. URL http://arxiv.org/abs/1510.02823.

[60] Ian Goodfellow, Yoshua Bengio, and Aaron Courville. *Deep Learning*. MIT Press, 2016. http://www.deeplearningbook.org.

[61] Richard Futrell and Edward Gibson. Experiments with generative models for dependency tree linearization. In *Proceedings of the 2015 Conference on Empirical Methods in Natural Language Processing*, pages 1978–1983, Lisbon, Portugal, September 2015. Association for Computational Linguistics. URL http://aclweb.org/anthology/D15-1231.

[62] Dingquan Wang and Jason Eisner. The Galactic Dependencies Treebanks: Getting more data by synthesizing new languages. *Transactions of the Association for Computational Linguistics*, 4:491–505, 2016.

[63] Eli Bingham, Jonathan P. Chen, Martin Jankowiak, Fritz Obermeyer, Neeraj Pradhan, Theofanis Karaletsos, Rohit Singh, Paul Szerlip, Paul Horsfall, and Noah D. Goodman. Pyro: Deep Universal Probabilistic Programming. *Journal of Machine Learning Research*, 2018.

[64] M. Haspelmath, M.S. Dryer, D. Gil, and B.. Comrie. The World Atlas of Language Structures Online. 2005.

[65] Matthew S Dryer. The evidence for word order correlations. *Linguistic Typology*, 15(2):335–380, 2011.

[66] Thomas M. Cover and J.A. Thomas. *Elements of Information Theory*. John Wiley & Sons, Hoboken, NJ, 2006.

[67] Ronald J. Williams. Simple statistical gradient-following algorithms for connectionist reinforcement learning. *Machine learning*, 8(3-4):229–256, 1992.

[68] Yoav Goldberg. Neural network methods for natural language processing. *Synthesis Lectures on Human Language Technologies*, 10(1):1–309, 2017.

[69] Sepp Hochreiter and Jürgen Schmidhuber. Long short-term memory. *Neural Computation*, 9(8):1735–1780, 1997.

[70] Stefan L. Frank and Rens Bod. Insensitivity of the human sentence-processing system to hierarchical structure. *Psychological Science*, 22(6):829–834, 2011.

[71] Adam Goodkind and Klinton Bicknell. Predictive power of word surprisal for reading times is a linear function of language model quality. In *Proceedings of the 8th Workshop on Cognitive Modeling and Computational Linguistics (CMCL 2018)*, pages 10–18, Salt Lake City, UT, 2018. Association for Computational Linguistics.

[72] Eliyahu Kiperwasser and Yoav Goldberg. Simple and accurate dependency parsing using bidirectional lstm feature representations. *Transactions of the Association of Computational Linguistics*, 4:313–327, 2016.

[73] Xingxing Zhang, Jianpeng Cheng, and Mirella Lapata. Dependency parsing as head selection. In *Proceedings of the 15th Conference of the European Chapter of the Association for Computational Linguistics: Volume 1, Long Papers*, pages 665–676, Valencia, Spain, 2017.

[74] Ryan T McDonald, Fernando Pereira, Kiril Ribarov, and Jan Hajič. Non-projective dependency parsing using spanning tree algorithms. In *Proceedings of the Conference on Human Language Technology and Empirical Methods in Natural Language Processing*, pages 523–530. Association for Computational Linguistics, 2005.

[75] Matthew E Peters, Mark Neumann, Mohit Iyyer, Matt Gardner, Christopher Clark, Kenton Lee, and Luke Zettlemoyer. Deep contextualized word representations. *arXiv preprint arXiv:1802.05365*, 2018.

[76] John Schulman, Nicolas Heess, Theophane Weber, and Pieter Abbeel. Gradient estimation using stochastic computation graphs. In *Advances in Neural Information Processing Systems*, pages 3528–3536, 2015.

[77] Kelvin Xu, Jimmy Ba, Ryan Kiros, Kyunghyun Cho, Aaron Courville, Ruslan Salakhudinov, Rich Zemel, and Yoshua Bengio. Show, attend and tell: Neural image caption generation with visual attention. In *International conference on machine learning*, pages 2048–2057, 2015.

[78] Stephen Merity, Nitish Shirish Keskar, and Richard Socher. Regularizing and optimizing LSTM language models. In *6th International Conference on Learning Representations, ICLR 2018, Vancouver, BC, Canada, April 30 - May 3, 2018, Conference Track Proceedings*, 2018. URL `https://openreview.net/forum?id=SyyGPP0TZ`.

[79] Diederik P. Kingma and Jimmy Lei Ba. Adam: A method for stochastic optimization. In *Proceedings of the 3rd International Conference Learning Representations*, 2014.

[80] Lutz Prechelt. Early stopping-but when? In *Neural Networks: Tricks of the trade*, pages 55–69. Springer, 1998.

[81] Justin Sirignano and Rama Cont. Universal features of price formation in financial markets: perspectives from deep learning. *arXiv preprint arXiv:1803.06917*, 2018.

[82] Olalekan Ogunmolu, Xuejun Gu, Steve Jiang, and Nicholas Gans. Nonlinear systems identification using deep dynamic neural networks. *arXiv preprint arXiv:1610.01439*, 2016.

[83] Michael Collins. Head-driven statistical models for natural language parsing. *Computational Linguistics*, 29(4): 589–637, 2003.

[84] Tadao Kasami. An efficient recognition and syntax-analysis algorithm for context-free languages. *Coordinated Science Laboratory Report no. R-257*, 1966.

[85] Reinhard Kneser and Hermann Ney. Improved backing-off for m-gram language modeling. In *1995 International Conference on Acoustics, Speech, and Signal Processing*, volume 1, pages 181–184. IEEE, 1995.

[86] Stanley F. Chen and Joshua Goodman. An empirical study of smoothing techniques for language modeling. *Computer Speech & Language*, 13(4):359–393, 1999.

[87] Timothy Osborne and Kim Gerdes. The status of function words in dependency grammar: A critique of Universal Dependencies (UD). *Glossa: A journal of general linguistics*, 4(1), 2019.

[88] Richard Futrell, Kyle Mahowald, and Edward Gibson. Quantifying word order freedom in dependency corpora. In *Proceedings of the Third International Conference on Dependency Linguistics (Depling 2015)*, pages 91–100, Uppsala, Sweden, 2015.

[89] Ramon Ferrer i Cancho. Euclidean distance between syntactically linked words. *Physical Review E*, 70:056135, Nov 2004. doi: 10.1103/PhysRevE.70.056135. URL `http://link.aps.org/doi/10.1103/PhysRevE.70.056135`.

[90] Haitao Liu, Chunshan Xu, and Junying Liang. Dependency distance: A new perspective on syntactic patterns in natural languages. *Physics of Life Reviews*, 2017.

[91] David Temperley and Dan Gildea. Minimizing syntactic dependency lengths: Typological/cognitive universal? *Annual Review of Linguistics*, 4:1–15, 2018.

[92] Haitao Liu. Dependency distance as a metric of language comprehension difficulty. *Journal of Cognitive Science*, 9 (2):159–191, 2008.

[93] John A. Hawkins. *A performance theory of order and constituency*. Cambridge University Press, Cambridge, 1994.

[94] John A. Hawkins. *Efficiency and complexity in grammars*. Oxford University Press, Oxford, 2004.

[95] Richard Futrell, Roger Levy, and Edward Gibson. Generalizing dependency distance: Comment on "dependency distance: A new perspective on syntactic patterns in natural languages" by haitao liu et al. *Physics of Life Reviews*, 21:197–199, 2017.

[96] Edward Gibson. Linguistic complexity: Locality of syntactic dependencies. *Cognition*, 68(1):1–76, 1998.

[97] E. Gibson. The dependency locality theory: A distance-based theory of linguistic complexity. In Alec Marantz, Yasushi Miyashita, and Wayne O'Neil, editors, *Image, Language, Brain: Papers from the First Mind Articulation Project Symposium*, pages 95–126, 2000.

[98] Vera Demberg and Frank Keller. Data from eye-tracking corpora as evidence for theories of syntactic processing complexity. *Cognition*, 109(2):193–210, 2008. ISSN 0010-0277. doi: DOI:10.1016/j.cognition.2008.07.008.

[99] Richard L. Lewis and Shravan Vasishth. An activation-based model of sentence processing as skilled memory retrieval. *Cognitive Science*, 29(3):375–419, 2005.

[100] Ramon Ferrer i Cancho and Haitao Liu. The risks of mixing dependency lengths from sequences of different length. *Glottotheory*, 5(2):143–155, 2014.

[101] Susumu Kuno. The position of relative clauses and conjunctions. *Linguistic Inquiry*, 5(1):117–136, 1974.

[102] J. Rijkhoff. Word order universals revisited: The principle of head proximity. *Belgian Journal of Linguistics*, 1: 95–125, 1986.

[103] Lyn Frazier. Syntactic complexity. *Natural language parsing: Psychological, computational, and theoretical perspectives*, pages 129–189, 1985.

[104] J. Rijkhoff. Explaining word order in the noun phrase. *Linguistics*, 28(1):5–42, 1990.

[105] Jason M. Eisner. Three new probabilistic models for dependency parsing: An exploration. In *Proceedings of the 16th Conference on Computational Linguistics*, pages 340–345, 1996.

[106] Holger Diessel. The ordering distribution of main and adverbial clauses: A typological study. *Language*, pages 433–455, 2001.

[107] Matthew Synge Dryer. The positional tendencies of sentential noun phrases in universal grammar. *Canadian Journal of Linguistics/Revue canadienne de linguistique*, 25(2):123–196, 1980.

[108] Matthew S. Dryer and Martin Haspelmath. *WALS Online*. Max Planck Institute for Evolutionary Anthropology, Leipzig, 2013. URL `https://wals.info/`.

Language	ISO Code	Family	Sentences (train/held-out)	Words (train/held-out)
Afrikaans	afr	Germanic	1315/194	30765/4808
Ancient Greek	grc	Greek	26322/2156	323993/33468
Arabic	arb	Semitic	21864/2895	737410/93666
Basque	eus	Basque	5396/1798	61040/20122
Belarusian	bel	Slavic	260/65	4328/1274
Bulgarian	bul	Slavic	8907/1115	106813/13822
Catalan	cat	Romance	13123/1709	375524/50954
Chinese	cmn	Sino-Tibetan	3997/500	85013/10899
Coptic	cop	Egyptian	364/41	8818/871
Croatian	hrv	Slavic	7689/600	148560/12922
Czech	ces	Slavic	102993/11311	1547431/163578
Danish	dan	Germanic	4383/564	69273/8952
Dutch	nld	Germanic	18310/1518	234859/19115
English	eng	Germanic	17062/3070	263328/39537
Estonian	est	Finnic	6959/855	69754/8709
Finnish	fin	Finnic	27198/3239	248283/29204
French	fra	Romance	32347/3232	780289/77416
Galician	glg	Romance	2472/1260	76208/36450
German	deu	Germanic	13814/799	229204/10727
Gothic	got	Germanic	3387/985	35024/10114
Greek	ell	Greek	1662/403	38139/9404
Hebrew	heb	Semitic	5241/484	122122/10050
Hindi	hin	Indic	13304/1659	262389/32850
Hungarian	hun	Ugric	910/441	17282/9974
Indonesian	ind	Malayo-Sumbawan	4477/559	82963/10676
Irish	gle	Celtic	121/445	2864/9554
Italian	ita	Romance	17427/1070	329477/18790
Japanese	jpn	Japanese	7164/511	145240/10404
Korean	kor	Korean	27410/3016	312830/32849
Latin	lat	Latin	30598/2568	387236/29858
Latvian	lav	Baltic	4124/989	51562/10773
Lithuanian	lit	Baltic	153/55	2536/883
Marathi	mar	Indic	373/46	2447/342
Norwegian	nob	Germanic	29870/4639	432741/62802
Old Church Slavonic	chu	Slavic	4123/1073	37432/10100
Persian	pes	Iranian	4798/599	110345/14474
Polish	pol	Slavic	6100/1027	52445/8613
Portuguese	por	Romance	17995/1770	401487/37388
Romanian	ron	Romance	8664/752	170551/14898
Russian	rus	Slavic	52664/7163	773678/105285
Serbian	srp	Slavic	2935/465	57581/8825
Slovak	slk	Slavic	8483/1060	65044/10648
Slovenian	slv	Slavic	7532/1817	106904/22083
Spanish	spa	Romance	28492/3054	731920/79171
Swedish	swe	Germanic	7041/1416	102400/23585
Tamil	tam	Southern Dravidian	400/80	5664/1118
Telugu	tel	South-Central Dravidian	1051/131	3926/519
Turkish	tur	Southwestern Turkic	3685/975	31271/8203
Ukrainian	ukr	Slavic	4506/577	61011/8384
Urdu	urd	Indic	4043/552	103152/13888
Vietnamese	vie	Viet-Muong	1400/800	17325/9873

Table S14: Languages with ISO codes, families (according to `https://universaldependencies.org/`), and the number of available sentences and words.

	Relation	Real	DLM	Efficiency	Expected Prevalence
①	lifted_case				> 50% [1]
②	lifted_cop				> 50% [1]
③	aux				> 50% [1]
④	nmod				> 50% [1]
⑤	acl				> 50% [1]
⑥	lifted_mark				> 50% [1]
⑦	obl				> 50% [1]
⑧	xcomp				> 50% [1]
	advcl				> 50% [6, 106]
	ccomp				> 50% (cf. [107])
	csubj				> 50% (cf. [107])
	nsubj				See Section S1
	amod				≈ 50% [1]
	nummod				≈ 50% [108, 89A, 83A]

Table S15: Predictions on UD relations with predictions from the typological literature (compare Table S7), for languages optimized for Efficiency and Dependency Length Minimization.
